# Profiling polyamine–protein interactions in live cells through photoaffinity labeling

**DOI:** 10.1039/d5cb00103j

**Published:** 2025-09-19

**Authors:** Maciej Zakrzewski, Zuzanna Sas, Benjamin Cocom-Chan, Moh Egy Rahman Firdaus, Marcin Kałek, Karolina Szczepanowska, Piotr Gerlach, Anna Marusiak, Remigiusz A. Serwa

**Affiliations:** a IMol Polish Academy of Sciences M. Flisa 6 02-247 Warsaw Poland r.serwa@imol.edu.pl; b Centre of New Technologies, University of Warsaw S. Banacha 2C 02-097 Warsaw Poland

## Abstract

Polyamines are essential metabolites that play a crucial role in regulating key cellular processes. While previous studies have shown that polyamines modulate protein function through non-covalent interactions, the lack of robust analytical methods has limited the systematic identification of these interactions in living cells. To address this challenge, we synthesized a series of novel photoaffinity probes and applied them to a model cell line, identifying over 400 putative protein interactors with remarkable polyamine analog structure-dependent specificity. Analysis of probe-modified peptides revealed photocrosslinking sites for dozens of protein binders and demonstrated that all but one of the probes, the spermine analog, were intracellularly stable. The interaction profiles of these probes were visualized through in-gel fluorescence scanning, and their subcellular localization was examined using fluorescence microscopy. Spermidine analogs interacted with proteins in the nucleoplasm, colocalizing with nucleolar and nuclear-speckle proteins, as well as in the cytoplasm. By contrast, diamine analogs localized to vesicle-like structures near the Golgi apparatus, implying that different polyamine types exhibit a proclivity for specific cellular compartments. Notably, spermidine analogs bound preferentially to proteins containing acidic stretches, often located within intrinsically disordered regions. Focusing on one such case, we provide *in-cellulo* evidence of direct interactions between G3BP1/2 and spermidine analogs and advance the hypothesis that such interactions influence stress-granule dynamics. Overall, this study provides a comprehensive profile of polyamine analogs–protein interactions in live cells, offering valuable insights into their roles in cellular physiology.

## Abstract 2

Photoaffinity polyamine analogs were synthesized and used to map polyamine–protein interactions in living cells, identifying over 400 protein interactors with structure-dependent specificity.

## Graphical abstract

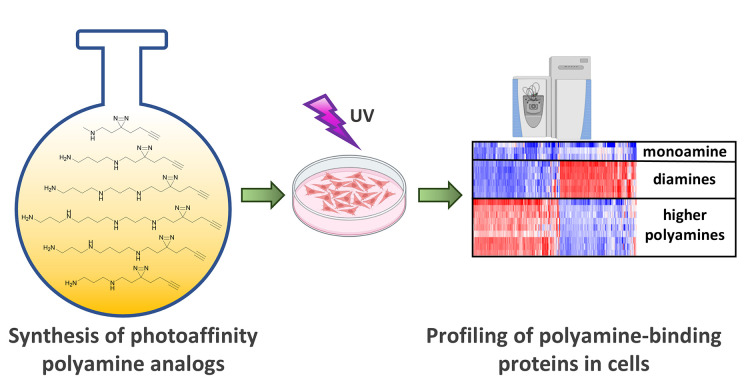

## Introduction

Polyamines (putrescine, spermidine, and spermine) are polycationic molecules essential for regulating cellular processes through interactions with nucleic acids and proteins. These primarily non-covalent interactions are driven by electrostatic forces. While protein-centric studies have identified several polyamine-binding proteins and demonstrated their regulatory effects,^1–3^ direct evidence of specific interactions in cells is limited, largely due to a lack of advanced analytical tools. Investigating the molecular role of polyamines is vital, as they are crucial for cellular health and implicated in pathologies such as cancer and Parkinson's disease.^4–6^

**Scheme 1:**
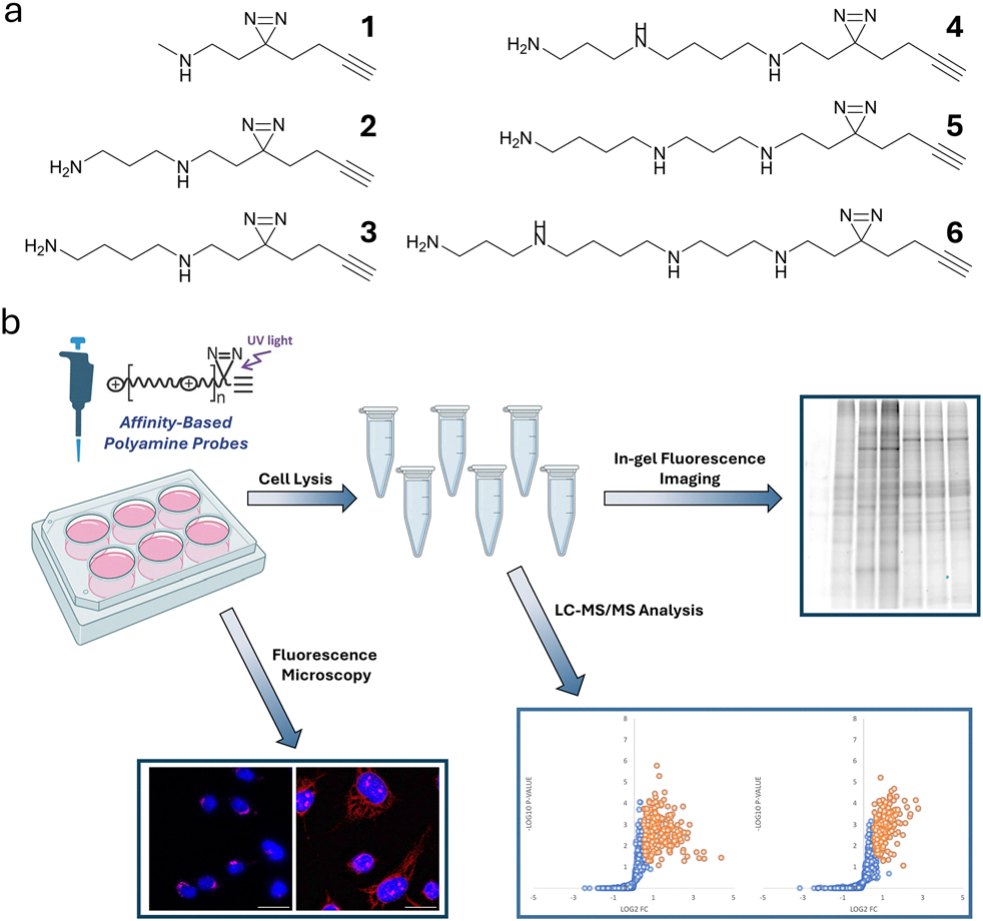
Structures of photoaffinity probes and chemoproteomic workflow for profiling polyamine-binding proteins in live cells. (a) Structures of probes 1–6 (b) The workflow involves treating cells with probes followed by UV irradiation at 365 nm to induce covalent binding to nearby biomolecules. After treatment, cells are lysed, and probe-labeled proteins are ligated to TAMRA or biotin *via* CuAAC. TAMRA-labeled samples are analyzed by SDS-PAGE and in-gel fluorescence imaging, while biotin-labeled samples undergo affinity enrichment on Neutravidin-agarose beads. Enriched proteins are digested with trypsin, labeled with TMT, and analyzed by LC-MS/MS for identification and quantification. Alternatively, following UV irradiation, cells are washed with methanol to precipitate intracellular proteins and remove excess free probe. The precipitated proteins are then reacted with TAMRA *via* CuAAC for subcellular imaging of probe–protein conjugates.

To address this gap, we developed a metabolite-centric methodology using photoaffinity analogs of endogenous polyamines (Scheme 1a). These probes are compatible with mass spectrometry, enabling precise identification of interacting proteins in cells. They also allow detailed analyses of subcellular interactions *via* fluorescent microscopy and SDS-PAGE with in-gel fluorescence imaging (Scheme 1b). This work presents the synthesis and comprehensive evaluation of photoaffinity analogs of putrescine, spermidine, and spermine, and provides a community resource: a catalog of proteins captured by the putrescine and spermidine probes in live eukaryotic cells.

Our study maps the polyamine interaction landscape in a human cell line, uncovering polyamine probe structure-dependent subcellular localization differences and identifying hundreds of protein interactors, including previously known ones.^3,7–9^ The methodology opens new opportunities for studying polyamine dynamics, with implications for cellular biology, disease mechanisms, and therapeutics.

With advances in crosslinking and bioorthogonal chemistry, affinity-based probes have become powerful tools for investigating non-covalent small molecule–protein interactions in complex biological systems, including live cells.^10^ Despite this progress, polyamine-like probes remain underexplored. Early designs from the 1990s were inadequate for unbiased protein identification.^3,11^ More recently, a spermidine analog featuring a bulkier, non-linear and partially aromatic architecture – bearing an aryl diazerine that photolyzes to a short-lived carbene with broad amino-acid reactivity^12^ – was developed by *Singh et al.* for profiling protein interactors.^13^ However, efficient labeling with this probe required prolonged treatment with difluoromethylornithine (DFMO), an inhibitor of ornithine decarboxylase, the rate-limiting enzyme in polyamine biosynthesis. Although DFMO lowers intracellular polyamine levels and enhances probe uptake by activating the polyamine transport system,^14^ it also induces broad cellular changes^15^ that may significantly confound the interpretation of polyamine–protein interactions.

Here, we introduce structurally streamlined analogs of putrescine, spermidine, and spermine (Scheme 1a), each equipped with a “minimalist” alkynyl diazirine photocrosslinker,^16^ designed to better preserve the native properties of the parent compounds while enabling efficient photoaffinity labeling under physiological conditions. Because polyamines are multiply cationic, their principal partners are expected to be protein regions locally enriched in acidic residues. Upon UV irradiation, alkyl diazirines generate a diazo intermediate that preferentially reacts with acidic side chains (*D*/*E*), enabling capture of even weak, transient electrostatic contacts;^12^ this mechanistic complementarity motivated our choice of photocrosslinker.

## Results and discussion

Natural polyamines feature nitrogen atoms, charged at physiological pH, separated by propylene and butylene groups, and our probes retain this spatial charge distribution. Linear polyamine analogs with terminal primary amine modifications enter cells more efficiently than branched polyamines with internal secondary amine modifications.^17^ Therefore, we tagged the terminal amines. Asymmetrical spermidine was tagged at either terminus, N1 (5) or N8 (4). Monoamine 1 was included as a control for amino tag-specific interactions, while diamines 2–3 served as additional controls for higher polyamines 4–6. This probe set was designed to systematically study how structural variations affect protein binding.

Compounds 1–6 were synthesized using a convergent approach involving a “minimalist” affinity tag iodide^18^ and versatile polyamine building blocks featuring a single Nosyl-protected terminal nitrogen atom and Boc-protected remaining nitrogen atoms (Scheme S1). The Nosyl group, acting as both a protecting and activating group, enabled selective alkylation under mild conditions.^19^ This strategy facilitates site-specific introduction of virtually any modification at the activated position. Building blocks for compounds 4–6 were synthesized in 4–6 steps, with overall yields of 56%, 35%, and 13%, respectively. The “minimalist” affinity tag was incorporated *via* nucleophilic substitution, followed by Nosyl group removal, achieving an average yield of 21%. Finally, HCl treatment removed the Boc groups, yielding the desired hydrochloride salts of compounds 1–6 quantitatively.

We initiated the evaluation of compounds 1–6 with photoaffinity experiments performed in cell lysates under non-denaturing conditions (Fig. S1–S3). After incubation with the probes and UV irradiation at 365 nm, protein-probe conjugates were labeled with 5-TAMRA-azide *via* copper-catalyzed azide–alkyne cycloaddition (CuAAC)^20,21^ and analyzed by in-gel fluorescence imaging. Compounds 4–6 produced strong, similar fluorescent patterns, while probe 3 showed weaker, and probes 1–2 much weaker, signals (Fig. S1), indicating reduced protein binding. This suggest that protein interactions of compounds 3–6 rely on additional structural features, as the minimalist photoaffinity tag and 1,3-diaminopropane moiety in compound 2 were insufficient for strong binding. Notably, the fluorescent patterns differed from total protein staining, highlighting selective interactions with specific proteins.

Since both polyamines and proteins bind nucleic acids, we tested whether polyamine analogs preferentially associate with proteins complexed to DNA/RNA, with nucleic acids potentially guiding these interactions. Cell lysates were pre-treated with Benzonase to degrade nucleic acids, and the impact on photoaffinity labeling with probes 3–6 was assessed, revealing that nuclease treatment had little effect on the intensity or profile of protein interactors (Fig. S2).

To confirm the observed probe–protein conjugates resulted from photoaffinity labeling rather than enzymatic ligation (*e.g.* transglutaminase-catalyzed Gln-polyamination),^22^ UV irradiation was omitted before the CuAAC reaction, resulting in negligible fluorescence in the non-photocrosslinked samples (Fig. S3).

Next, competitive affinity labeling experiments were conducted with five amino compounds, including endogenous polyamines. Protein labeling by putrescine (3), spermidine (4), and spermine (6) analogs was reduced in the presence of excess higher polyamines (spermidine and spermine) (Fig. S2). 1,4-Diaminobutane (putrescine) effectively outcompeted its analog 3 and, to a lesser extent, probes 4 and 6, whereas 1,8-diaminooctane had little effect on probes 4 and 6 and no effect on probe 3. The smallest, singly charged dimethylamine did not reduce protein binding to any probe (Fig. S3).

Collectively, these findings underscore the importance of the probes’ potential to form multivalent binding with negatively charged residues on proteins, which appears to play a key role in determining the affinity of these interactions.

**Fig. 1:**
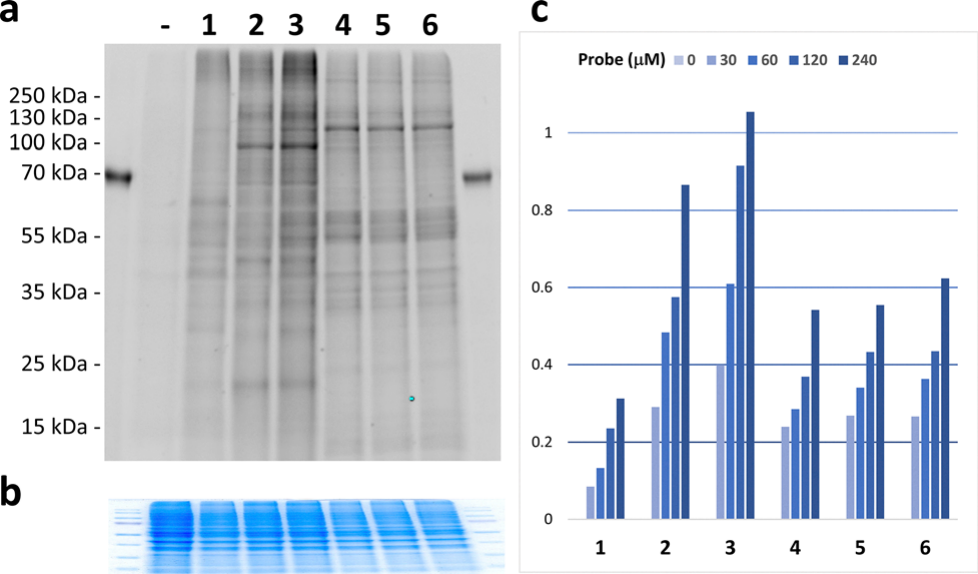
Photoaffinity labeling with polyamine analogs in live cells. HeLa cells were incubated with probes 1–6 (30–240 μM) for 2 h and UV irradiated at 365 nm. probe–protein conjugates were ligated to 5-TAMRA-azide, separated by SDS-PAGE, and visualized *via* in-gel fluorescence imaging. (a) Fluorescent profiles of protein binders in cells treated with vehicle (H_2_O), probe 1 (120 μM), 2 (30 μM), 3 (30 μM), 4 (120 μM), 5 (120 μM), or 6 (120 μM). (b) Total protein stained with Coomassie. To conserve space, the image was horizontally compressed while maintaining accurate representation of relative protein loading across the lanes. (c) Dose-dependent yield of probe–protein conjugate formation, relative to the highest value. Fluorescence signals were normalized to protein load.

Building on the initial testing of the probes in cell lysates, we expanded the study to investigate polyamine-binding proteins in live cells. HeLa cells were incubated with compounds 1–6 at various concentrations, followed by UV irradiation. After lysis, bioorthogonal ligation of the probe–protein conjugates to 5-TAMRA-azide was performed, and fluorescent bands were visualized *via* in-gel fluorescence imaging. This approach revealed both qualitative and quantitative differences in the profiles of interacting proteins, dependent on probe structure (Fig. 1). These differences likely stem from multiple factors, including variations in small molecule import efficiency, intracellular localization, and binding preferences for specific structural motifs in proteins within their natural environment.

Three distinct fluorescent band patterns were observed: one for the monoamine probe (1), another for the diamine probes (2–3), and a third for the higher-polyamine probes (4–6), with some common bands across all conditions (Fig. 1 and Fig. S4). Importantly, no fluorescent bands were detected when UV irradiation was omitted, confirming that the observed probe–protein conjugates were exclusively formed through photoaffinity labeling (Fig. S4).

Given that spermidine is the most extensively studied polyamine – linked to aging, autophagy, and disease modulation across various physiological systems^23^ – we conducted additional mechanistic experiments using its specific analogs, compounds 4 and 5. Photoaffinity labeling with these probes was reduced in the presence of excess natural spermidine, consistent with competition for shared protein targets (Fig. S5). Furthermore, pretreatment with the polyamine transport inhibitor AMXT-1501^24^ reduced labeling efficiency, suggesting that spermidine and compounds 4 and 5 share common transport pathways.

Encouraged by the gel-based results, we conducted a proteomic experiment to identify proteins interacting with the polyamine probes. HeLa cells were supplemented with compounds 1–6 at concentrations yielding comparable labeling efficiencies, UV irradiated, and lysed. The probe–protein conjugates were ligated to azide-PEG_3_-biotin, enriched with Neutravidin-agarose and digested with trypsin to enable the relative quantification of the probe–protein conjugates under different conditions. In total, 413 protein groups were identified as polyamine binders based on statistically significant enrichment in samples from cells supplemented with compounds 2–6 compared to control samples from cells supplemented with 1 (Fig. S6 and Table S1).

In parallel, we investigated the impact of treating HeLa cells with selected probes on total protein levels. These experiments revealed negligible effects on the overall proteomic profile and, in particular, on proteins identified as probe-binding partners (Fig. S7 and Table S2). This finding rules out the possibility that the photoaffinity proteomic results include false positives arising from a hypothetical, probe-induced increase in protein abundance.

The binders of compounds 2–6 included several previously reported polyamine-associated proteins – tubulins,^7^ casein kinase 2,^3^ ribosomal subunits,^8,25^ and the polyamine transporter SLC3A2^9^ – validating the results. Of the polyamine-pathway enzymes expected to interact with the probes, only spermidine synthase (SRM) exhibited binding; for the others, the lack of detection does not allow any inference about binding.

**Fig. 2:**
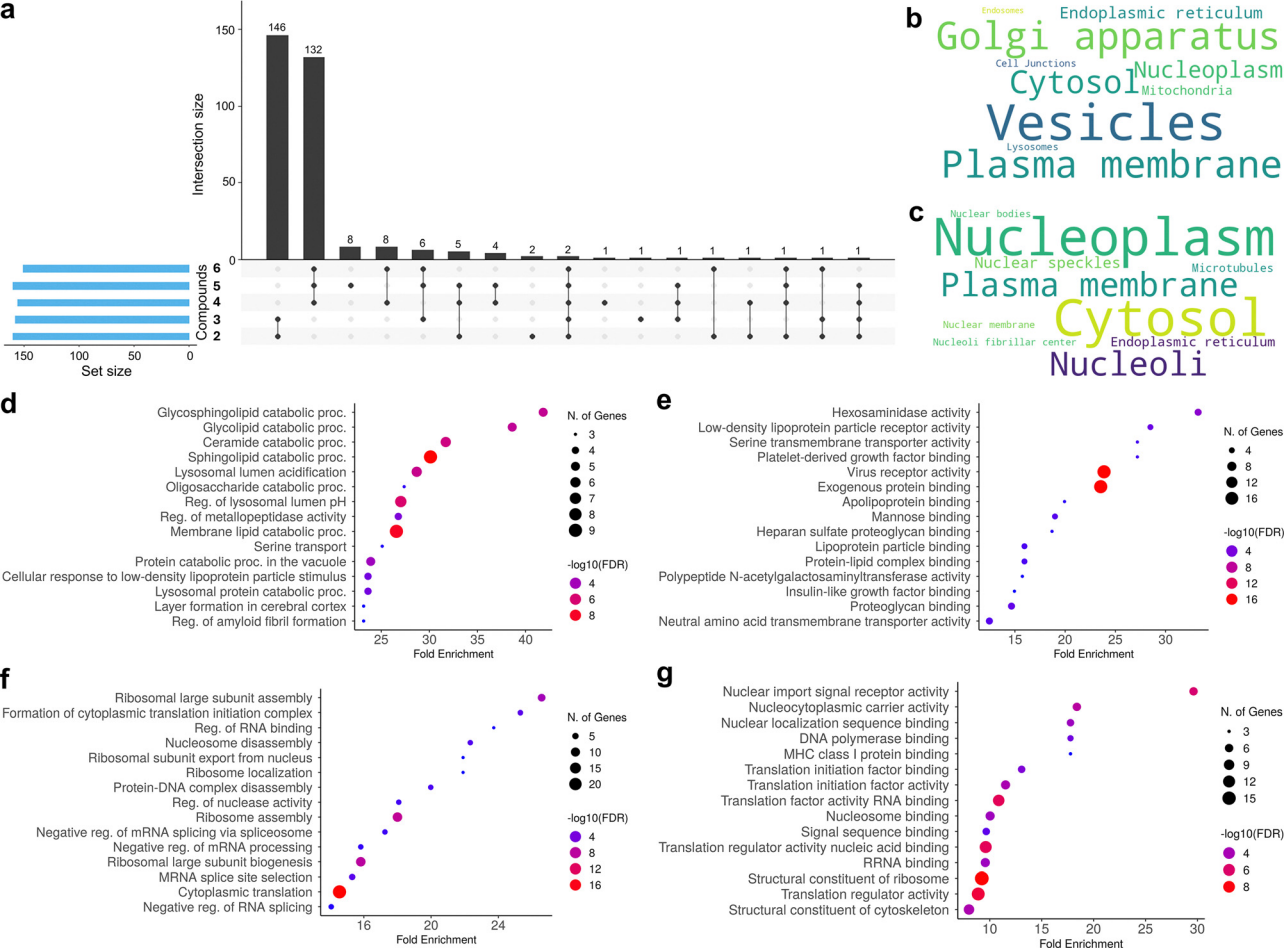
Characterization of protein binders to affinity-based probes in HeLa cells. (a) Matrix layout showing all intersections of compounds 2–6, sorted by intersection size. Dark circles in the matrix indicate sets that are part of each intersection. Set size indicate the number of protein groups bound to each probe. Subset A includes 171 protein groups bound to compounds 2 and/or 3 but not to compounds 4–6, while subset B comprises 195 protein groups bound to compounds 4, 5, and/or 6 but not to compounds 2–3. (b) Text cloud showing the primary subcellular localization of proteins in subset A. (c) Text cloud for proteins in subset B. (d) Top 15 Gene Ontology (GO) Biological Process terms for protein subset A, sorted by fold enrichment. (e) GO Molecular Function terms for subset A. (f) GO Biological Process terms for subset B. (g) GO Molecular Function terms for subset B.

While the total number of binders was similar across the polyamine probes (196 ± 12 protein groups), the identities of proteins attracted to the diamines 2–3 differed substantially from those bound to the longer polyamines 4–6. Specifically, 171 protein groups, subset A (Table S3), were bound to compounds 2 and/or 3 but not to compounds 4–6, while 195 protein groups, subset B (Table S4), were bound to compounds 4, 5, and/or 6 but not to compounds 2–3 (Fig. 2a).

Gene ontology (GO) enrichment analysis revealed distinct molecular functions and biological processes for these subsets (Fig. 2d–g). For example, subset A was enriched for GO terms related to lipid and saccharide processing, binding of extracellular factors, and virus receptor activity, while subset B was associated with nucleic acid processing (replication, transcription, splicing, translation) and nucleocytoplasmic transport. Our proteomic results also imply probe structure-dependent differences in the subcellular trafficking. Proteins binding to probes 2–3 were predominantly localized to vesicles (28%), Golgi apparatus (18%), and plasma membrane (17%), whereas those bound to compounds 4–6 were primarily found in nucleoplasm (26%), cytosol (26%), and nucleoli (7%) (Fig. 2b, c and Tables S3, S4).^26^ Moreover, 16% of subset A proteins, but only 2% of subset B proteins, are predicted to be secreted,^26^ suggesting a specific role for putrescine in associating with proteins during secretion and possibly facilitating this process. Notably, only six mitochondrial proteins were identified as interactors of spermidine analogs 4–5,^26^ in contrast to the study by Singh *et al*. in HEK293 cells, which used a different spermidine probe under polyamine-depleted conditions and reported dozens of mitochondrial targets.^13^ This discrepancy may reflect the effects of prolonged DFMO treatment, which has been shown to compromise mitochondrial ultrastructure and function,^26^ potentially affecting probe uptake, localization, and the resulting interactome profiles. Remarkably, despite differences in cell line and treatment, 51 protein interactors of probes 4–5 overlapped with the other spermidine probe (Fig. S8), providing partial mutual validation of the datasets.

Affinity-based probes enable the investigation of the subcellular distribution of their biomolecular interactors through bioorthogonal ligation with a fluorescent reagent (*e.g.* 5-TAMRA-azide), followed by fluorescence microscopy. The localization of two probes, 3 and 5, chosen for their distinct interactome profiles (Fig. 1a and 2a–c), was examined in HeLa cells after UV irradiation to generate probe-conjugates.

**Fig. 3:**
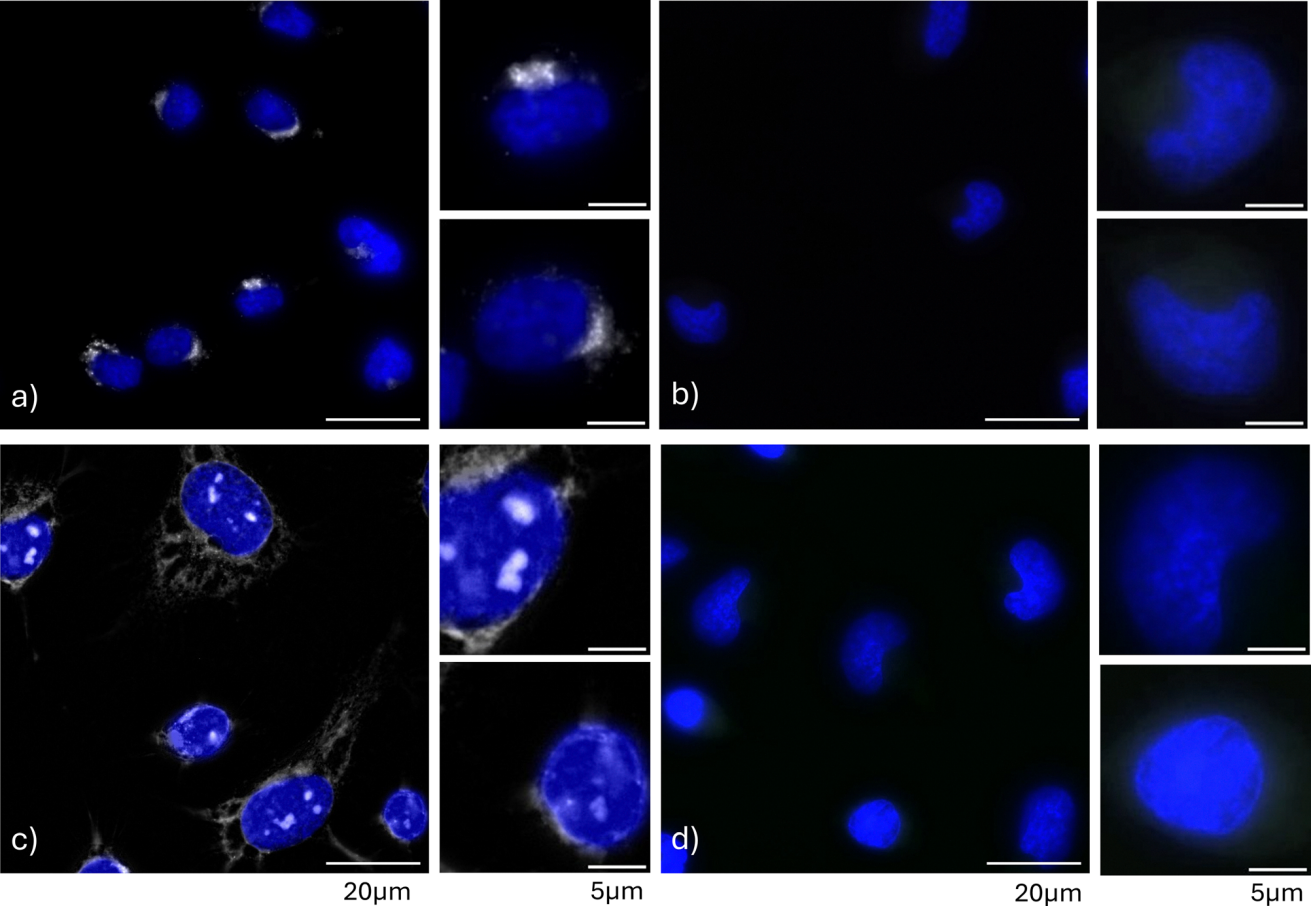
Subcellular localization of putrescine analog 3 and spermidine analog 5 conjugates in HeLa cells. Cells were treated with photoaffinity probe 3 (30 μM) or 5 (200 μM) for 2 h, with or without UV irradiation at 365 nm. The cells were fixed with methanol, and probe conjugates were ligated to 5-TAMRA-azide (grayscale). Nuclei were stained with DAPI (blue) prior to visualization by fluorescence microscopy. (a) Cells treated with probe 3 and subjected to UV irradiation. (b) Cells treated with probe 3 without UV irradiation. (c) Cells treated with probe 5 and subjected to UV irradiation. (d) Cells treated with probe 5 without UV irradiation. Scale bars: 20 μm for wider fields and 5 μm for single cells.

Putrescine analog 3 accumulated in putative vesicular structures near the nucleus (Fig. 3a), while spermidine analog 5 localized to the cytoplasm and nucleoplasm (Fig. 3c), consistent with proteomic data (Fig. 2b and c). Control cells subjected to identical treatments without UV exposure showed no fluorescent signal (Fig. 3b and d). Prompted by proteomic results indicating that probe 3 interacts with numerous Golgi proteins, immunofluorescence staining for GM-130, a Golgi marker, was performed with enhanced image processing^28^ to visualize regions of highest probe conjugate abundance and improve resolution (Fig. S9). The results revealed that putrescine analog 3 closely associated with, but did not penetrate, the Golgi apparatus. This spatial association was maintained during interphase and mitosis (Fig. S10) and showed polar distribution during cell division, as reported previously for the Golgi apparatus.^29^ This suggests a persistent, functional interaction between the Golgi and polyamines-sequestering vesicle-like structures. To determine whether the punctate nuclear pattern observed for the spermidine analog 5 reflects association with proteins in nucleoli and/or nuclear speckles – two subnuclear compartments most prominently annotated among binders in our proteomic screen (Table S4) – we performed colocalization studies using established markers: nucleophosmin (NPM1) for the nucleolar rim and splicing factor 3B subunit 1 (SF3B1) for nuclear speckles. Probe 5 was confined within the ring-like regions delineated by NPM1 staining (Fig. S11) and showed discernible overlap with SF3B1 (Fig. S12), indicating enrichment in both nucleoli and nuclear speckles and supporting the proteomic annotations. While further spatial analysis by immunofluorescence could provide more insights into probe trafficking, it falls outside the scope of this study.

An interesting observation is that 111 out of 195 proteins (57%) in subset B contain at least one 20-amino-acid-long sequence with 10 or more acidic amino acids (*D*/*E*), while only 11 out of 171 proteins (6%) in subset A contain such a motif (Tables S3 and S4). Across the human proteome, 2206 out of 20399 proteins (11%) in the Swiss-Prot database contain this motif. This indicates a strong overrepresentation of acidic stretches in subset B proteins (fold enrichment, FE = 5.3) and a mild underrepresentation in subset A proteins (FE = 0.6). These findings suggest that acidic stretches, often part of intrinsically disordered regions (IDRs), are important for binding proteins to spermidine/spermine probes.

We next performed photoaffinity labeling experiments in HeLa cells applying compounds 3–6 and a modified proteomic workflow. Following bioorthogonal ligation to azide-PEG3-biotin, proteins were digested with trypsin, and the resulting biotinylated, probe-modified peptides were affinity-enriched and analyzed by LC-MS/MS. The search was focused on peptides bearing modifications resulting from azide-PEG3-biotin conjugated to photochemical adducts derived from compound 3 (C_29_H_52_O_5_N_8_S, Δmass = +624.37814 Da), compounds 4–5 (C_32_H_59_O_5_N_9_S, Δmass = +681.43599 Da), and compound 6 (C_35_H_66_O_5_N_10_S, Δmass = +738.49384 Da). A preliminary search (data not shown) revealed that modification sites were predominantly localized to aspartic/glutamic acid (*D*/*E*) within peptides. This observation is consistent with electrostatic guidance by the positively charged polyamine moiety of the probe and aligns with previously reported residue preferences of the “minimalist” alkynyl diazirine photocrosslinker.^12^ A subsequent search focused on *D*/*E* residues identified 334 peptide sequences modified by adducts derived from analogs of putrescine or spermidine – but, surprisingly, none from spermine (Table S5).

A detailed analysis of the dataset revealed that samples from cells treated with analog 3 contained adducts derived from that compound. Similarly, samples from cells treated with analogs 4 and 5 showed adducts corresponding to those respective compounds. In contrast, samples from cells treated with spermine analog 6 contained adducts with masses corresponding to a spermidine analog. Taken together, these observations indicate that whereas analogs 3–5 remained largely intact in cells, analog 6 underwent truncation, plausibly mediated by amine oxidases in the polyamine salvage pathway; this interpretation is consistent with a report that *N*1-butylated spermine is a substrate of human spermine oxidase.^30^ After matching the modified peptide data to the protein-level enrichment data, we mapped 28 sites on 22 putative targets of probes 2–3 (Table S3) and 91 sites on 39 putative targets of probes 4–6 (Table S4) providing additional evidence and another level of detail in characterizing *in cellulo* interactions of polyamine analogs with proteins.

One of the peptides covalently modified by probe 5 mapped to spermidine synthase (SRM) and carried an adduct at E208 position (Fig. S13), consistent with SRM's identification as a binder of that probe in the protein-level enrichment dataset. Modeling and structural superposition of spermidine analog 5 onto spermidine in the previously reported SRM co-crystal structure^31^ indicates that the diazirine of probe 5 lies within reach of the E208 carboxylate, rationalizing the observed modification and supporting a spermidine-like binding pose (Fig. S14). Under this model, both putrescine- and spermidine-based analogs may attempt to align in the amine-acceptor site, with their propargyl group projecting toward the pocket entrance bounded by residues L27, W28, and H213. Notably, presence of the photocrosslinker can introduce steric clashes, potentially impairing or destabilizing native-like binding of the polyamine moiety within this compact cavity. However, the spermidine analog 5 likely has an advantage, with its N8 oriented toward the negatively charged aminopropyl pocket, defined by residues residues D104 and D173. We propose that this additional interaction strengthens electrostatic contacts with SRM, increasing affinity and/or residence time and thereby enabling efficient photocrosslinking at E208. By contrast, the putrescine analog 3 is expected to bind more weakly and transiently, consistent with the absence of detectable crosslinking.

A closer examination of the amino acid sequences of peptides modified by the probes revealed that those photocrosslinked to spermidine analogs are more enriched in acid amino acid residues compared to those photocrosslinked to the putrescine analog. Specifically, 12% (24 out of 198) of peptides exclusively bearing mass adducts corresponding to the spermidine analog contained ≥50% *D*/*E*, and 38% (75 out of 198) contained ≥33% *D*/*E*. In contrast, none (0 out of 47) of the peptides modified exclusively by the putrescine analog contained ≥50% *D*/*E*, and only 13% (6 out of 47) contained ≥33% *D*/*E* (Table S5). These findings are consistent with a higher prevalence of acidic stretches in the protein binders of spermidine compared to those of putrescine, and support the notion that these motifs actively contribute to binding.

Among the handful of proteins for which prior evidence supports polyamine interactions localized to acidic amino acid rich domains are tubulins (*e.g.*, TUBA1C), which interact *via* a C-terminal IDR, and G3BP1, which binds *via* IDR1 (Fig. S15c).^7,32^ While these previous studies supported *in vitro* interaction between higher polyamines and synthetic peptide fragments of tubulins and G3BP1, in this work we present evidence for *in cellulo* interactions, by identifying spermidine probe-modified peptides (Table S5 and Fig. S13) covering precisely the abovementioned regions of proteins: YQDEVFGGFVTEPQEESEEEVEEPEER (133–159) at the N-terminal site of IDR1 in G3BP1 and EDMAALEKDYEEVGADSADGEDEGEEY (423–449) as well as DYEEVGADSADGEDEGEEY (431–449) at the C-terminus of TUBA1C. To complement these, our protein-level enrichment proteomic data identify several tubulins (including TUBA1C) and G3BP2, a paralog of G3BP1 that also contains IDR1 (Fig. S15c), as *in cellulo* binding partners of probes 4–6.

Although interactions between polyamines and tubulins are well documented, their interaction with G3BP1/2 is a recent discovery^32^ that merits further investigation. G3BP1 and G3BP2 are key regulators of stress granules in mammalian cells, promoting the formation of these membraneless organelles essential for survival under adverse conditions.^33^ Accordingly, we used our probes to further investigate G3BP1/2–polyamine interactions, including in the context of stress granule assembly.

Because G3BP1 was not detected in the protein-level enrichment proteomic data, its enrichment status, or the lack of, could not be assessed. We therefore additionally supported the modified peptide data with targeted western blot experiments. Following protein-level enrichment of probe–protein conjugates, elution under denaturing conditions, SDS-PAGE and probing with a G3BP1-specific antibody, we confirmed binding of this protein to compound 4, but not probes 1 or 3 (Fig. S15a), which is consistent with proteomic results for G3BP2 (Fig. S15b) and suggest that both paralogs interact with spermidine in the cell.

**Fig. 4:**
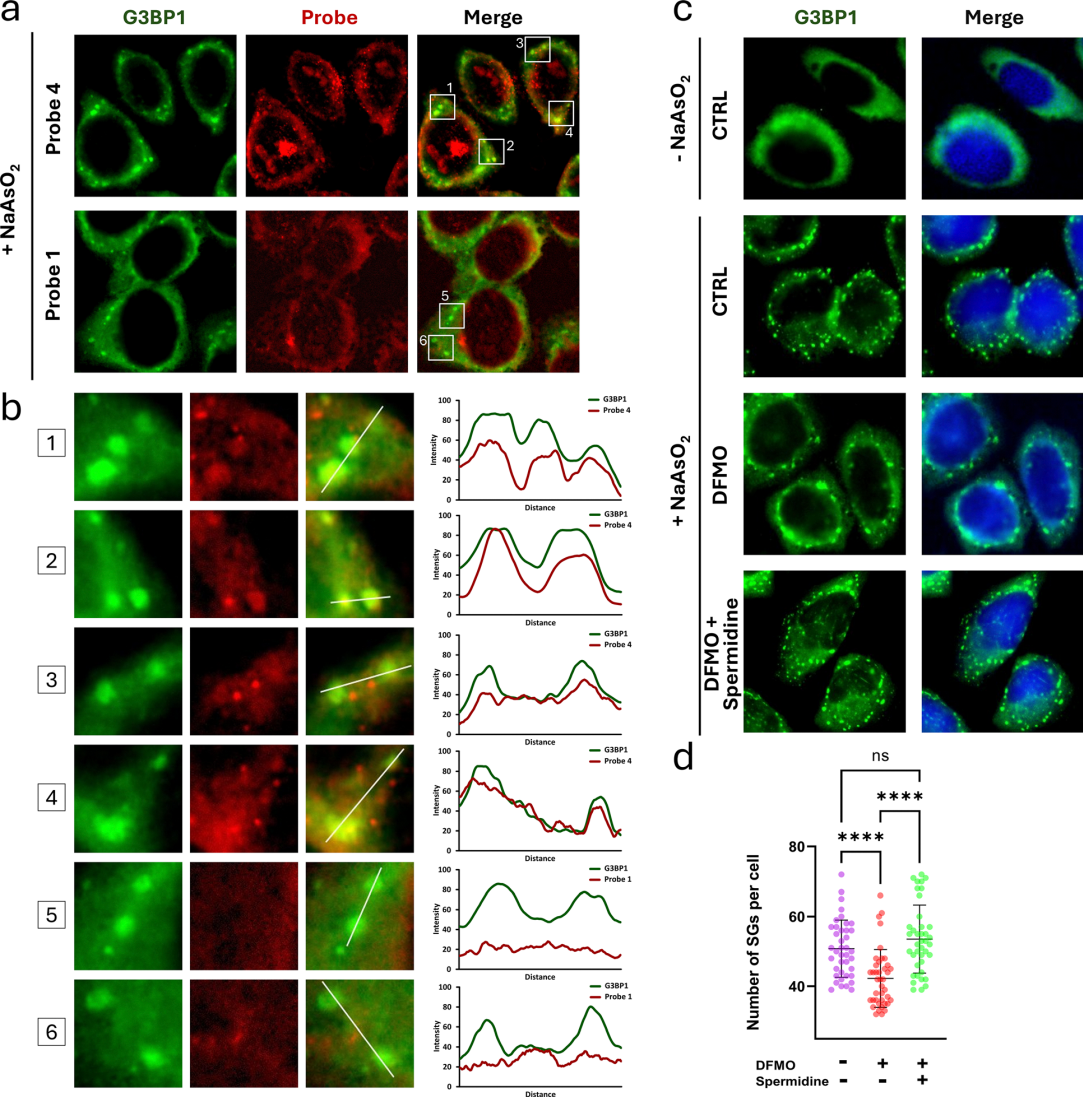
The role of spermidine in the stress granule assembly. (a) Co-localization of G3BP1 protein and probes 4 and 1 in HeLa cells following NaAsO_2_ treatment. Cells were incubated with compound 4 (30 μM) or 1 (100 μM) for 2 h, washed with PBS, and treated with NaAsO_2_ (500 μM) for 30 min to induce stress granule formation. Following UV irradiation at 365 nm, cells were fixed, washed with methanol, and subjected to a CuAAC reaction with 5-TAMRA-azide (red), immunostaining for G3BP1 (green), and visualization by fluorescence microscopy. Images show wider field views (b) magnified regions (indicated in panel a) showing selected stress granules (left) and superimposed signal intensity profiles of G3BP1 and probes (right), obtained in ImageJ Fiji^28^ for points along white lines crossing selected stress granules. (c) Effect of DFMO treatment and spermidine supplementation on stress granule assembly in HeLa cells. Cells were treated with DFMO (2.5 mM) or vehicle (H_2_O) for 48 h, followed by spermidine (500 μM) or vehicle (H_2_O) for 2 h, and then NaAsO_2_ (500 μM) or vehicle (H_2_O) for 30 min. G3BP1-containing stress granules (green) were visualized by fluorescence microscopy after immunostaining. Nuclei were stained with DAPI (blue). Scale bar: 10 μm. (d) Quantification of stress granule numbers in cells (*n* = 40) under different treatment conditions, performed in ImageJ Fiji.^27^ Data show the distribution of stress granule counts in response to DFMO treatment and spermidine supplementation. Statistical significance between groups was assessed by one-way ANOVA.

To assess the interaction of spermidine analog 4 with G3BP1 in stress granules, which may suggest functional relevance of spermidine in the assembly or maintenance of stress granules, co-localization experiments were performed in HeLa cells treated with NaAsO_2_, a strong stress granule inducer.^34^ Monoamine analog 1 served as a control for an incidental, probe-structure-independent trafficking into stress granules. While probe 4 did not co-localize with all stress granules, likely due to competition from intracellular spermidine for G3BP binding, it showed clear co-localization with G3BP1 in many granules (Fig. 4a, b and Fig. S16). In contrast, no visible co-localization was observed for the control compound 1 (Fig. 4a, b and Fig. S16).

To further evaluate the role of spermidine-G3BP1/2 interactions in stress granule assembly, stress granule formation was compared in control cells, polyamine-depleted cells (2.5 mM DFMO, 48 h), and polyamine-depleted cells supplemented with spermidine prior to NaAsO_2_ treatment. Based on prior literature, the polyamine-depletion regimen applied here (2.5 mM DFMO, 48 h) results in undetectable intracellular putrescine and spermidine and a slight reduction in spermine in HeLa cells.^35^ Polyamine depletion significantly impaired stress granule formation, while spermidine supplementation rescued the original phenotype (Fig. 4c and d).

Collectively, our experimental data, together with independent biophysical evidence that polyamines bind the acidic IDR1 of G3BP1 and promote its phase separation *in vitro*,^32^ motivate a testable hypothesis: under NaAsO_2_-induced stress, spermidine engages acidic clusters within IDR1 of G3BP1/2, weakening intramolecular, self-inhibitory contacts between IDR1 and the positively charged IDR3.^36^ This electrostatic engagement would relieve autoinhibition and favor an “open,” RNA-competent conformation, thereby increasing the propensity of G3BP1/2 to phase-separate with RNA and cofactors and facilitating the assembly and/or stabilization of stress granules. This hypothesis is consistent with the reduction of stress granules upon polyamine depletion and their rescue by spermidine supplementation.

## Conclusion

This study presents a comprehensive evaluation of endogenous polyamine-based photoaffinity probes in live cells. By employing a panel of minimalist photoaffinity analogs, we established a robust methodology for identifying protein interactors and mapping their subcellular localization under physiological conditions, without the need for depletion of the endogenous polyamine pool.

In live cells, the probes display distinct protein-binding profiles that are markedly attenuated in lysates, indicating that probe structure-dependent intracellular trafficking is a major determinant of target engagement in a cellular context. Putrescine analogs accumulate in putative vesicular compartments near the Golgi apparatus, engaging nearby proteins, whereas spermidine analogs preferentially bind proteins in the nucleoplasm (notably within nucleoli and nuclear speckles) and in the cytoplasm. Notably, the spermine analog undergoes intracellular catabolism to the corresponding spermidine analog, effectively acting as a catabolically activated spermidine probe that binds protein sets very similar to those captured by the two direct spermidine analogs. These spermidine analogs, regardless of the position of the photocrosslinker substitution (N1 or N8), behave similarly in terms of intracellular stability, localization, and target profiles.

Interestingly, the spermidine probes introduced here capture a partly overlapping yet partly distinct protein repertoire relative to the probe recently reported by Singh *et al.* That study used an aryl-diazirine probe and performed labeling after 48 h DFMO-mediated depletion of endogenous polyamines – a step that reduces competition but shifts cells away from steady state. Their probe also carried N8 branching (aryl diazirine plus alkyne handle), increasing steric bulk relative to the minimalist, linear tags used here. Photochemically, aryl diazirines generate short-lived carbenes with broad amino-acid reactivity, whereas our alkyl diazirines label *via* a diazo intermediate with a reported preference for acidic residues; together with the probes’ cationic character, this favors capture of protein regions locally enriched in acidic residues. Collectively, differences in cellular context (polyamine-depleted vs near-physiological) and probe design/photochemistry (aryl *vs*. alkyl diazirine; branched vs linear) plausibly account for the observed similarities and differences between the two studies; further work will be needed to pinpoint the dominant drivers.

Photoaffinity strategies carry intrinsic caveats that merit attention. The appended photocrosslinker can introduce steric hindrance and misorient the ligand within narrow or compact binding pockets. Consequently, enzymes of the polyamine pathway (sparsely detected here) and other tight-binding polyamine partners residing in constrained cavities may fail to be labeled by polyamine analogs, yielding false negatives despite genuine binding to the natural ligands. In addition, the absence of some expected binders may reflect limitations of untargeted shotgun proteomics, which – despite enabling broad, unbiased profiling – can miss low-abundance proteins, proteins yielding few or suboptimal tryptic peptides, or peptides that co-elute with more abundant species in complex mixtures. Finally, whereas protein-level enrichment affords quantitative comparisons across probes, peptide-level enrichment is not quantitative; consequently, individual modified peptides can arise from low-stoichiometry, potentially incidental events. We therefore recommend interpreting peptide-level identifications primarily for proteins that also show enrichment at the protein level, either by mass spectrometry or by western blot.

Protein binding to the probes is governed by the number and spatial arrangement of charged nitrogen atoms, with triamines exhibiting stronger binding affinities and a pronounced preference for proteins enriched in acidic stretches compared with diamines. This underscores the role of multivalent electrostatic interactions in determining target specificity.

While studying electrostatic interactions between intrinsically disordered regions (IDRs) in proteins and small molecules in the complex environment of living cells is inherently challenging, the methodology described here enables direct detection of such interactions, including identification of the binding site. One such interaction examined in greater detail was between spermidine and the acidic IDR1 of G3BP1/2 proteins, key regulators of stress granule formation. Here, we present a direct *in cellulo* evidence for this interaction, which was previously characterized *in vitro* using orthogonal methodologies. Our data further suggest that it also occurs within stress granules and may contribute to their formation or stabilization under stress conditions, as stress granule abundance was significantly reduced under polyamine-depleted conditions but restored following supplementation with extracellular spermidine.

In summary, we introduce novel polyamine analogs and map their protein interactors in live cells under near-physiological conditions, yielding a resource intended to help build a more comprehensive picture of the cellular roles of polyamines.

## Author contributions

Conceptualization: RAS. Methodology development: RAS and MZ. Organic synthesis: MZ and MK. Proteomic data processing and analysis: RAS. Fluorescence microscopy: ZS, BC, MERF, KS, AM, and PG. Funding acquisition: RS. Supervision: RS. Writing: RS with help of other authors.

## Conflicts of interest

There are no conflicts to declare.

## Supplementary Material

CB-OLF-D5CB00103J-s001

CB-OLF-D5CB00103J-s002

## Data Availability

The authors confirm that the data supporting the findings of this study are available within the article and its supplementary information (SI). Supplementary information: the tables containing the list of proteins or peptides were compiled in a separate Excel file and added as SI. The datasets containing synthesis procedures, NMR spectra, gels, blots, and microscopy images supporting this article have been uploaded as SI. In addition, mass spectrometry data have been deposited in the ProteomeXchange Consortium^37^*via* the PRIDE partner repository^38^ under the dataset identifiers PXD058706 (HeLa cell protein interactome of polyamine affinity-based probes), PXD066655 (HeLa cell protein interactome of polyamine affinity-based probes (probe-modified peptides)) and PXD062452 (Shotgun analysis of proteins in HeLa cells following treatment with polyamine affinity-based probes). Raw imaging data have been deposited https://doi.org/10.5281/zenodo.16925272. See DOI: https://doi.org/10.1039/d5cb00103j.
