# Supplementary material for: Profiling polyamine–protein interactions in live cells through photoaffinity labeling
Source: RSC Chem Biol. 2025 Sep 19;6(11):1787–96. doi: 10.1039/d5cb00103j (PMC12461607; doi:10.1039/d5cb00103j)
Supplement: CB-006-D5CB00103J-s002 [file CB-006-D5CB00103J-s002.pdf]

Supplementary Information (SI) for RSC Chemical Biology.
This journal is © The Royal Society of Chemistry 2025

Supplementary information

Profiling Polyamine-Protein Interactions in Live Cells Through Photoaffinity Labeling

Authors:

Maciej Zakrzewski,[1] Zuzanna Sas,[1] Benjamin Cocom-Chan,[1] Moh Egy Rahman Firdaus,[1] Marcin Kałek,[2] Karolina Szczepanowska,[1] Piotr Gerlach,[1] Anna Marusiak,[1] Remigiusz A. Serwa[1,*]

[1] IMol, Polish Academy of Sciences, M. Flisa 6, 02-247 Warsaw, Poland

[2] Centre of New Technologies, University of Warsaw, S. Banacha 2C, 02-097, Warsaw, Poland

* For correspondence: r.serwa@imol.edu.pl

Contents:

1. Supplementary methods (experiments in cells and cell lysates)

2. Synthetic procedures and Scheme S1

3. Figures S1-16

4. Tables S1-5

5. References

1. Supplementary methods (experiments in cells and cell lysates)

1.1. General

Unless stated otherwise, all reagents were purchased from Sigma Aldrich or Thermo Fisher Scientific. Human cervical cell carcinoma line HeLa was purchased from ATCC and routinely tested for mycoplasma infection (Universal Mycoplasma Detection Kit, ATCC).

1.2. HeLa cell culture

HeLa cells were grown in complete medium composed of Dulbecco's Modified Eagle Medium (DMEM) supplemented with 10% fetal bovine serum (FBS) and 1% penicillin/streptomycin (P/S) in a humidified 5% CO_2-containing atmosphere at 37 °C. Cells were plated 24 h before treatments. To generate naïve cell lysates, the cells were washed with phosphate-buffered saline (PBS), detached with trypsin, washed with PBS (2×), and the resulting cell pellets were immediately deep-frozen and stored at -80 °C.

1.3. Treatment of HeLa cell lysates with photoaffinity probes **1**-**6**

Lysates were prepared by suspending HeLa cell pellets in a non-denaturing lysis buffer containing 25 mM Tris (pH 7.4), 150 mM NaCl, 5% (m/v) glicerol, 1% (m/v) dodecyl-β-D-maltoside (DDM), and 1× cOmplete EDTA-free Protease Inhibitor Cocktail (Roche). Protein concentrations in lysates were determined using the DC Protein Assay (Bio-Rad) and ranged from 1.2 to 1.6 mg/ml, depending on the batch. For comparative experiments, a single batch of lysate was aliquoted and applied uniformly across all samples. Lysate samples (100 µg of protein at 1 mg/ml) were incubated with probes **1**-**6** (25, 50, 100, or 200 µM) for 15 min at 4 °C. For competitive affinity-based labelling experiments, lysates were pre-incubated with 10 mM amino-compounds (dimethylamine, putrescine, 1,8-diaminooctane, spermidine, or spermine) or a vehicle control (water) for 15 min at 4 °C, followed by incubation with photoaffinity probes (50 µM) for 15 minutes at 4 °C. It was confirmed that the addition of the competitors did not alter the pH of the lysate solutions. For experiments involving Benzonase nuclease, lysates were pre-incubated for 30 min at 25 °C with 30 units of enzyme (E1014, Millipore) in enzyme dilution buffer (50 mM Tris-HCl, 20 mM NaCl, 2 mM $MgCl_2$) or with dilution buffer alone. Then, upon cooling to 4 °C, the lysates were incubated with photoaffinity probes (50 µM) for 15 min at 4 °C. Following incubation with probes, the samples were immediately irradiated (365 nm, 10+ mW/cm2) for 10 min using an LED light reaction box (WaveyTech Ltd, London, UK). Control samples were not UV irradiated, to exclude covalent probe-protein binding not related to the photoaffinity labelling. Proteins were precipitated by addition of methanol (1 vol.) and chloroform (1/4 vol.), followed by a brief vortex-mixing. The pellets were washed with methanol twice, air-dried, and reconstituted in 2% SDS in PBS. Cysteine residues were reacted with 40 mM iodoacetamide for 30 min to prevent azide-alkyne-thiol reaction.[1] Proteins were precipitated as described above, dissolved in 2% SDS in PBS (30 µl), then diluted with PBS (90 µl) and subjected to the CuAAC ligation as described below.

1.4. Treatment of HeLa cells with photoaffinity probes **1**-**6**

The complete medium was removed, cells were washed with PBS and incubated with compounds **1**-**6** (30-240 µM) in FBS-free DMEM supplemented with 1% P/S for 2 h.

For competition experiments, the cells were washed with PBS and incubated with spermidine (2.5 mM) in FBS-free DMEM supplemented with 1% P/S for 2h. Then compound **4** or **5** (25 µM) was added and the cells were incubated 1h.

For experiments involving inhibition of the polyamine transport system, the cells were threated overnight with AMXT-1501 (2 µM) in complete media. Then cells were washed with PBS, compound **4** or **5** (75 µM) and fresh portion of AMXT-1501 (2 µM) were added in FBS-free DMEM supplemented with 1% P/S for 1h.

The medium was removed, cells were washed with PBS and immediately irradiated (365 nm, 10+ mW/cm^2) for 10 min in PBS using an LED light reaction box (WaveyTech Ltd, London, UK). Control cells were not UV irradiated, to exclude covalent probe-protein binding not related to the photoaffinity labelling. The cells were lysed in 2% SDS in PBS, probe sonicated (3 pulses of 5 s), and cysteine residues were reacted with 40 mM iodoacetamide for 30 min to prevent azide-alkyne-thiol reaction.[1] Proteins were precipitated by addition of methanol (1 vol.) and chloroform (1/4 vol.), followed by a brief vortex-mixing. The pellets were washed with methanol twice, air-dried, and reconstituted in 2% SDS in PBS. The solutions were diluted with PBS to the final concentration of 0.5% SDS and the protein concentration was determined by DC Protein Assay (Bio-Rad). Protein concentrations were adjusted to 1 mg/ml using 0.5% SDS in PBS, and the samples were subjected to CuAAC ligation as described below.

1.5. CuAAC ligation

A click reaction mixture was prepared by combining 4 reagents (volumes given per 100 µg of proteins): 5-TAMRA-azide (Jena Bioscience, 1 µl, stock 10 mM in DMSO, final conc. 0.1 mM) or azido-PEG$_3$-biotin (Thermo Fisher Scientific, 1 µl, stock 20 mM in water, final conc. 0.2 mM), $CuSO_4$ (2 µl, stock 50 mM in water, final conc. 1 mM), tris(2-carboxyethyl)phosphine (TCEP) (2 µl, stock 50 mM in water, final conc.1 mM), tris((1-hydroxy-propyl-1H-1,2,3-triazol-4-yl)methyl)amine (THPTA) (1 µl, stock 20 mM in water, final conc. 0.2 mM). Click mixture (6 µl) was added to protein samples (100 µg). The samples were vortex-mixed at RT for 1 h. EDTA (final conc. 5 mM), methanol (1 vol.), and chloroform (1/4 vol.) were added to each sample. The samples were quickly vortexed and centrifuged at 21,000 *g* for 5 min to pellet precipitated proteins. The pellets were washed with methanol twice and dried.

1.6. SDS-PAGE and in-gel fluorescence imaging

The pellets were dissolved in 45 µl of sample loading buffer cont. 10% β-mercaptoethanol, boiled for 5 min, centrifuged at briefly, and proteins separated by SDS-PAGE (12% polyacrylamide gels) using a Mini-PROTEAN system (Bio-Rad). The fluorescence image was recorded using Amersham Imager (Amersham). Subsequently the total protein was stained by Coomassie Brilliant Blue (CBB) and imaged using Epson Perfection V850 Pro scanner (Epson). The fluorescence and CBB signal saturation levels were quantified using ImageJ Fiji software.[2] Saturation profiles were integrated over horizontal slices of the gel, and total signal levels for the lanes were obtained by summing the slice signals. Identical slice positioning was applied to both fluorescence and CBB signals. Finally, fluorescence signals were normalized to total protein loading.

1.7. Protein pull-down and western blot analysis

The pellets were resuspended in 2% SDS in PBS and PBS was added to dilute the sample (final protein concentration 1 mg ml^{-1}, 0.5% SDS). Streptavidin magnetic beads (New England Biolabs) were washed with 0.5% SDS in PBS (3×). The sample was added to the beads and the enrichment was carried out for 2 h at RT with 1100 RPM shaking. The supernatant was removed and the beads washed with 0.5% SDS in PBS (3×), then with 0.6 M aq. NaCl, and the bound proteins were eluted by boiling in the sample loading buffer. Following SDS-PAGE separation, the proteins were transferred onto polyvinylidene difluoride (PVDF) membrane. The membrane was cut based on the protein ladder indications and incubated with 5% powdered milk in tris-buffered saline containing 0.1% Tween 20 (TBST). The membrane was then subjected to sequential incubation with G3BP1-specific recombinant rabbit monoclonal antibody (1:100, MA5-35425, Invitrogen), a horseradish peroxidase-conjugated goat anti-rabbit secondary antibody (1:10000, 31460, Invitrogen), and a chemiluminescent substrate (A38554, Thermo Fisher Scientific). The chemiluminescence image was recorded with Amersham Imager (Amersham).

1.8. Protein pull-down and proteomic analysis

The pellet was resuspended in 2% SDS in PBS and PBS was added to dilute the sample (final concentration 1 mg ml^{-1}, 0.5% SDS). NeutrAvidin agarose resin was washed with 0.5% SDS in PBS (3×). The samples were added to the beads and the enrichment was carried out for 2 h at RT with 1100 RPM shaking. The supernatants were removed and the beads were washed with 0.5% SDS in PBS (2×), with PBS (2×), with 0.6 M aq. NaCl, and then with 100 mM HEPES pH 8.0 (2×). The beads were suspended in 100 mM HEPES pH 8.0 containing 5 mM TCEP and 10 mM chloroacetamide. Sequencing Grade Modified Trypsin (Promega) was added and the samples incubated overnight at 37 °C with 1100 RPM shaking. The samples were centrifuged and the supernatants transferred into clean tubes. The solutions were acidified with trifluoroacetic acid (TFA, to final conc. 1%) and tryptic peptides were labelled using an on-column TMT labelling protocol.[3] TMT-labelled peptides were compiled into a single TMT-18 sample and concentrated using a SpeedVac concentrator. Prior to the LC-MS/MS measurements, the peptide sample was resuspended in 0.1% TFA and 2% acetonitrile in water. The sample was injected twice. Chromatographic separation was performed on an Easy-Spray Acclaim PepMap column (50 cm length × 75 µm inner diameter, Thermo Fisher Scientific) at 55 °C by applying 180 min acetonitrile gradients in 0.1% aqueous formic acid at a flow rate of 300 nl/min. An UltiMate 3000 nano-LC system was coupled to a Q Exactive HF-X mass spectrometer via an easy-spray source (all Thermo Fisher Scientific). The Q Exactive HF-X was operated in TMT mode with survey scans acquired at a resolution of 60,000 at m/z 200. Up to 18 of the most abundant isotope patterns with charges 2-5 from the survey scan were selected with an isolation window of 0.7 m/z and fragmented by higher-energy collision dissociation with normalized collision energies of 32, while the dynamic exclusion was set to 40 s. The maximum ion injection times for the survey scan and dual MS (MS/MS) scans (acquired with a resolution of 45,000 at m/z 200) were 50 and 130 ms, respectively. The ion target value for MS was set to 3e6 and for MS/MS was set to 1e5, and the minimum AGC target was set to 8e2. The data were processed with MaxQuant v. 1.6.17.0 and the peptides were identified from the MS/MS spectra searched against Uniprot Human Reference Proteome (UP000005640)[4] using the built-in Andromeda search engine.[5] Reporter ion MS2-based quantification was applied with reporter mass tolerance = 0.003 Da and min. reporter PIF = 0.75. Cysteine carbamidomethylation was set as a

fixed modification and methionine oxidation, glutamine/asparagine deamidation as well as protein N-terminal acetylation were set as variable modifications. For in silico digests of the reference proteome, cleavages of arginine or lysine followed by any amino acid were allowed (trypsin/P), and up to two missed cleavages were allowed. The FDR was set to 0.01 for peptides, proteins and sites. Since two raw files were collected (double injection of the sample), match between runs was enabled. Other parameters were used as pre-set in the software. Reporter intensity corrected values for protein groups were loaded into Perseus v. 1.6.10.[6] Standard filtering steps were applied to clean up the dataset: reverse (matched to decoy database), only identified by site, and potential contaminant (from a list of commonly occurring contaminants included in MaxQuant) protein groups were removed. Reporter intensity corrected values were log2 transformed and protein groups with values across all samples were kept. The values were normalized by median subtraction within TMT channels. To determine which proteins were bound to the probes **2**-**6**, Student's t-tests (1-sided, permutation-based FDR = 0.05, S0 = 0.5, n = 3) were performed on two groups of samples: samples derived from cells treated with one of the probes **2**-**6** vs samples derived from cells treated with the control compound **1**. The table was exported from Perseus and formatted to its final form (Table S1) in Microsoft Excel 2016.

1.9. Determination of total protein levels in extracts from cells subjected to photoaffinity labeling.

The treatment of HeLa cells with photoaffinity probes (**1**, **3**, **4**, and **6**) or vehicle control (H_2O) and the initial steps of cell lysate preparation were performed as described in section 1.4. Protein pellets were resuspended in 100 mM HEPES pH 8.0 containing 5 mM TCEP and 10 mM chloroacetamide, and subjected to overnight digestion at 37°C with sequencing grade modified trypsin (1 µg : 100 µg total protein). The samples were acidified with TFA to the final concentration of 1 % and centrifuged at 21,000 x g for 3 min at RT. Tryptic peptides (10 µg per sample) were then desalted and TMT-labeled on StageTip, compiled into a single TMT sample, and concentrated using a SpeedVac concentrator. Peptides in the compiled sample were fractionated (8 fractions) using the Pierce™ High pH Reversed-Phase Peptide Fractionation Kit (Thermo Fisher Scientific). Prior to LC-MS measurement, peptide fractions were resuspended in 0.1% TFA, 2% acetonitrile in water. Chromatographic separation was performed on an Easy-Spray Acclaim PepMap column 50 cm long × 75 µm inner diameter (Thermo Fisher Scientific) at 55 °C by applying a 180 min acetonitrile gradients in 0.1% aqueous formic acid at a flow rate of 300 nl/min. An UltiMate 3000 nano-LC system was coupled to a Q Exactive HF-X mass spectrometer via an easy-spray source (all Thermo Fisher Scientific). The Q Exactive HF-X was operated in TMT mode with survey scans acquired at a resolution of 60,000 at m/z 200. Up to 15 of the most abundant isotope patterns with charges 2-5 from the survey scan were selected with an isolation window of 0.7 m/z and fragmented by higher-energy collision dissociation (HCD) with normalized collision energies of 32, while the dynamic exclusion was set to 35 s. The maximum ion injection times for the survey scan and the MS/MS scans (acquired with a resolution of 45,000 at m/z 200) were 50 and 150 ms, respectively. The ion target value for MS was set to 3e6 and for MS/MS to 1e5, and the minimum AGC target was set to 1e3. The data were processed with MaxQuant v. 2.6.7.0, and the peptides were identified from the MS/MS spectra searched against Uniprot Human Reference Proteome (UP000005640)[4] using the build-in Andromeda search engine.[5] Reporter ion MS2-based quantification was applied with reporter mass tolerance = 0.003 Da and min. reporter PIF = 0.75. Cysteine carbamidomethylation was set as a fixed modification and methionine oxidation, glutamine/asparagine deamination, as well as protein N-terminal acetylation were set as variable

modifications. For in silico digests of the reference proteome, cleavages of arginine or lysine followed by any amino acid were allowed (trypsin/P), and up to two missed cleavages were allowed. The FDR was set to 0.01 for peptides, proteins and sites. Match between runs was enabled. Other parameters were used as pre-set in the software. Reporter intensity corrected values for protein groups were loaded into Perseus v. 1.6.10.[6] Standard filtering steps were applied to clean up the dataset: reverse (matched to decoy database), only identified by site, and potential contaminant (from a list of commonly occurring contaminants included in MaxQuant) protein groups were removed. Reporter intensity corrected values were log2 transformed and protein groups with values across all samples were kept. The values were normalized by median subtraction within TMT channels. To determine proteins differentially regulated by polyamine probes **3**, **4**, and **6**, compared to either the control compound **1** or the vehicle control, Student's t-tests (2-sided, permutation-based FDR = 0.01, S0 = 0.1, n = 3). The table was exported from Perseus and formatted to its final form (Table S2) in Microsoft Excel 2016.

1.10. Bioinformatic analysis

Gene Ontology (GO) functional protein enrichment analyses were performed using the web-based tool ShinyGO 0.81[7] on two protein subsets. Subset A (n = 171) consisted of proteins bound to compounds **2** and/or **3** but not to compounds **4**-**6**. Subset B (n = 195) included proteins bound to compounds **4**, **5**, and/or **6** but not to compounds **2**-**3**. Both subsets were analyzed against the human proteome. GO molecular function and biological process terms were limited to top 15 results. The false discovery rate (FDR) cutoff for multiple hypothesis testing was set to 0.01, with a minimal pathway size of 5 and a maximal pathway size of 500. Enriched pathways and terms were ranked based on fold enrichment. To identify acidic stretches in proteins, a FASTA file containing the amino acid sequences of the revised human proteome (UniProtKB) was downloaded from the UniProt Knowledgebase.[4] This file was split into 10 text files, each containing approximately 2,000 entries. The files were uploaded into the OpenAI ChatGPT-4o environment, where the tool was queried to identify 20-amino-acid-long sequence fragments containing at least 10 acidic amino acids (D/E) along with the corresponding protein identifiers. These identifiers were matched to Subsets A and B from the proteomic analysis. For validation, 20 randomly selected identifiers from Subset B were manually checked, confirming 100% accuracy in the assignment of acidic stretches. Text clouds were generated using ChatGPT-4o. Protein identifiers obtained from the proteomic analysis were matched to the Human Protein Atlas subcellular localization dataset[8] using Perseus software.[6] This table was then uploaded into the ChatGPT-4o environment, where text clouds were generated to represent the frequency of subcellular localization for proteins in Subset A (diamines) and Subset B (higher polyamines). The plot representing the matrix layout for the intersections of protein bound to photoaffinity probes was produced in R using the UpSetR package.[9]

1.11. Probe-modified peptide pull-down and proteomic analysis

The pellet was suspended in 100 mM HEPES pH 8.0 containing 5 mM TCEP and 10 mM chloroacetamide. Sequencing Grade Modified Trypsin (Promega) was added and the samples incubated overnight at 37 °C with 1100 RPM shaking. EDTA-free Protease Inhibitor Cocktail (1x, Roche) was added to quench residual trypsin activity. NeutrAvidin agarose resin was washed with 100 mM HEPES (3×). The tryptic peptide samples were centrifuged and clear supernatants added to the beads.

The enrichment was carried out for 2 h at RT with 1100 RPM shaking. The supernatants were removed and the beads were washed with 100 mM HEPES (3×), with water (2×), and with 10% acetonitrile in water (2×). Probe-modified peptides were eluted at 37 °C with 0.1% trifluoroacetic acid and 1% formic acid in water (2×15 min). Combined eluates were concentrated using a SpeedVac concentrator. The samples were pre-cleaned using C18 StageTips and resuspended in 0.1% TFA and 2% acetonitrile in water prior to the LC-MS/MS measurements. Chromatographic separation was performed on an Easy-Spray Acclaim PepMap column (50 cm length × 75 µm inner diameter, Thermo Fisher Scientific) at 55 °C by applying 90 min acetonitrile gradients in 0.1% aqueous formic acid at a flow rate of 300 nl/min. An UltiMate 3000 nano-LC system was coupled to a Q Exactive HF-X mass spectrometer via an easy-spray source (all Thermo Fisher Scientific). The Q Exactive HF-X was operated in TMT mode with survey scans acquired at a resolution of 60,000* or 120,000 at m/z 200. Up to 12 or 15* of the most abundant isotope patterns with charges 2-7 or 3-6* from the survey scan were selected with an isolation window of 1.3 m/z and fragmented by higher-energy collision dissociation with normalized collision energies of 27, while the dynamic exclusion was set to 30 or 35* s. Optimized parameters are marked with asterisk. The maximum ion injection times for the survey scan and dual MS (MS/MS) scans (acquired with a resolution of 15,000 or 30,000 at m/z 200) were 45 and 150 ms, respectively. The ion target value for MS was set to 3e6 and for MS/MS was set to 1e5, and the minimum AGC target was set to 1e3. The data were processed with MaxQuant v. 1.6.17.0 and the peptides were identified from the MS/MS spectra searched against Uniprot Human Reference Proteome (UP000005640)[4] using the built-in Andromeda search engine.[5] Cysteine carbamidomethylation was set as a fixed modification, while azido-PEG$_3$-biotin ligated to photochemical adducts on glutamic or aspartic acid residues - derived from compounds **3** ($C_{29}H_{52}O_5N_8S$, mass increase = 624.37814), **4-5** ($C_{32}H_{59}O_5N_9S$, mass increase = 681.43599), and **6** ($C_{35}H_{66}O_5N_{10}S$, mass increase = 738.49384) - were set as variable modifications. For in silico digests of the reference proteome, cleavages of arginine or lysine followed by any amino acid except proline were allowed (trypsin), and up to two missed cleavages were allowed. Min. peptide length was wet to 0. The FDR was set to 0.01 for peptides, proteins and sites. Min. score and delta score for modified and unmodified peptides were set to 0. Match between runs was disabled. Other parameters were used as pre-set in the software. Intensity values from the modificationSpecificPeptides table were loaded into Perseus v. 1.6.10.[6] Standard filtering steps were applied to clean up the dataset: reverse (matched to decoy database) and potential contaminant (from a list of commonly occurring contaminants included in MaxQuant) peptides were removed. Intensity values were log$_2$-transformed, and unique (Groups) peptides with a posterior error probability (PEP) < 0.001 that contained the above-specified mass increases and were detected in at least 2 out of 3 replicates were retained. Modified peptide data were exported from Perseus and formatted to its final form (Table S5) in Microsoft Excel 2016. Next, intensity values from modification sites tables were loaded into Perseus v. 1.6.10.[6] Standard filtering steps were applied to clean up the dataset: reverse (matched to decoy database) and potential contaminant (from a list of commonly occurring contaminants included in MaxQuant) peptides were removed. Intensity values were log$_2$-transformed, and unique (Groups) peptides with a posterior error probability (PEP) < 0.001, modification site localization probability > 0.5, and detected in at least 2 out of 3 replicates were retained and matched to the lists of proteins assigned as polyamine probe binding partners based on the protein-based enrichment experiment. The data were exported from Perseus and formatted to its final form (Tables S3 and S4) in Microsoft Excel 2016.

1.12. Fluorescent microscopy imaging

HeLA cells were cultured in DMEM with 10% FBS and 1% pen-strep on coverslips until 60-80% of confluence. For the difluoromethylornithine (DFMO) sets of experiments, the cells were pre-exposed to 2.5 mM DFMO or vehicle control for 48 h. The cells were washed with PBS, incubated in serum-free Opti-MEM media containing polyamine analogs (30-200 µM), spermidine (500 µM) or vehicle controls for 2 h, and the medium was removed. For stress-granule formation experiments the cells were incubated with 500 µM $NaAsO_2$ or vehicle control (H_2O) for 30 min. The cells were washed with PBS and immediately irradiated (365 nm, 10+ mW/cm^2) for 10 min in PBS using an LED light reaction box (WaveyTech Ltd, London, UK). Control cells were not UV irradiated. Fixation and permeabilization was performed using pre-chilled methanol for 10 min at -20 °C followed by additional washes with methanol and PBS (2×) at RT. The coverslips were incubated for 1h at RT in the dark with a diluted click reaction mixture prepared by combining 4 reagents: 5-TAMRA-azide (1 µl, stock 10 mM in DMSO), $CuSO_4$ (2 µl, stock 50 mM in water), TCEP (2 µl, stock 50 mM in water), THPTA (1 µl, stock 20 mM in water) and PBS (100 µl). The coverslips were blocked with 3% BSA in PBS. For the Golgi marker immunostaining the samples were incubated for 1 h with a primary anti-GM130 antibody (1:250, ab52649, Abcam) followed by 1 h incubation with a secondary antibody Goat Anti-Rabbit IgG CoraLite®488-Conjugated (1:1000, Proteintech). For the nuclear speckles immunostaining the samples were incubated overnight with a primary anti-SF3B1 antibody (1:1000, #172634, Abcam) followed by 1 h incubation with a secondary antibody Goat Anti-Rabbit IgG CoraLite®488-Conjugated (1:1000, Proteintech). For the nucleoli immunostaining the samples were incubated overnight with a primary anti-NPM1 antibody (1:200, #32-5200, Invitrogen) followed by 1 h incubation with a secondary antibody Goat Anti-Mouse IgG CoraLite®488-Conjugated (1:1000, Proteintech). For the stress granule experiments, the samples were incubated for 1 h with G3BP1 Recombinant Rabbit Monoclonal Antibody (1:100, MA5-35425, Invitrogen), followed by 1 h incubation with Donkey anti-Rabbit IgG (H+L) Highly Cross-Adsorbed Secondary Antibody conjugated to Alexa Fluor Plus 488 (1:2000, Thermo Fisher Scientific). The samples were stained with Hoechst 33342 before mounting or mounted using ProLong™ Diamond Antifade Mountant with DAPI (Thermo Fisher Scientific). The samples were then imaged on an Axio Observer microscope (Zeiss). The samples stained for the nuclear speckles were imaged on confocal microscope LSM710 NLO (Zeiss). Fluorescence images were processed and analyzed using FIJI ImageJ software (version 1.54k). Raw image files were imported, and channels were adjusted to the same exposure settings for both experimental and control groups to ensure consistency in fluorescence intensity comparisons. Brightness and contrast adjustments were applied equally across all images and channels within a particular dataset. For an easier appreciation of figures S8 and S9, deconvolution was performed using the Iterative Deconvolve 3D plugin in Fiji.[2] Green (488) and red (TAMRA) fluorescence channels were deconvolved separately using a theoretical Point Spread Function (PSF) generated using the Diffraction PSF 3D plugin with parameters matching the numerical aperture (NA) of the microscope objective and the emission wavelength of the fluorophores. Stress granules were analyzed as described above without deconvolution steps. For quantifying the number of stress granules per cell, the images were converted to binary using a calculated thresholding weighted mean method and analyzed further using previously described pipeline,[10] adjusting parameters to fit foci as granules. Obtained regions of interest (ROI) were quantified from 40 cells per condition. Statistical analysis was performed using GraphPad Prism (GraphPad Software). One-way ANOVA was employed to assess statistical significance between groups, and differences were considered significant at $p<0.05$. Pearson correlations were quantified using Fiji plugin JACoP.[11]

2. Synthetic procedures and Scheme S1

2.1. General

All materials were purchased from commercial suppliers and used without further purification. Thin layer chromatography (TLC) was performed on Merck Silica gel 60 F254 precoated aluminium sheets. Compounds were visualized by UV at 254 nm, or by staining with ninhydrin or aqueous $KMnO_4$. Column chromatography was conducted using SiliaFlash Irregular Silica Gel P60 (40-63 µm, 60 A). NMR spectra were recorded using Agilent 400 MHz or Bruker Avance 500 MHz spectrometer at ambient temperature. Chemical shifts are reported in parts per million (ppm) and referenced against the residual proton or carbon signal of solvent: $CDCl_3$ (1H = 7.26 ppm, 13C = 77.16 ppm), D_2O (1H = 4.79 ppm). Coupling constants (J) are given in hertz (Hz). Data are reported as follows: chemical shift, multiplicity, coupling constant, and integration. HRMS data were obtained using Q-Exactive HF-X mass spectrometer (Thermo Fisher Scientific).

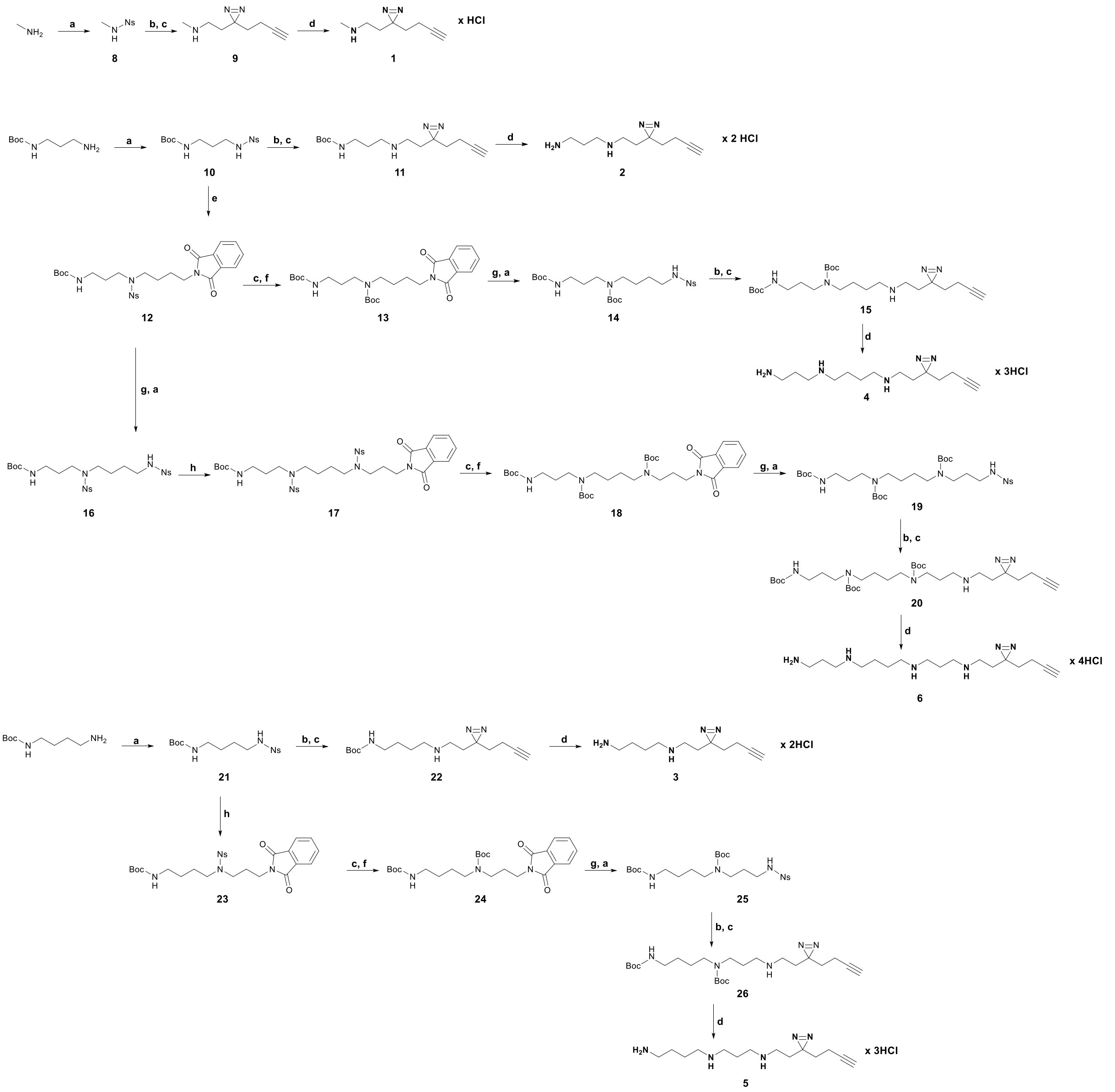

Scheme S1. The synthesis of polyamine analogs **1-6.** Reagents: **a.** NsCl, Et_3N; **b.** Cs_2CO_3, **7** (3-(but-3-yn-1-yl)-3-(2-iodoethyl)-3H-diazirine); **c.** Cs_2CO_3, thiophenol; **d.** 4M HCl/Dioxan; **e.** Cs_2CO_3, TBAI, N-(4-Bromobutyl)phthalimide; **f.** $(BocO)_2O$, NaOH; **g.** N_2H_4; **h.** Cs_2CO_3, TBAI, N-(3-Bromopropyl)phthalimide.

2.2. Preparation of basic building blocks

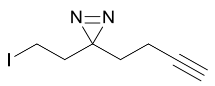

7

Compound **7** was synthesized following the procedures previously described.[12] This seven-step synthesis provided the product as a colorless oil with an overall yield of 23%. The spectra collected during the synthesis were consistent with previously reported data. **^{1}H NMR (400 MHz, $CDCl_3$)** δ 2.89 (t, J=8Hz, 2H), 2.12 (t, J=8Hz, 2H), 2.05-2.01 (m, 3H), 1.69 (t, J=7.2Hz, 2H).

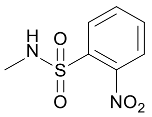

8

3.5 g (15.8 mmol) of 2-nitrobenzenesylfonyl chloride (NsCl) was dissolved in 100 mL of dichloromethane (DCM) and cooled to 0 °C in an ice-water bath. Then 5 mL of 33% methylamine (53.2 mmol) in EtOH was added dropwise. The reaction was stirred at 0 °C for 1h. The reaction mixture was diluted with DCM and washed twice with 1M HCl. The organic layer was dried over $MgSO_4$ and concentrated under reduced pressure. The residue was purified by column chromatography (silica, 40% AcOEt/hexanes). The product was obtained as a white solid (2.87 g, 84%). **^{1}H NMR** (500 MHz, $CDCl_3$) δ 8.15-8.13 (m, 1H), 7.88-7.86 (m, 1H), 7.76-7.75 (m, 2H), 5.22 (bs, 1H), 2.79 (d, J=5Hz, 3H); **^{13}C NMR** (126 MHz, $CDCl_3$) δ 148.43, 133.81, 132.83, 132.62, 131.65, 125.59, 29.92.

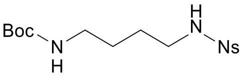

21

To a solution of N-Boc-1,4-butanediamine (3.0 g, 15.96 mmol) in DCM (100 mL), 4.38 mL (31.5 mmol) of triethylamine was added and the mixture was cooled to 0 °C in an ice-water bath. Next, 2.8 g (12.72 mmol) of 2-nitrobenzenesylfonyl chloride was added portionwise. The reaction mixture was stirred overnight at room temperature. Subsequently, the reaction mixture was diluted with DCM and washed twice with 1M HCl. The organic layer was dried over $MgSO_4$ and concentrated under reduced pressure. The product was obtained as a yellowish solid 5.88 g (99%); **TLC** (50% EtOAc/ hexanes): Rf=0.38; **^{1}H NMR (500 MHz, $CDCl_3$)** 8.13-8.11 (m, 1H), 7.86-7.84 (m, 1H), 7.75-7.73 (m, 2H), 5.38 (bs, 1H), 4.55 (bs, 1H), 3.12-3.07 (m, 1H), 1.58-1.48 (m, 4H), 1.42 (bs, 9H); **^{13}C NMR (126 MHz, $CDCl_3$)** δ 156.12, 148.21, 133.77, 133.71, 132.93, 131.20, 125.52, 79.39, 43.55, 39.94, 28.51, 27.27, 27.00.

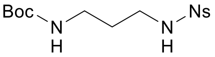

10

To a solution of N-Boc-1,3-propanediamine (2.0 g, 11.5 mmol) in DCM (80 mL), 3.2 mL (23.0 mmol) of triethylamine was added and the mixture was cooled to 0 °C in an ice-water bath. Next, 3.05 g (13.8 mmol) of 2-nitrobenzenesylfonyl chloride was added portionwise. The reaction mixture was stirred overnight at room temperature. Subsequently, the reaction mixture was diluted with DCM and washed twice with 1M HCl. The organic layer was dried over $MgSO_4$ and concentrated under reduced pressure. The product was obtained as a yellowish solid 4.03 g (97%); **TLC** (50% EtOAc/ hexanes): Rf=0.29; **^{1}H NMR (500 MHz, $CDCl_3$)** δ 8.12-8.11 (m, 1H), 7.84-7.83 (m, 1H), 7.73-7.71 (m, 2H),5.91 (bs, 1H), 4.71 (bs, 1H), 3.22-3.12 (m, 4H), 1.71-1.65 (m, 2H), 1.41 (bs, 9H); **^{13}C NMR (126 MHz, $CDCl_3$)** δ 156.61, 148.19, 134.08, 133.60, 132.82, 130.97, 125.39, 79.69, 40.93, 37.22, 30.69, 28.46.

2.3. Procedure 1 - substitution of Nosyl-protected amines with N-(Bromoalkyl)phthalimide

Nosyl-protected amine **10**, **16** or **21** (1 equiv.) was dissolved in anhydrous acetonitrile (10 mL/1.5 mmol) and cesium carbonate (1 equiv.), tetrabutylammonium iodide (TBAI, 1 equiv.), and either N-(3-bromopropyl)phthalimide or N-(4-bromobutyl)phthalimide (1 equiv.) were added. The reaction mixture was stirred overnight at 60 °C. After completion, the solvent was removed under reduced pressure, and the residue was partitioned between DCM and water. The aqueous layer was extracted with DCM twice. The combined organic layers were dried over $MgSO_4$, filtered, and concentrated

under reduced pressure. The crude product was purified by column chromatography (silica, AcOEt/hexanes).

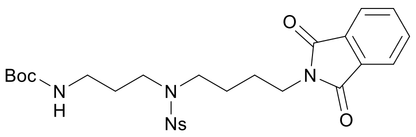

12

Compound **12** was prepared following Procedure 1 through a reaction between compound **10** (2.0 g, 5.57 mmol) and N-(4-bromobutyl)phthalimide. The product was obtained as a yellowish solid 2.91 g (93%); **TLC** (70% EtOAc/ hexanes): Rf=0.48; **^{1}H NMR** (500 MHz, $CDCl_3$) δ 8.00-7.98 (m, 1H), 7.86-7.83 (m, 2H), 7.75-7.71 (m, 2H), 7.69-7.65 (m, 2H), 7.59-7.57 (m, 1H), 4.93 (bs, 1H), 3.65 (m, 2H), 3.37-3.33 (m, 4H), 3.16-3.15 (m, 2H), 1.76-1.74 (m, 2H), 1.67-1.64 (m, 2H), 1.58-1.55 (m, 2H), 1.44 (bs, 9H). **^{13}C NMR** (126 MHz, $CDCl_3$) δ 168.42, 156.11, 148.07, 134.13, 133.60, 133.41, 132.06, 131.79, 130.63, 124.27, 123.35, 79.27, 47.10, 45.31, 37.45, 37.16, 28.64, 28.50, 25.75, 25.45.

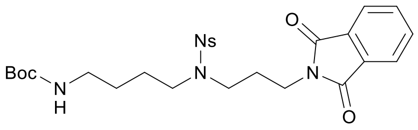

23

Compound **23** was prepared following Procedure 1 through a reaction between compound **21** (1.56 g, 4.20 mmol) and N-(3-bromopropyl)phthalimide. The product was obtained as a yellowish solid 2.19 g (93%); **TLC** (70% EtOAc/ hexanes): Rf=0.55; **^{1}H NMR** (500 MHz, $CDCl_3$) δ 7.97-7.95 (d, J=8Hz, 1H), 7.86-7.81 (m, 2H), 7.73-7.69 (m, 2H), 7.68-7.62 (m, 2H), 7.59 (d, J=8Hz, 1H), 4.65 (bs, 1H), 3.67 (m, 2H), 3.39-3.31 (m, 4H), 3.12-3.08 (m, 2H), 1.94-1.88 (m, 2H), 1.64-1.58 (m, 2H), 1.51-1.49 (m, 2H), 1.41 (bs, 9H); **^{13}C NMR** (126 MHz, $CDCl_3$) δ 168.32, 156.12, 148.16, 134.19, 133.64, 133.37, 132.12, 131.78, 130.81, 124.32, 123.44, 79.23, 47.59, 45.43, 40.03, 35.55, 28.53, 27.47, 27.29, 25.82.

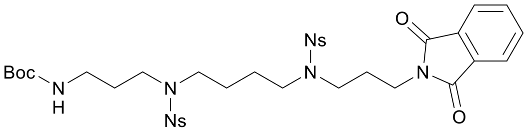

17

Compound **17** was prepared following Procedure 1 through a reaction between compound **16** (0.6 g, 0.98 mmol) and N-(3-bromopropyl)phthalimide. The product was obtained as a yellowish solid 0.59 g (75%); **TLC** (70% EtOAc/ hexanes): Rf=0.38; **^{1}H NMR** (500 MHz, $CDCl_3$) δ 7.96-7.92 (m, 2H), 7.83-7.80 (m, 2H), 7.72-7.57 (m, 8H), 4.90 (bs, 1H),3.63 (t, 2H, J=7Hz), 3.34-3.27 (m, 8H), 3.13-3.10 (m, 2H), 1.89-1.84 (m, 2H), 1.74-1.69 (m, 2H), 1.54-1.48 (m, 2H), 1.41 (bs, 9H); **^{13}C NMR** (126 MHz, $CDCl_3$) δ 168.33, 156.14, 148.08, 134.19, 133.76, 133.29, 133.18, 132.07, 131.93, 131.89, 130.71, 130.65, 124.34, 124.32, 123.42, 79.28, 77.41, 47.04, 46.91, 45.27, 45.17, 37.49, 35.45, 28.65, 28.51, 27.42, 25.20, 24.99.

2.4. Procedure 2 - replacement of Nosyl protecting groups with Boc protecting group

Compound **12**, **17** or **23** was dissolved in acetonitrile (10 mL/1.5 mmol), then cesium carbonate (3 equiv.) and thiophenol (2.0 equiv.) were added. The reaction mixture was stirred overnight at 60 °C. Next, di-tert-butyl dicarbonate (1.5 equiv.) was added, and the reaction mixture was stirred overnight at room temperature. The solvent was then removed under reduced pressure. The residue was partitioned between DCM and water, and the aqueous layer was extracted with DCM twice. The combined organic layers were dried over $MgSO_4$, filtered, and concentrated under reduced pressure. The crude product was purified by column chromatography (silica, AcOEt/hexanes).

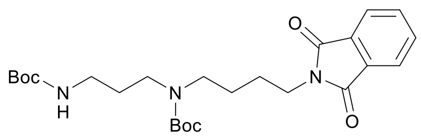

13

Compound **13** was prepared from **12** (2.91 g, 5.20 mmol) following Procedure 2. The product was obtained as a yellowish solid 1.84 g (75%); **TLC** (60% EtOAc/ hexanes): Rf=0.68; **^{1}H NMR** (500 MHz, $CDCl_3$) δ 7.85-7.83 (m, 2H), 7.72-7.70 (m, 2H), 5.29 (bs, 1H), 3.69 (t, 2H, J=7Hz), 3.24-3.15 (bm, 4H), 3.08 (bs, 2H), 1.68-1.61 (m, 4H), 1.58-1.49 (m, 2H), 1.42 (bs, 18H)^{13}C NMR (126 MHz, $CDCl_3$) δ 168.51, 156.21, 147.81, 147.65, 134.17, 134.10, 132.20, 132.14, 123.43, 123.40, 123.36, 85.11, 47.35, 45.37, 37.33, 28.55, 27.59, 25.93, 25.87, 25.79, 24.79.

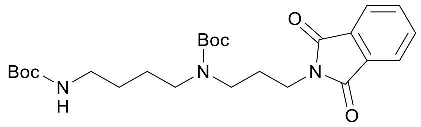

24

Compound **24** was prepared from **23** (2.63 g, 4.70 mmol) following Procedure 2. The product was obtained as a yellowish solid 1.41 g (63%); **TLC** (60% EtOAc/ hexanes): Rf=0.65; **^{1}H NMR (500 MHz, $CDCl_3$)** δ 7.83-7.81 (m, 2H), 7.70-7.68 (m, 2H), 4.67 (bs, 1H), 3.66 (t, J=10Hz, 2H), 3.24-3.16 (m, 4H), 3.12-3.08 (m, 2H), 1.91-1.85 (m, 2H), 1.54-1.49 (m, 2H), 1.46-1.43 (m, 2H), 1.40 (bs, 18H); **^{13}C NMR (126 MHz, $CDCl_3$)** δ 168.38, 156.08, 155.54, 134.05, 132.18, 123.32, 79.57, 79.09, 46.94, 45.15, 40.31, 35.93, 28.51, 28.48, 28.06, 27.47, 25.80.

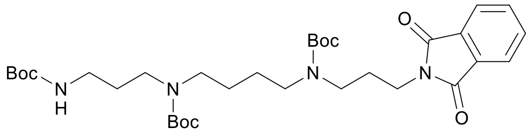

18

Compound **18** was prepared from **17** (2.29 g, 2.85 mmol) following Procedure 2. Two protecting groups were exchanged simultaneously using twice the stoichiometric amount of reagents. The product was obtained as a yellowish solid 1.03 g (57%); **TLC** (60% EtOAc/ hexanes): Rf=0.54; **^{1}H NMR** (500 MHz, $CDCl_3$) δ 7.82-7.80(m, 2H), 7.70-7,68 (m, 2H), 4.81 (bs, 1H), 3.66 (m, 2H), 3.23-3.06 (m, 10H), 1.91-1.85 (m, 2H), 1.47-1.45 (m, 6H), 1.42 (bs, 27H); **^{13}C NMR (126 MHz, $CDCl_3$)** δ 168.37, 156.19, 155.49, 134.06, 132.16, 123.31, 79.56, 78.95, 77.36, , 46.88, 45.03, 43.80, 37.73, 37.42, 35.90, 28.53, 28.47, 28.05, 27.58, 25.89.

2.5. Procedure 3 - replacement of Phthalimide protecting group with Nosyl protecting group

In a round-bottom flask equipped with a condenser, compound **12**, **13**, **18** or **24** was dissolved in ethanol (20 mL/1.0 mmol), and hydrazine dihydrate (4.0 equiv.) was added. The reaction mixture was gently heated overnight at 80 °C in an oil bath. After cooling, the resulting white precipitate was filtered off and washed several times with DCM. The solvent was evaporated under reduced pressure. The residual oil was dissolved in DCM (10 mL/1.5 mmol), and triethylamine (2.0 equiv.) was added. The mixture was cooled down to 0 °C in an ice-water bath, and 2-nitrobenzenesylfonyl chloride (1.5 equiv.) was added portionwise. The reaction mixture was stirred overnight at room temperature. It was then diluted with DCM and washed twice with 1M HCl. The organic layer was dried over $MgSO_4$ and concentrated under reduced pressure. The crude product was purified by column chromatography (silica, AcOEt/hexanes).

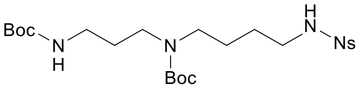

14

Compound **14** was prepared from **13** (1.84 g, 3.87 mmol) following Procedure 3. The product was obtained as a yellowish solid 1.69 g (82%); **TLC** (60% EtOAc/ hexanes): Rf=0.31; **^{1}H NMR (500 MHz, $CDCl_3$)** δ 8.11-8.09 (m, 1H), 7.84-7.82 (m, 1H), 7.73-7.72 (m, 2H), 5.50 (bd, 1H), 4.72 (bs, 1H), 3.17-3.05 (m, 8H), 1.61 (bs, 2H), 1.53-1.49 (m,4H), 1.41 (bs, 18H); **^{13}C NMR (126 MHz, $CDCl_3$)** δ 156.08, 148.18, 133.78, 133.69, 132.87, 131.10, 125.44, 79.79, 77.41, 46.34, 45.99, 44.37, 43.73, 43.56, 37.53, 29.01, 28.51, 28.34, 27.10, 25.66, 25.13.

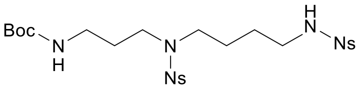

16

Compound **16** was prepared from **12** (5 g, 8.93 mmol) following Procedure 3. The product was obtained as a yellowish solid 2.45 g (45%); **TLC** (60% EtOAc/ hexanes): Rf=0.28; **^{1}H NMR (500 MHz, $CDCl_3$)** δ 8.13-8.10 (m, 1H), 7.99-7.97 (m, 1H), 7.87-7.84 (m, 1H), 7.77- 7.73 (m, 2H), 7.72-7.68 (m, 2H), 7.63-7.61 (m, 1H), 5.32 (bs, 1H), 4.79 (bs, 1H), 3.33- 3.26 (m,4H), 3.13-3.08 (m, 4H), 1.73-1.71 (m, 2H), 1.61-1.58 (m, 2H), 1.55-1.51 (m, 2H), 1.43 (bs, 9H); **^{13}C NMR (126 MHz, $CDCl_3$)** δ 156.18, 148.21, 148.17, 133.82, 133.71, 133.31, 133.03, 131.95, 131.20, 130.75, 125.59, 124.40, 79.48, 47.11, 45.37, 43.20, 37.58, 28.80, 28.55, 26.70, 25.17.

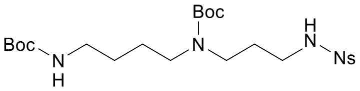

25

Compound **25** was prepared from **24** (0.82 g, 1.72 mmol) following Procedure 3. The product was obtained as a yellowish solid 0.55 g (60%); **TLC** (60% EtOAc/ hexanes): Rf=0.30; **^{1}H NMR (500 MHz, $CDCl_3$)** δ 8.11-8.09 (bs, 1H), 7.81 (bs, 1H), 7.73-7.70 (bs, 2H), 6.38 (bs, 1H). 4.55 (bs, 1H), 3.26 (bs, 2H), 3.12-3.07 (m, 6H), 1.71-1.67 (m, 2H), 1.51-1.46 (m, 4H), 1.42 (bs, 18H); **^{13}C NMR (126 MHz, $CDCl_3$)** δ 156.40, 156.10, 148.15, 133.40, 132.63, 130.76, 125.14, 79.99, 79.20, 77.36, 46.85, 43.44, 40.76, 40.18, 28.68, 28.49, 28.45, 27.47, 25.82.

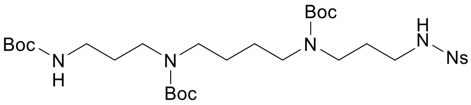

19

Compound **19** was prepared from **18** (1.46 g, 2.3 mmol) following Procedure 3. The product was obtained as a yellowish solid 1.22 g (77%); **TLC** (60% EtOAc/ hexanes): Rf=0.38; **^{1}H NMR (500 MHz, $CDCl_3$)** δ 8.11-8.09 (bs, 1H), 7.85-7.81 (bs, 1H), 7.75-7.70 (bs, 2H), 6.37 (bs, 1H), 4.71 (bs, 1H), 3.23-3.06 (m, 12H), 1.68-1.57 (m, 8H), 1.41 (bs, 27H); **^{13}C NMR (126 MHz, $CDCl_3$)** δ 156.19, 148.22, 133.70, 133.40, 132.63, 131.16, 130.82, 125.50, 125.19, 80.03, 46.93, 46.37, 43.86, 43.55, 40.81, 37.50, 28.56, 28.51, 26.11.

2.6. Procedure 4 – alkylation with photoaffinity tag **7** and removal of Nosyl protecting group

Compound **8**, **10**, **14**, **19**, **21** or **25** was dissolved in anhydrous acetonitrile (10 mL/1.0 mmol). Cesium carbonate (1.5 equiv.) and **7** (1.0 equiv.) were added, and the reaction mixture was stirred overnight at 60 °C. Thiophenol (2.0 equiv.) was then added, and the reaction mixture was stirred at 60 °C for 1.5 h. The solvent was removed under reduced pressure, and the residue was partitioned between DCM

and water. The organic layers were dried over $MgSO_4$, filtered, and concentrated under reduced pressure. The crude product was purified by column chromatography (silica, MeOH/DCM).

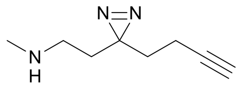

9

Compound **9** was prepared from **8** (580 mg, 2.68 mmol) following Procedure 4. The product was obtained as a pale-yellow oil 40 mg (10%); **TLC** (5% MeOH/DCM): Rf=0.18; **^{1}H NMR (400 MHz, $CDCl_3$)** δ 3.52 (bs, 1H), 2.42-2.39 (m, 5H), 1.98-1.95 (m, 3H), 1.70-1.67 (m, 2H), 1.62 (m, 2H); **^{13}C NMR (101 MHz, $CDCl_3$)** δ 82.73, 69.36, 69.11, 49.71, 45.94, 40.27, 35.81,13.41.

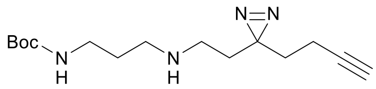

11

Compound **11** was prepared from **10** (250 mg, 0.70 mmol) following Procedure 4. The product was obtained as a pale-yellow solid 45 mg (22%); **TLC** (5% MeOH/DCM): Rf=0.25; **^{1}H NMR (400 MHz, $CDCl_3$)** δ 5.23 (bs, 1H), 3.16-3.11 (m, 2H), 2.58 (t, J=6.4Hz, 2H), 2.36 (t, J=7.2Hz, 2H), 1.99-1.95 (m, 3H), 1.64-1.57 (m, 6H), 1.37 (s, 9H); **^{13}C NMR (101 MHz, $CDCl_3$)** δ 156.29, 82.71, 79.08, 69.33, 47.36, 43.99, 38.86, 32.51, 32.41, 29.69, 29.44, 28.43, 28.40, 26.99, 13.29.

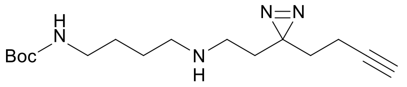

22

Compound **22** was prepared from **21** (500 mg, 1.34 mmol) following **P** Procedure 4. The product was obtained as a pale-yellow solid 91 mg (22%); **TLC** (5% MeOH/DCM): Rf=0.23; **^{1}H NMR (400 MHz, $CDCl_3$)** δ 4.81 (bs, 1H), 3.11 (m, 2H), 2.58 (m, 2H), 2.41 (t, J=8Hz, 2H), 2.04-1.99 (m, 3H), 1.71-1.63 (m, 4H), 1.53-1.49 (m, 4H), 1.44 (s, 9H); **^{13}C NMR (101 MHz, $CDCl_3$)** δ 156.21, 82.85, 69.38, 49.38, 44.11, 40.47, 32.70, 32.61, 29.88, 28.58, 27.94, 27.16, 27.05, 13.44.

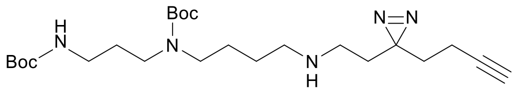

15

Compound **15** was prepared from **14** (570 mg, 1.08 mmol) following Procedure 4. The product was obtained as a pale-yellow solid 160 mg (32%); **TLC** (5% MeOH/DCM): Rf=0.23; **^{1}H NMR (400 MHz, $CDCl_3$)** δ 5.27 (bs, 1H), 3.16-2.98 (m, 6H), 2.58-2.53 (m, 2H), 2.39(bs, 2H), 1.96-1.92 (m, 3H), 1.67-1.54 (m, 6H), 1.47-1.40 (m, 4H), 1.37 (bs, 9H), 1.35 (bs, 9H); **^{13}C NMR (101 MHz, $CDCl_3$)** δ 156.03, 155.48, 136.79, 115.56, 82.60, 79.47, 77.36, 69.36, 49.12, 46.78, 46.28, 44.30, 43.71, 37.39, 32.35, 31.98, 28.61, 28.42, 28.21, 27.85, 13.25.

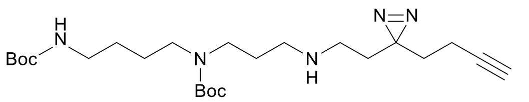

26

Compound **26** was prepared from **25** (500 mg, 0.95 mmol) following Procedure 4. The product was obtained as a pale-yellow solid 130 mg (29%); **TLC** (5% MeOH/DCM): Rf=0.19; **^{1}H NMR (400 MHz, $CDCl_3$)** δ 4.69 (bs, 1H), 3.18-3.00 (m, 6H), 2.55-2.48 (m, 2H), 2.40-2.32 (m, 2H), 1.95-1.90 (m, 3H), 1.67-1.54 (m, 6H), 1.46-1.37 (m, 4H), 1.35 (s, 9H), 1.34 (s, 9H); **^{13}C NMR (101 MHz, $CDCl_3$)** δ 156.14, 155.96,

155.45, 115.51, 82.58, 79.73, 78.94, 77.36, 69.31, 46.67, 46.23, 43.80, 40.08, 32.28, 29.61, 28.73, 28.55, 28.38, 28.16, 27.35, 25.71, 13.22.

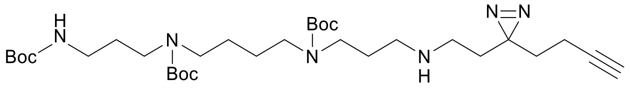

20

Compound **20** was prepared from **19** (1.08 g, 1.57 mmol) according to Procedure 2. The product was obtained as a pale-yellow solid 100 mg (10%); **TLC** (5% MeOH/DCM): Rf=0.12; **^{1}H NMR (400 MHz, $CDCl_3$)** δ 5.28 (bs, 1H), 3.25-2.85 (m, 16H), 1.89 (bs, 3H), 1.55 (bs, 4H), 1.34 (bs, 33H); **^{13}C NMR (101 MHz, $CDCl_3$)** δ 156.76, 156.00, 155.33, 129.36, 80.45, 79.43, 78.76, 77.36, 46.90, 46.67, 46.20, 44.14, 43.64, 42.93, 37.57, 37.23, 36.89, 29.55, 28.83, 28.38, 28.34, 28.29, 25.85, 25.30.

2.7. Procedure 5 – Boc deprotection and formation of hydrochloride salts

Compound **9**, **11**, **15**, **20**, **22** or **26** was cooled in an ice-water bath. Cold 4 M HCl in dioxane was added dropwise (1 mL for every 100 mg of sample), followed by a few drops of Milli-Q water. The reaction mixture was stirred at 0 °C for 1 h and then concentrated under reduced pressure. The residue was resuspended in 3 mL of MeOH and 1 mL of $CHCl_3$ and concentrated under reduced pressure. This washing procedure was repeated three times, reducing the volume of MeOH with each repetition, to yield the hydrochloride salt of the corresponding compound **1**, **2**, **3**, **4**, **5** or **6** as an off-white solid. The yield for each reaction was quantitative.

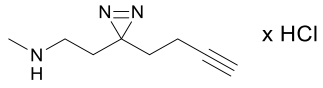

1

^{1}H NMR (400 MHz, D_2O) δ 3.01 (t, J=8Hz, 2H), 2.70 (s, 3H), 2.42-2.41 (m, 1H), 2.07 (m, 2H), 1.86 (t, J= 8Hz, 2H), 1.73 (t, J=7.2Hz, 2H); **^{13}C NMR (101 MHz, D_2O)** δ 83.96, 70.00, 43.81, 32.77, 30.39, 29.24, 26.39, 12.30. **HRMS** (m/z): $[M+H]^+$ calculated for $C_8H_{14}N_3$ 152.1182; found 152.1183

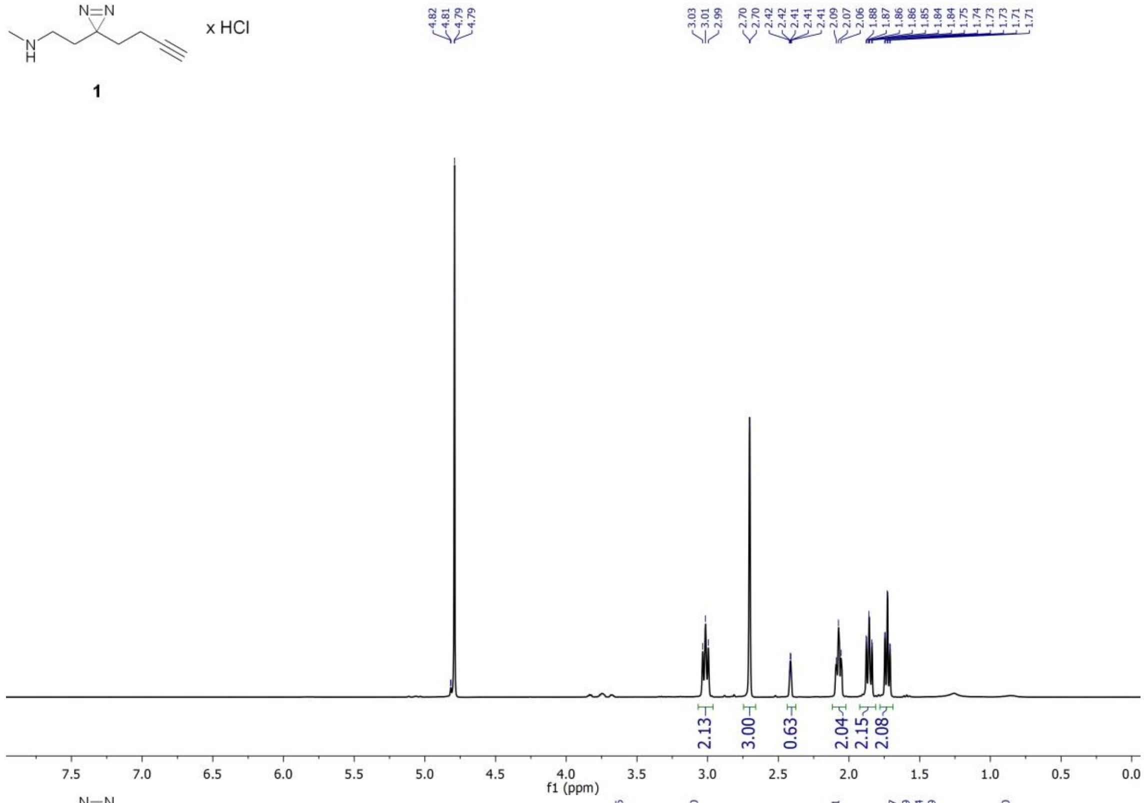

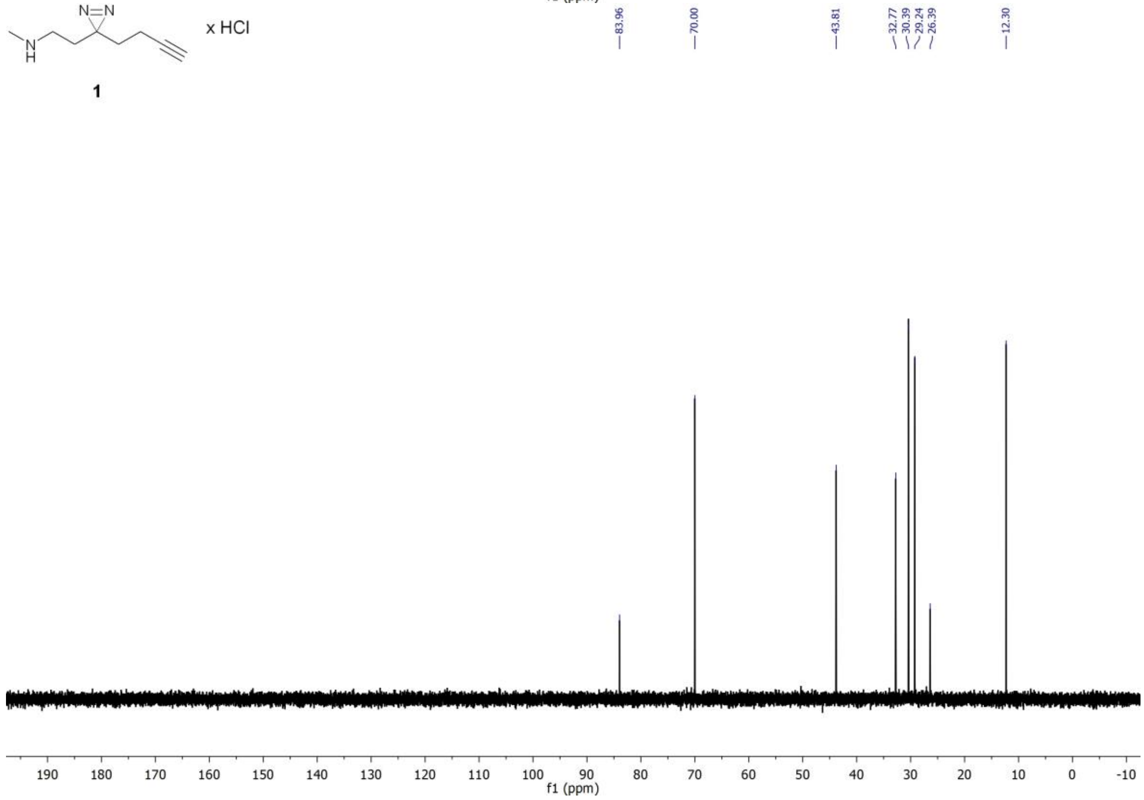

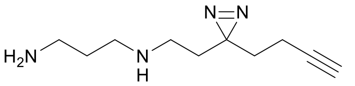

x 2 HCl

2

[1]**H NMR (400 MHz, D_2O)** δ 3.13-3.00 (m, 6H), 2.37 (bt, J= 2.8Hz, 1H), 2.08-2.00 (m, 4H), 1.83 (t, J=8Hz, 2H), 1.70 (t, J=7.2Hz, 2H); [13]**C NMR (101 MHz, D_2O)** δ 83.89, 69.96, 44.50, 42.49, 36.36, 30.34, 29.31, 26.35, 23.54, 12.28. **HRMS** (m/z): $[M+H]^+$ calculated for $C_{10}H_{19}N_4$ 195.1604; found 195.1606

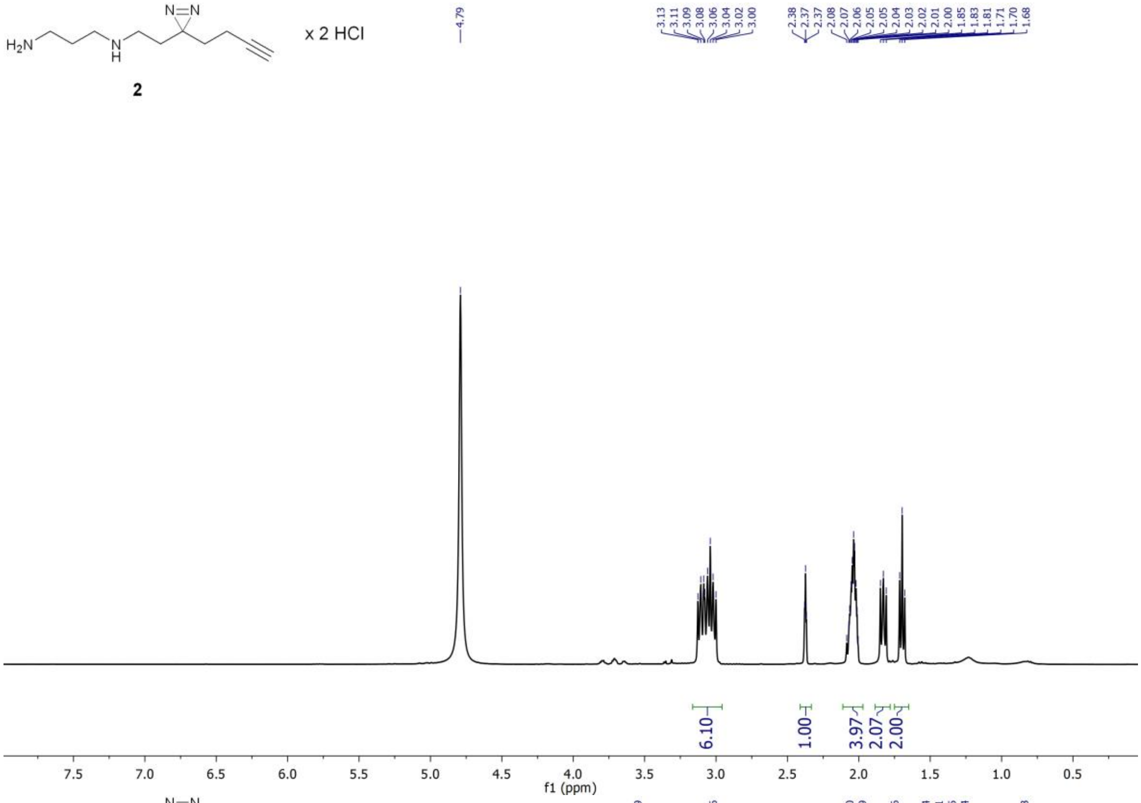

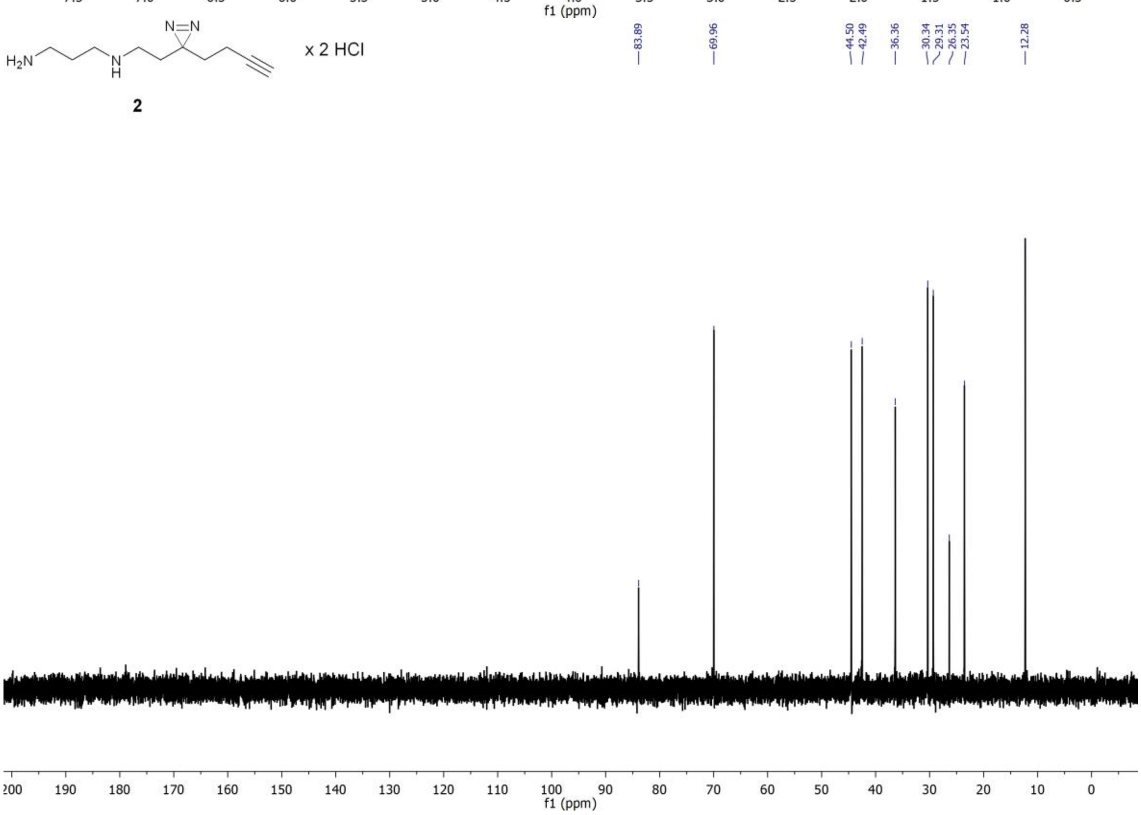

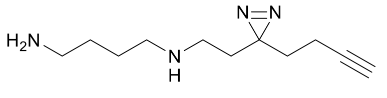

x 2HCl

3

^{1}H NMR (400 MHz, D_2O) δ 3.07-2.99 (m, 6H), 2.39 (t, J=2.4Hz, 2H), 2.05 (td,J_1=2.4Hz, J_2=7.2Hz, 2H), 1.86-1.81 (m, 2H), 1.75-1.69 (m, 6H); **^{13}C NMR (101 MHz, D_2O)** δ 83.91, 69.96, 46.87, 42.31, 38.65, 30.38, 29.31, 26.39, 23.78, 22.56, 12.29. **HRMS** (m/z): $[M+H]^+$ calculated for $C_{11}H_{21}N_4$ 209.1761; found 209.1762

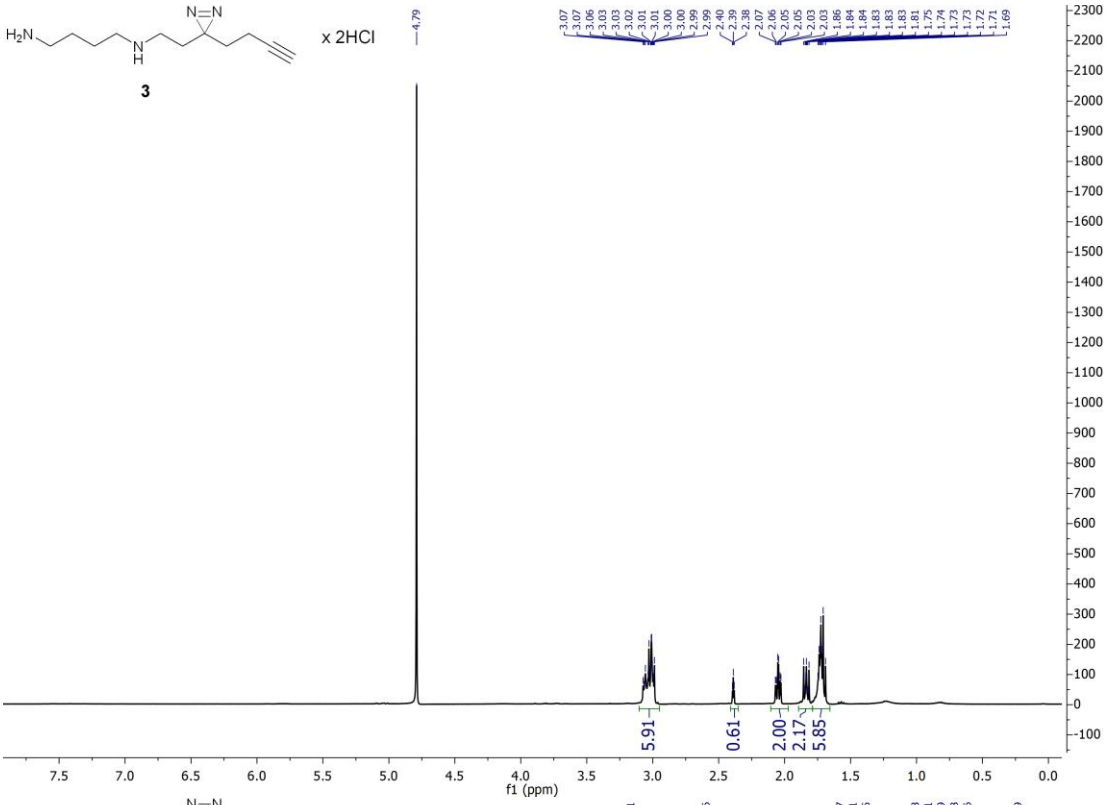

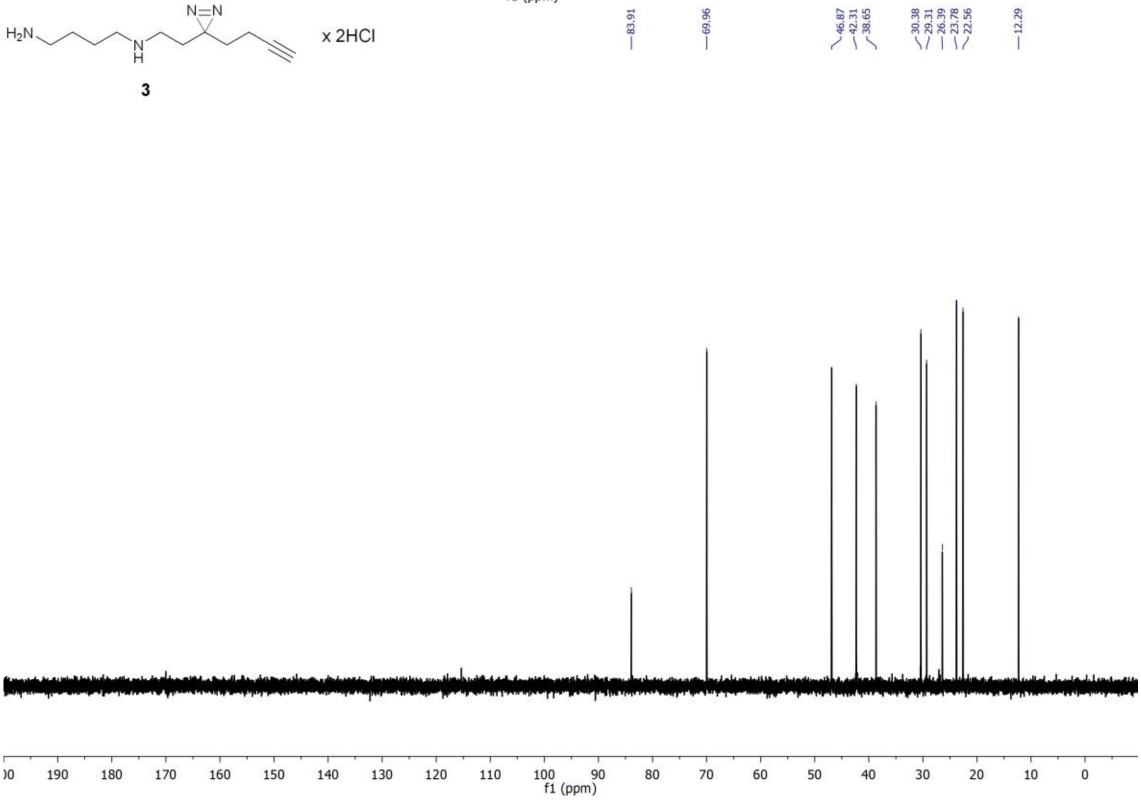

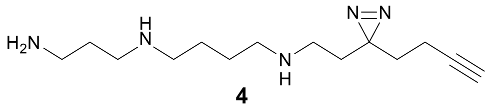

x 3HCl

4

^{1}H NMR (400 MHz, D_2O) δ 3.17-3.00 (m, 10H), 2.41-2.40 (m, 1H), 2.13-2.04 (m, 4H), 1.87-1.83 (m, 2H), 1.79-1.71 (m, 6H); **^{13}C NMR (101 MHz, D_2O)** δ 83.95, 69.98, 46.89, 46.82, 44.44, 42.33, 36.44, 30.39, 29.32, 26.41, 23.65, 22.66, 22.59, 12.31. **HRMS** (m/z): $[M+H]^+$ calculated for $C_{14}H_{28}N_5$ 266.2339; found 266.2343

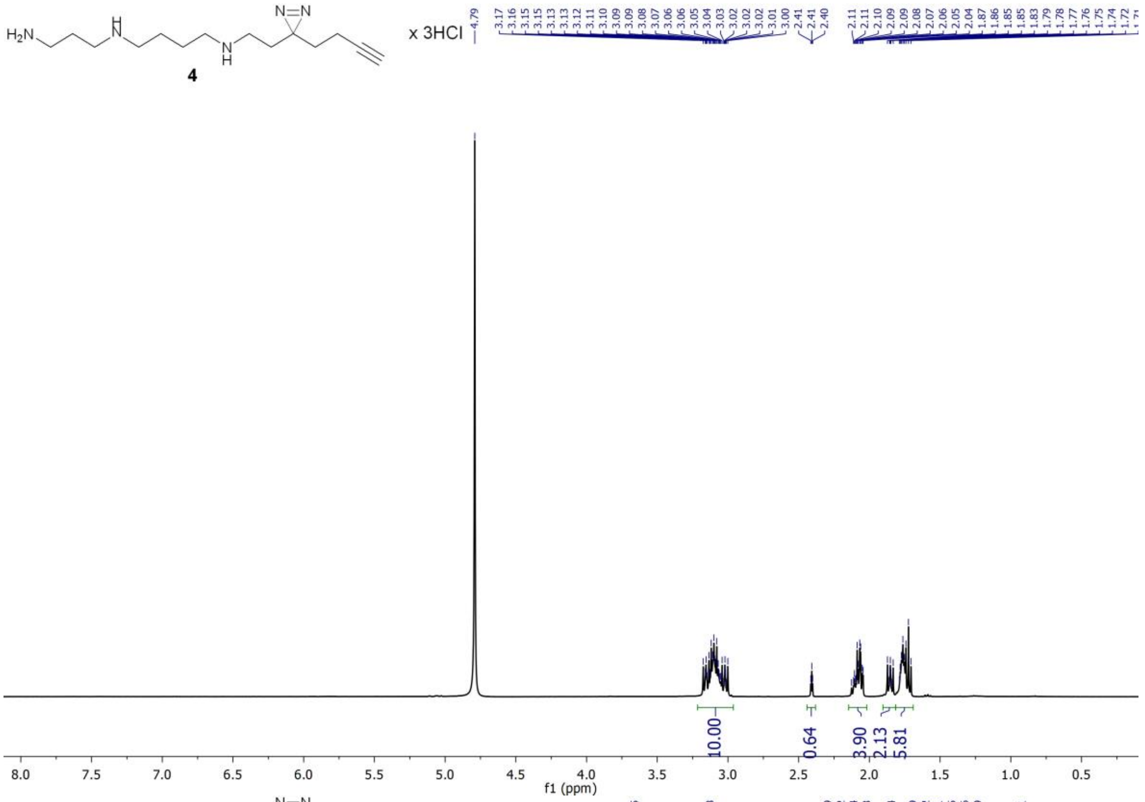

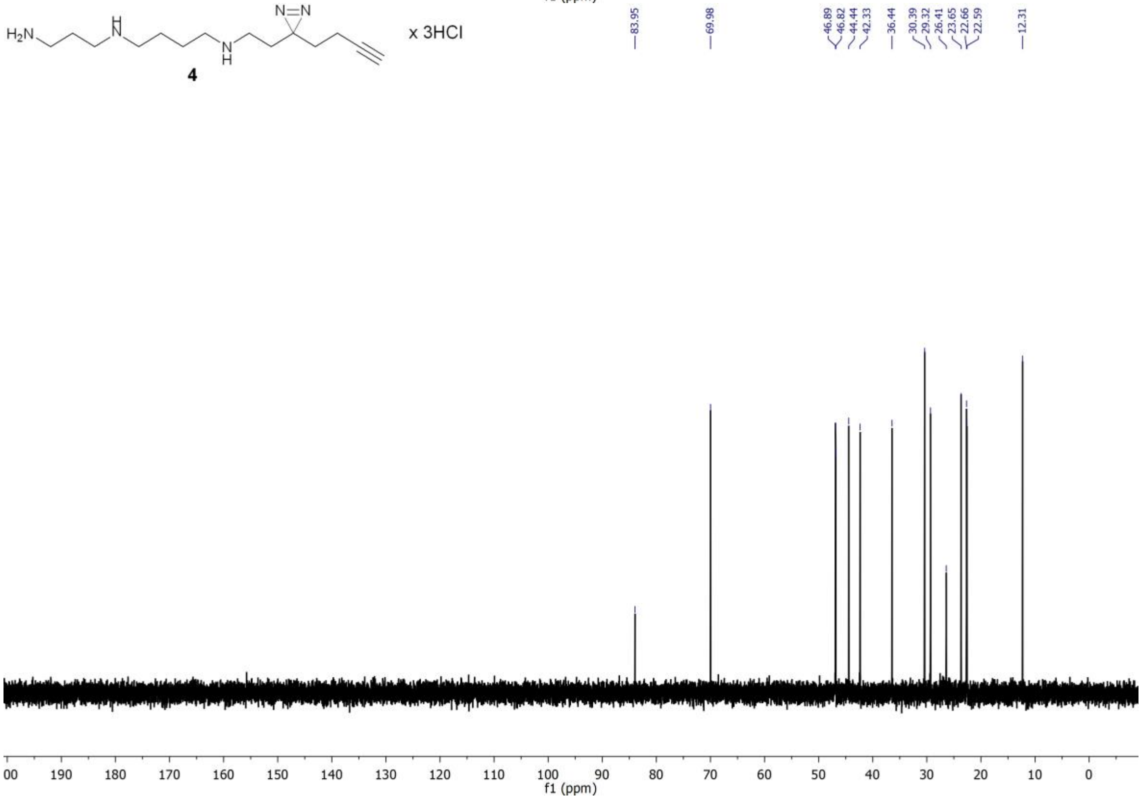

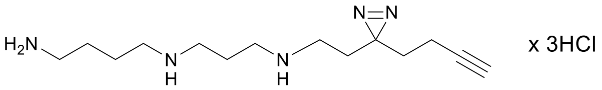

5

^{1}H NMR (400 MHz, D_2O) δ 3.16-3.01 (m, 10H), 2.40 (bs, 1H), 2.12-2.04 (m, 4H), 1.87-1.83 (m, 2H), 1.79-1.70 (m, 6H); **^{13}C NMR (101 MHz, D_2O)** δ 83.95, 70.01, 46.98, 44.46, 44.32, 42.54, 38.69, 30.38, 29.34, 26.39, 23.80, 22.65, 22.51, 12.31. **HRMS** (m/z): $[M+H]^+$ calculated for $C_{14}H_{28}N_5$ 266.2339; found 266.2342

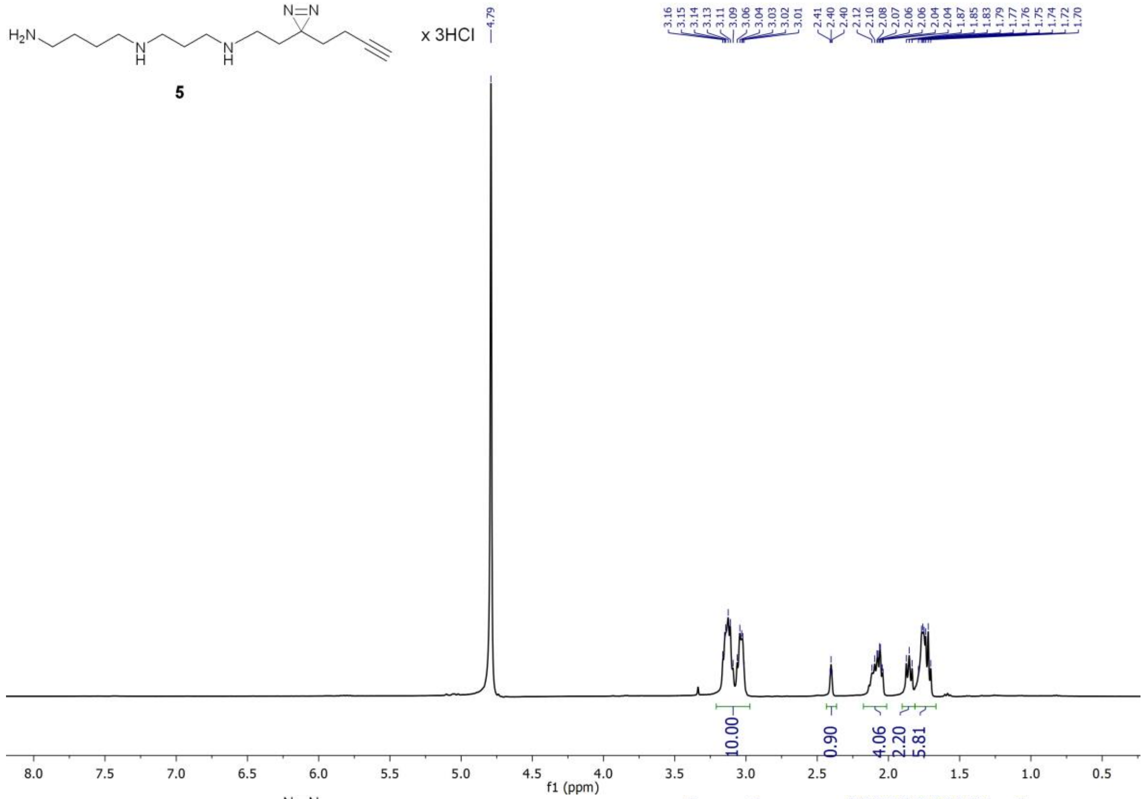

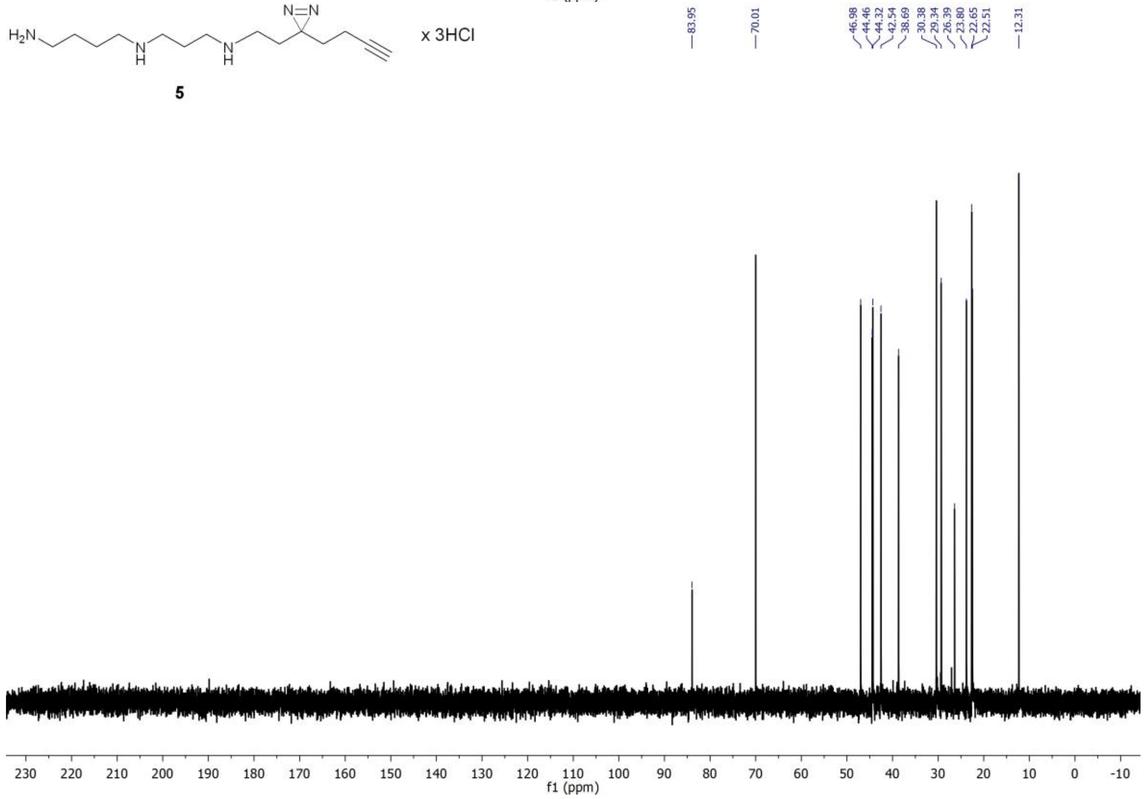

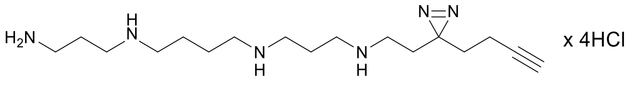

6

^{1}H NMR (400 MHz, D_2O) δ 3.13-2.94 (m, 14H), 2.35 (bs, 1H), 2.08-2.00 (m, 6H), 1.83-1.78 (m, 2H), 1.75-1.65 (m, 6H); **^{13}C NMR (101 MHz, D_2O)** δ 83.85, 69.93, 46.86, 46.78, 44.40, 44.28, 42.50, 42.30, 36.38, 30.32, 29.30, 26.33, 23.61, 22.62, 22.56, 22.48, 12.27. **HRMS** (m/z): $[M+H]^+$ calculated for $C_{17}H_{35}N_6$ 323.2918; found 323.2920

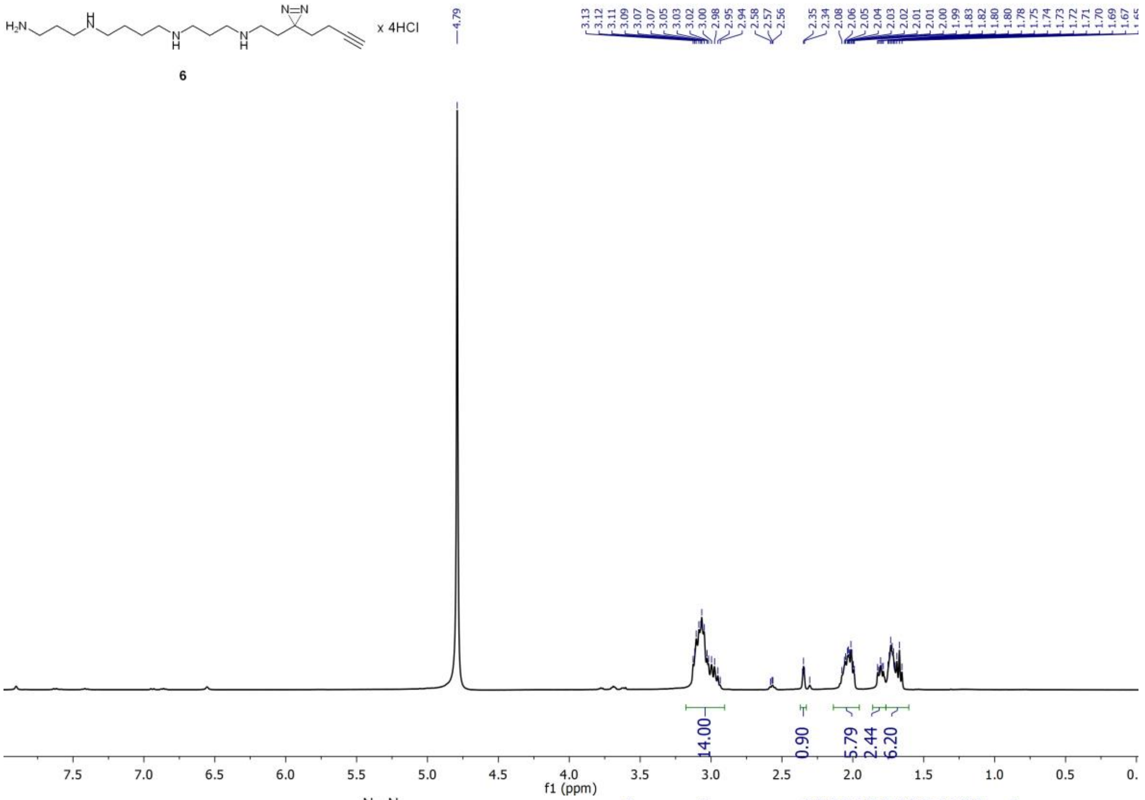

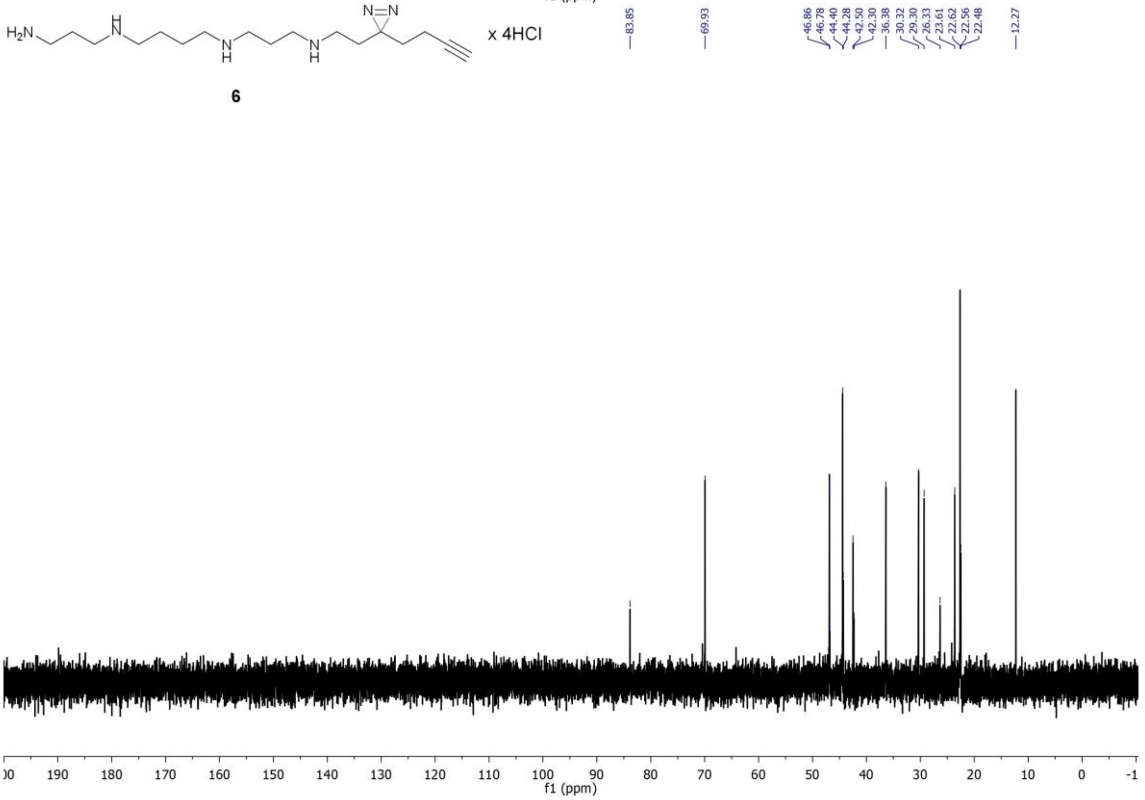

3. Figures S1-12

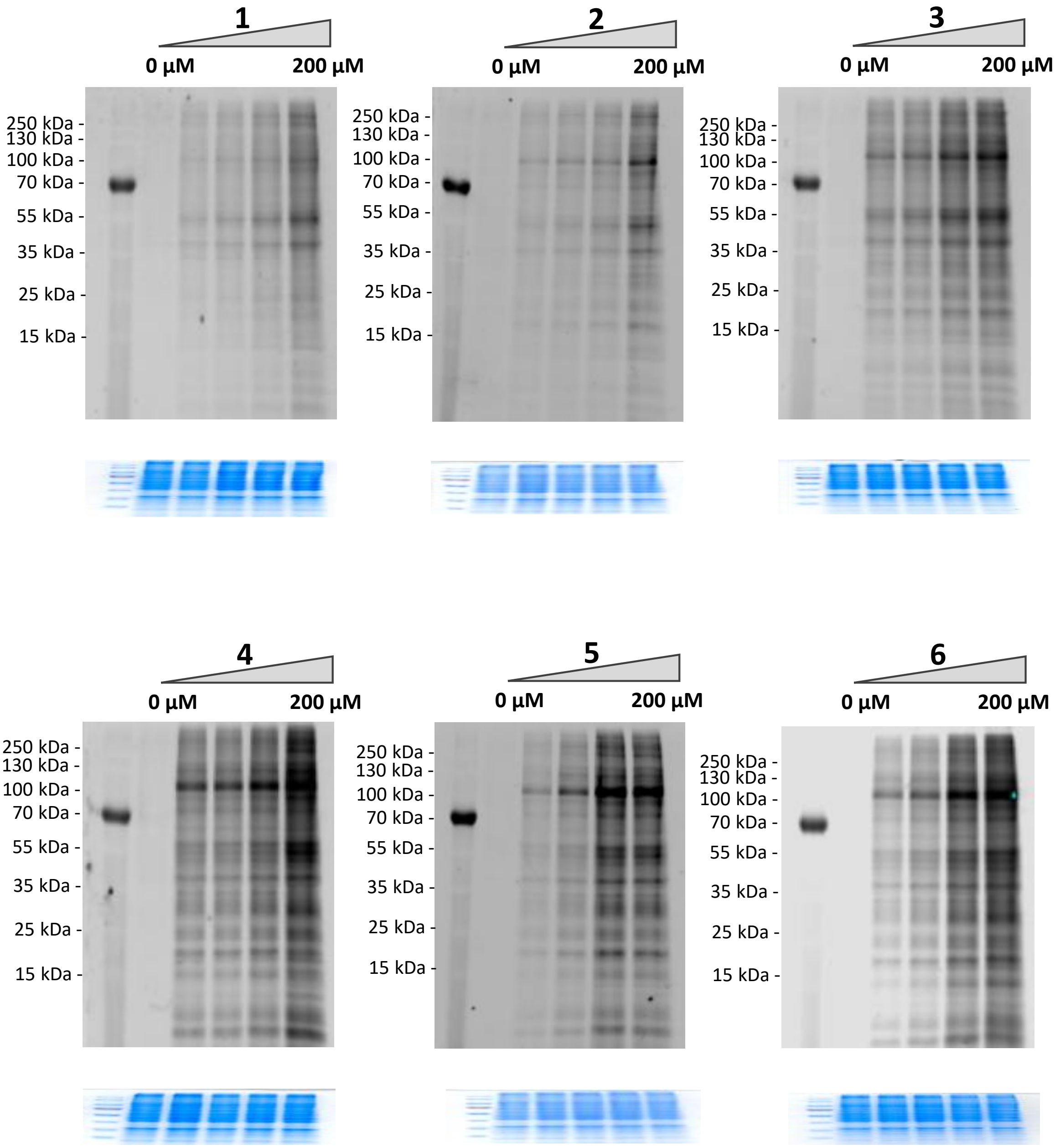

Figure S1. Probe-dose-dependent photoaffinity labelling with polyamine analogs in cell lysates. HeLa cell lysates were incubated with probes **1**–**6** at varying concentrations (0, 25, 50, 100, or 200 µM) for 15 min at 4 °C and subjected to UV irradiation at 365 nm. Following photoaffinity labeling, probe-protein conjugates were ligated to 5-TAMRA-azide dye, separated by SDS-PAGE, and visualized using in-gel fluorescence imaging (grey-scale images). Total protein was stained with Coomassie Brilliant Blue (CBB, blue images). To conserve space, the CBB-stained gel images were horizontally compressed, while preserving accurate representation of relative protein loading across the lanes.

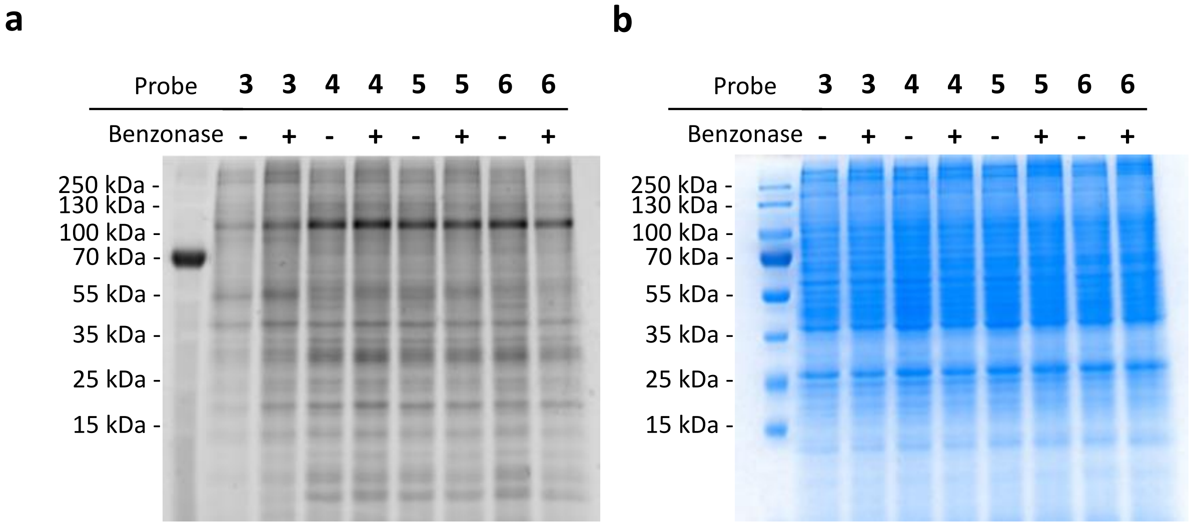

Figure S2. Photoaffinity labelling with polyamine analogs following pre-treatment of cell lysates with the broad-specificity nuclease, Benzonase. HeLa cell lysates were pre-incubated with 30 units of Benzonase (**+**) or with Benzonase dilution buffer (**-**) for 30 min at 25 °C, followed by incubation with 50 µM probes **3**–**6** for 15 min at 4 °C, and UV irradiation at 365 nm. Probe-protein conjugates were ligated to 5-TAMRA-azide dye, separated by SDS-PAGE, and visualized using in-gel fluorescence imaging (**a**). Total protein was stained with Coomassie Brilliant Blue (**b**).

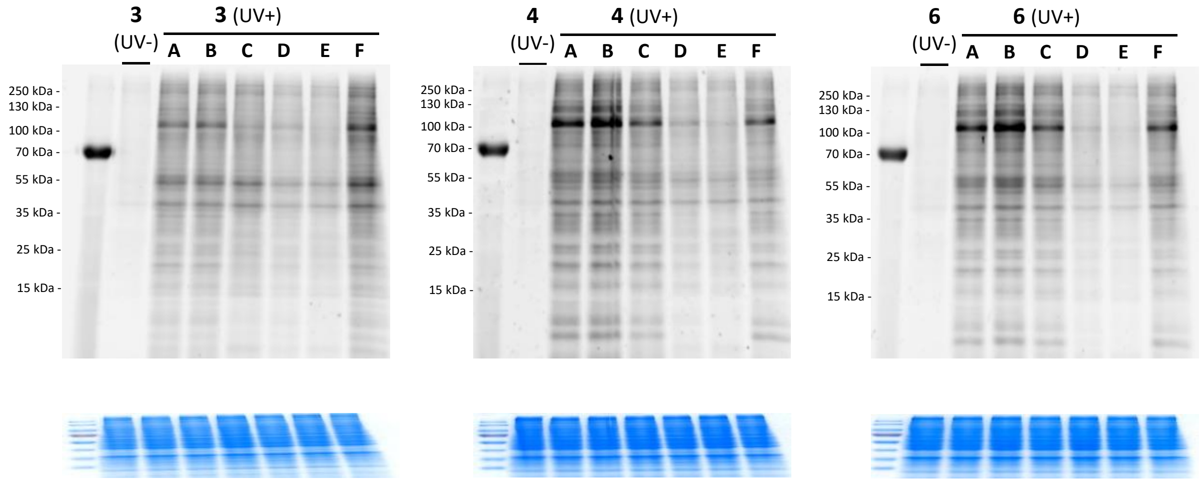

Figure S3. Competitive photoaffinity labelling with polyamine analogs in cell lysates. HeLa cell lysates were pre-incubated (or not - **A**) with 10 mM monoamine (dimethylamine - **B**), diamines (putrescine – **C** and synthetic 1,8-diaminooctane - **F**), or higher polyamines (spermidine - **D**, spermine - **E**) for 15 min at 4 °C, followed by incubation with 50 µM probes **3**, **4**, or **6** for 15 min 4 °C. Samples were then UV irradiated at 365 nm, while control samples were not exposed to UV light. Probe-protein conjugates were ligated to 5-TAMRA-azide dye, separated by SDS-PAGE, and visualized using in-gel fluorescence imaging (grey-scale images). Total protein was stained with Coomassie Brilliant Blue (CBB, blue images). To conserve space, the CBB-stained gel images were horizontally compressed, while preserving accurate representation of relative protein loading across the lanes.

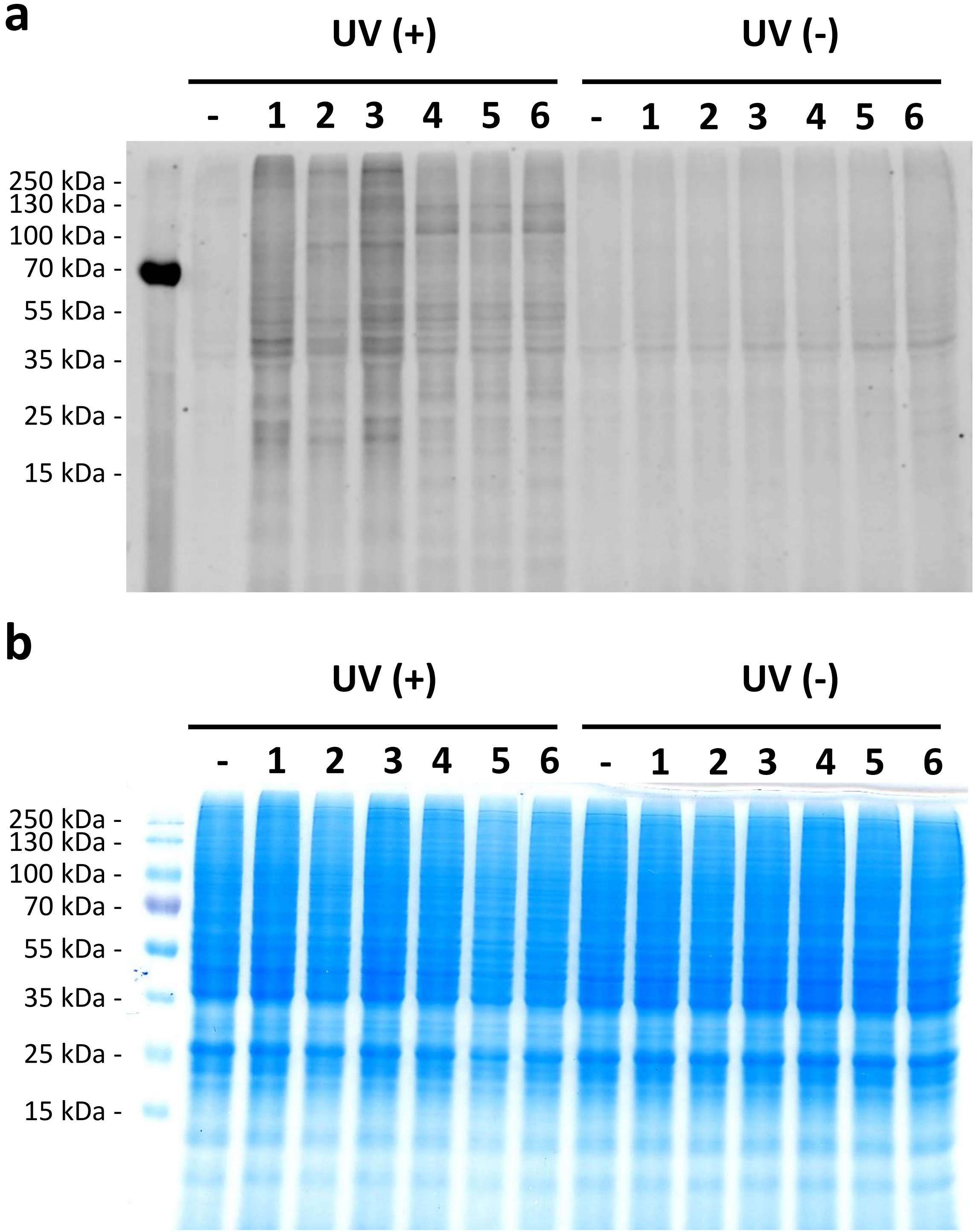

Figure S4. The profiles of protein interactors of polyamine analogs in live cells. HeLa cells in culture were incubated with vehicle (H_2O), compound **1** (240 µM), **2** (30 µM), **3** (30 µM), **4** (80 µM), **5** (80 µM), or **6** (80 µM) for 2 h and either UV irradiated or not at 365 nm. Probe-protein conjugates were ligated to 5-TAMRA-azide dye, separated by SDS-PAGE, and visualized using in-gel fluorescence imaging (**a**). Total protein was stained with Coomassie Brilliant Blue (**b**).

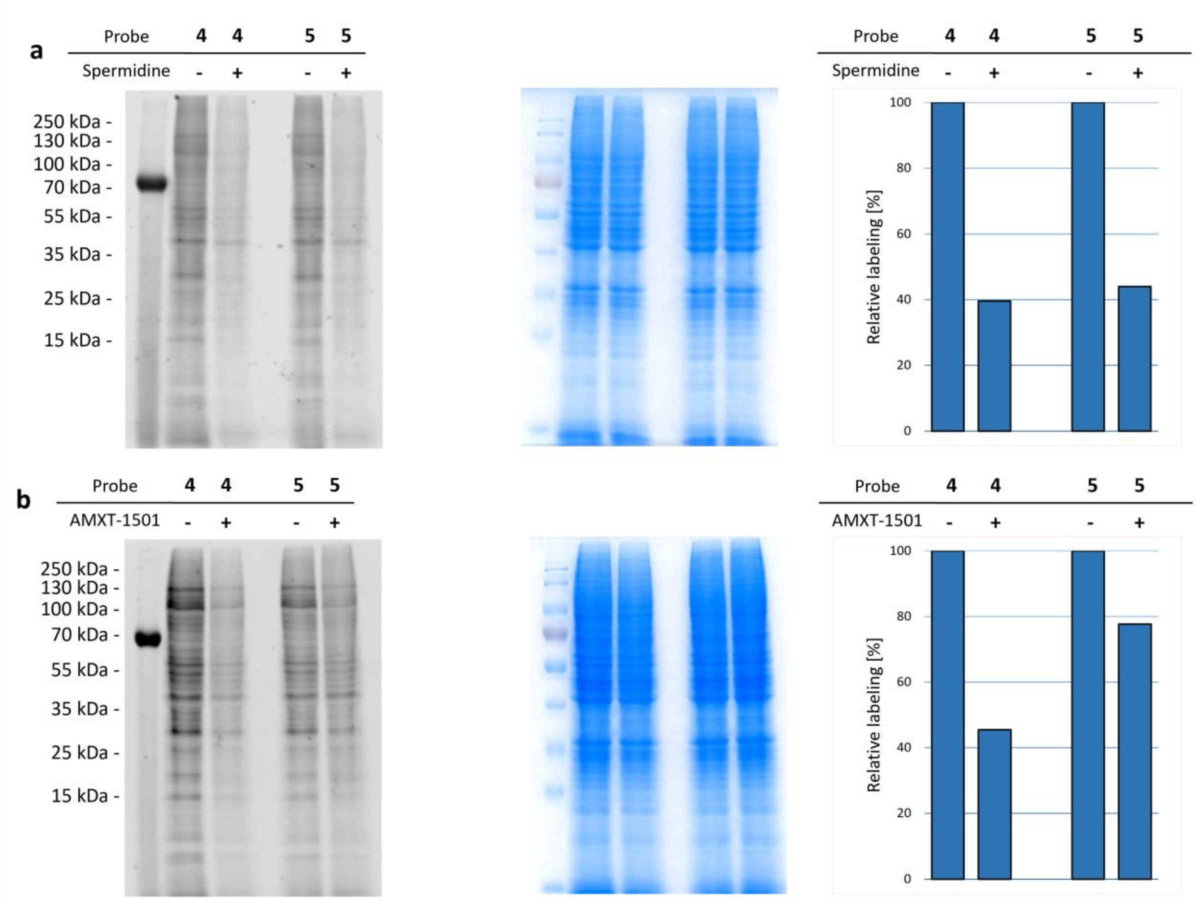

Figure S5. Photoaffinity labelling with polyamine analogs **4** and **5**. (a) HeLa cells were washed with Phosphate-buffered saline (PBS) and incubated with 2.5 mM spermidine in FBS-free DMEM supplemented with 1% P/S for 2h. Then compound **4** or **5** (25 µM) was added and the cells were incubated 1h. (b) HeLa cells were treated with 2.0 µM AMXT-1501 overnight. Then cells were washed with Phosphate-buffered saline (PBS) and compound **4** or **5** (75 µM) and AMXT-1501 (2 µM) were added in FBS-free DMEM supplemented with 1% P/S for 1h.

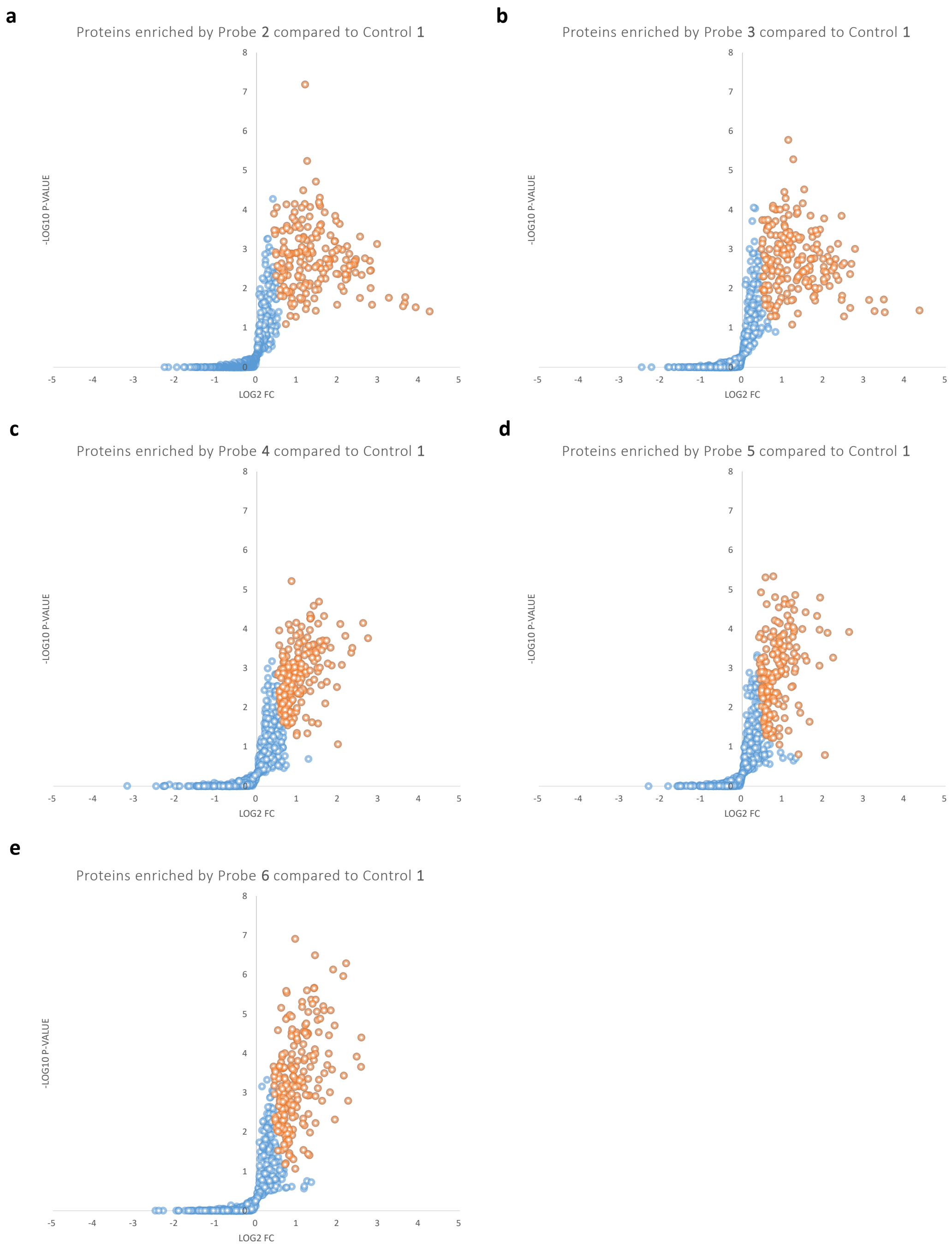

Figure S6. Profiling polyamine-binding proteins in HeLa cells. HeLa cells in culture were incubated with compound **1** (240 µM), **2** (25 µM), **3** (30 µM), **4** (75 µM), **5** (60 µM), or **6** (60 µM) for 2 hours, followed by UV irradiation at 365 nm. Probe-protein conjugates were ligated to azido-PEG3-biotin, enriched on Neutravidin beads, and digested on-bead. Tryptic peptides were desalted and TMT-labeled prior to analysis by liquid chromatography-tandem mass spectrometry (LC-MS/MS) for protein identification and quantification. Volcano plots depict proteins enriched by diamine probes **2**–**3** and higher polyamine probes **4**–**6** compared to the monoamine control compound **1**. Orange dots indicate polyamine probe-binding proteins identified based on statistical tests (one-sided Student's t-test, permutation-based FDR = 0.05, S0 = 0.5, n = 3). Log2 fold change (Log2FC, x-axes) and significance (-Log10 p-value, y-axes) of protein enrichment upon treatment with probes **2** (**a**), **3** (**b**), **4** (**c**), **5** (**d**), and **6** (**e**) versus compound **1** are presented. The numerical values used to generate the volcano plots are provided in Table S1.

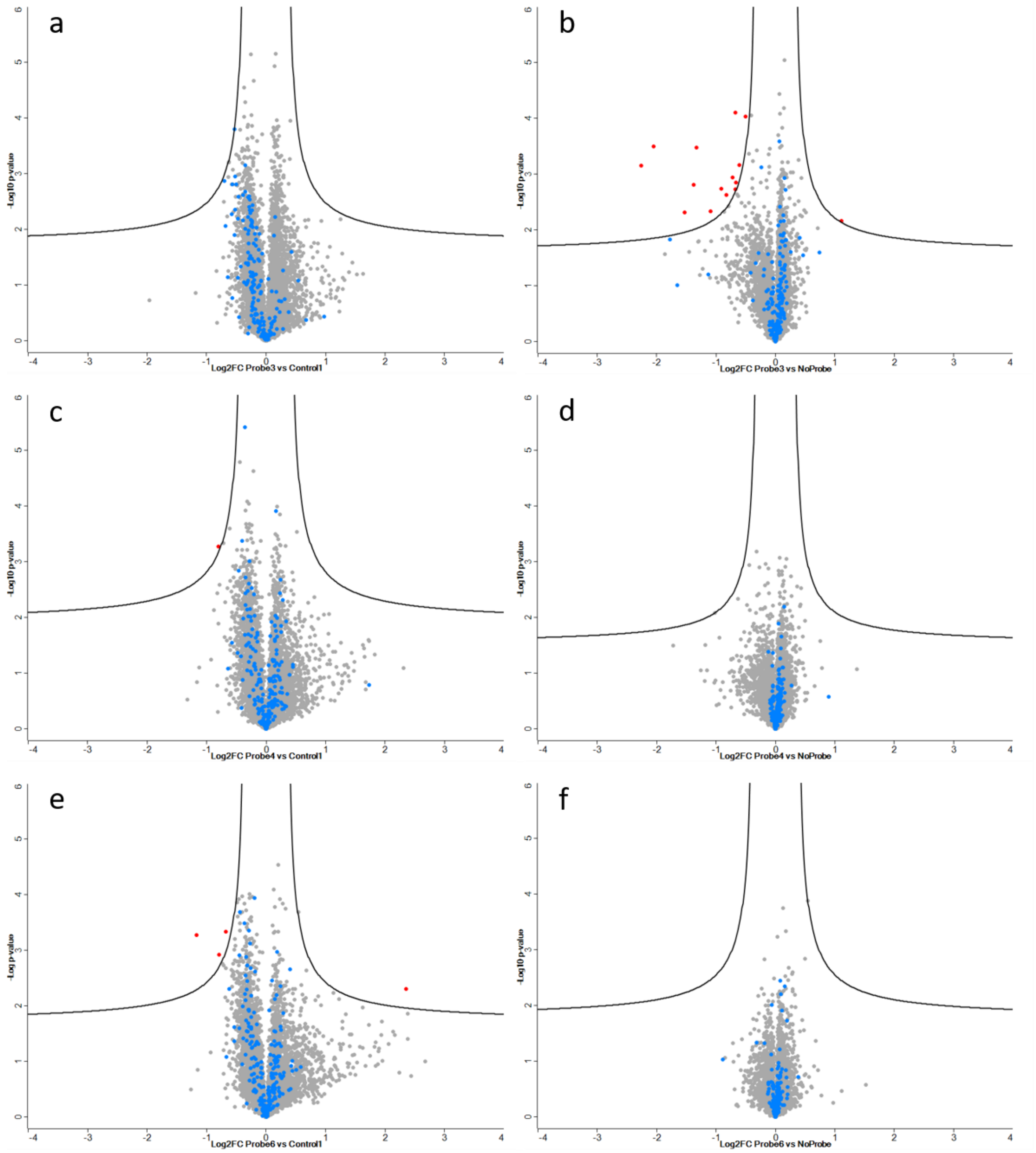

Figure S7. Effect of polyamine analog treatment on total protein levels in HeLa cells. Cells were incubated for 2 h with compound **1** (240 µM), **3** (30 µM), **4** (75 µM), or **6** (60 µM), and processed as for protein binder identification, omitting the bioorthogonal reaction and enrichment steps. Proteins were digested with trypsin, desalted, TMT-labeled, and analyzed by LC-MS/MS for protein identification and quantification. Volcano plots show protein level differences induced by putrescine analog **3**, spermidine analog **4**, and spermine analog **6**, compared to either the monoamine control compound **1** (panels a, c, e) or vehicle control (H_2O; panels b, d, f). Red dots indicate proteins significantly regulated based on on statistical tests (two-sided Student's t-test, permutation-based FDR = 0.01, S0 = 0.1, n = 3), blue dots indicate proteins identified as binders of specific polyamine probes in this study. The numerical values used to generate the volcano plots are provided in Table S2.

a

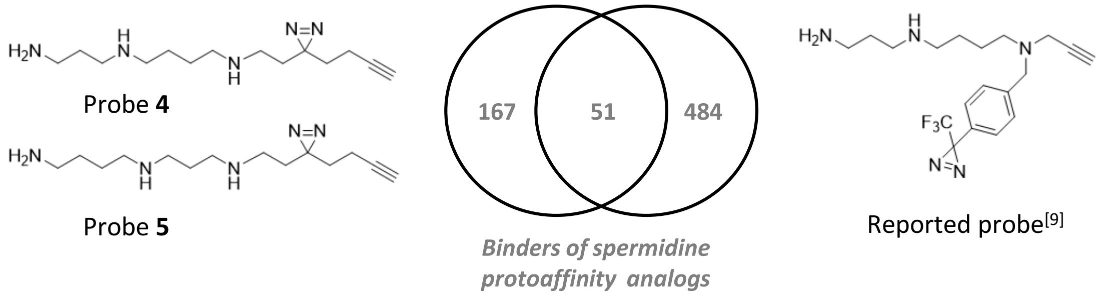

b

1	CAND1	18	KPNB1	35	RPLP0;RPLP0P6
2	CHD4	19	LARS	36	SF3B2
3	COPB2	20	LMNB1	37	SMU1
4	DEK	21	MCM2	38	SNRNP200
5	EFTUD2	22	MDC1	39	SPTBN1
6	EIF3C;EIF3CL	23	MYBBP1A	40	SRSF1
7	EIF3D	24	NCL	41	SRSF6
8	HNRNPC;HNRNPCL2	25	NOC2L	42	TCOF1
9	HNRNPU	26	NOP2	43	THRAP3
10	HNRNPUL2	27	NOP56	44	TNPO1
11	HP1BP3	28	NPEPPS	45	TUBA1B;TUBA1A;TUBA3E
12	HSP90AA1	29	NPM1	46	TUBB
13	HSP90AB1	30	PA2G4	47	TUBB2B;TUBB2A
14	IARS2	31	PDIA4	48	TUBB3
15	IPO5	32	RPL18A	49	TUBB4B;TUBB4A
16	IPO7	33	RPL4	50	TUBB6
17	KARS	34	RPL5	51	VCP

Figure S8. Putative binders of spermidine photoaffinity probes. **a.** Venn diagram representing the overlap between the protein binders of compounds **4** and/or **5** in HeLa cells and the protein binders of recently reported spermidine-like probe in HEK 293 cells.[13] A total of 218 protein groups were identified as binders of probes **4** and/or **5** based on enrichments upon treatment with these probes compared to the monoamine control compound **1**, using statistical testing (one-sided Student's t-tests, permutation-based FDR = 0.05, S0 = 0.5, n = 3). Candidate binders of the reported probe were selected based on the Log2 fold change (Log2FC) values. As neither intensity values for individual samples nor statistical significance values were reported by the authors, 535 candidate binders meeting the criterion Log2FC Probe/DMSO >0.5 were selected for this analysis. **b.** Gene names of the 51 protein binders shared between both studies.

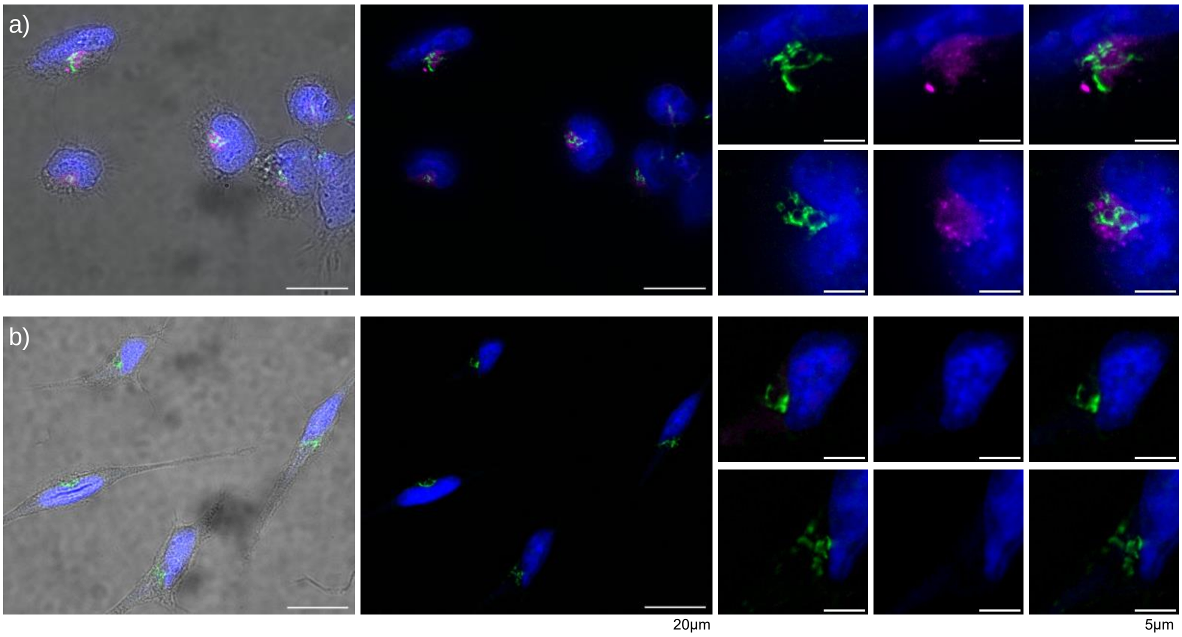

Figure S9. The subcellular localization of putrescine analog **3** conjugates (pink) and Golgi apparatus marker GM-130 (green) in HeLa cells. Cells were exposed to the photoaffinity probe **3** (30 µM) for 2h, followed by UV irradiation at 365 nm (**a**) or no irradiation (**b**). The cells were fixed with methanol, the probe conjugates ligated to 5-TAMRA-azide, and the immunostaining for GM-130 performed. The nucleus was stained with DAPI (blue). Fluorescence microscopy was used for visualization, with signals processed through background subtraction and deconvolution. Scale bars: 20 µm for wider fields and 5 µm for single cells.

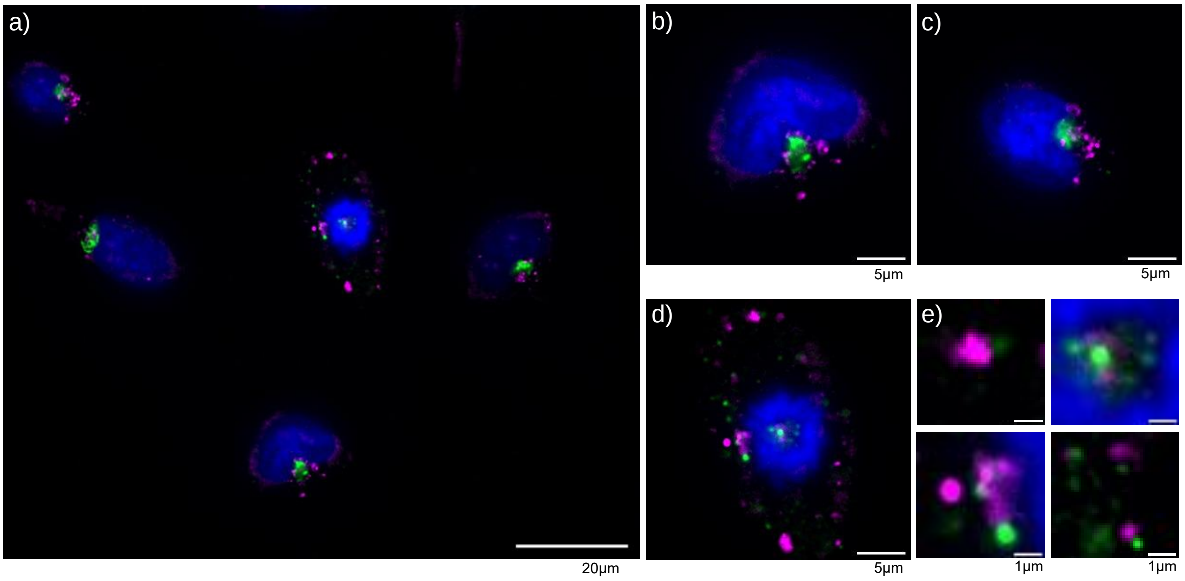

Figure S10. The subcellular localization of putrescine analog **3** conjugates (pink) and Golgi apparatus marker GM-130 (green) in HeLa cells during interphase (**b**-**c**) and mitosis (**d**-**e**). Cells were exposed to probe **3** (30 µM) for 2h, followed by UV irradiation at 365 nm. The cells were fixed with methanol, the probe conjugates ligated to 5-TAMRA-azide, and the immunostaining for GM-130 performed. The nucleus was stained with DAPI (blue). Fluorescence microscopy was used for visualization, with signals processed through background subtraction and deconvolution. Scale bars: 20 µm for wider fields (**a**), 5 µm for single cells (**b**-**d**), and 1 µm for cell the subcellular regions (**e**).

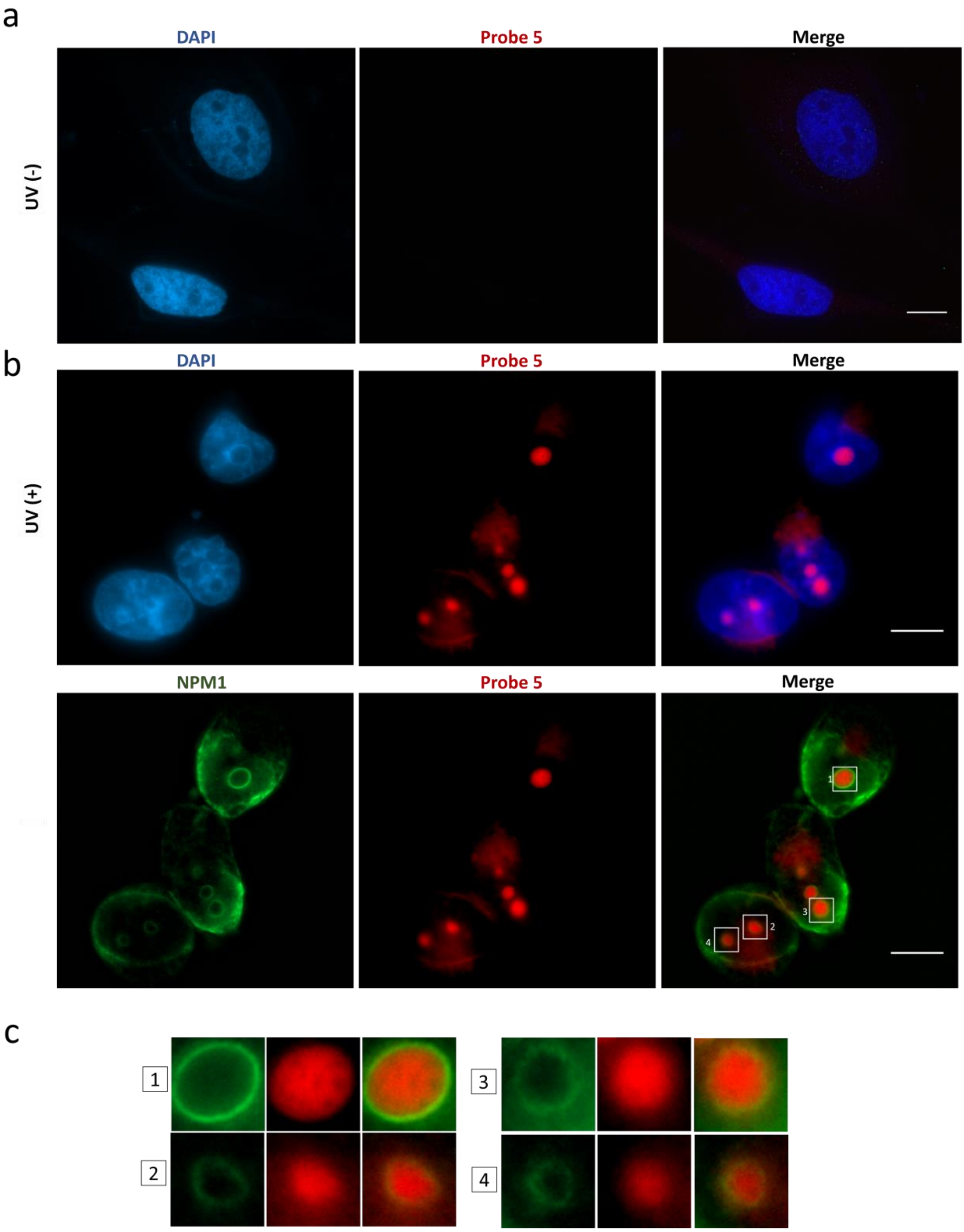

Figure S11.The subcellular localization of spermidine analog **5** conjugates (red) and nucleolar rim marker NPM1 (green) in HeLa cells. Cells were exposed to probe **5** (60 µM, 2 h) followed by either no irradiation (**a**) or UV irradiation at 365 nm (**b-c**). Cells were methanol-fixed; probe conjugates were ligated to 5-TAMRA-azide, and NPM1 was detected by immunostaining. Nuclei were counterstained with DAPI (blue). Images were acquired by fluorescent microscopy and processed by background subtraction. (**c**) Magnified subnuclear regions (indicated in panel b). Scale bar: 10 µm.

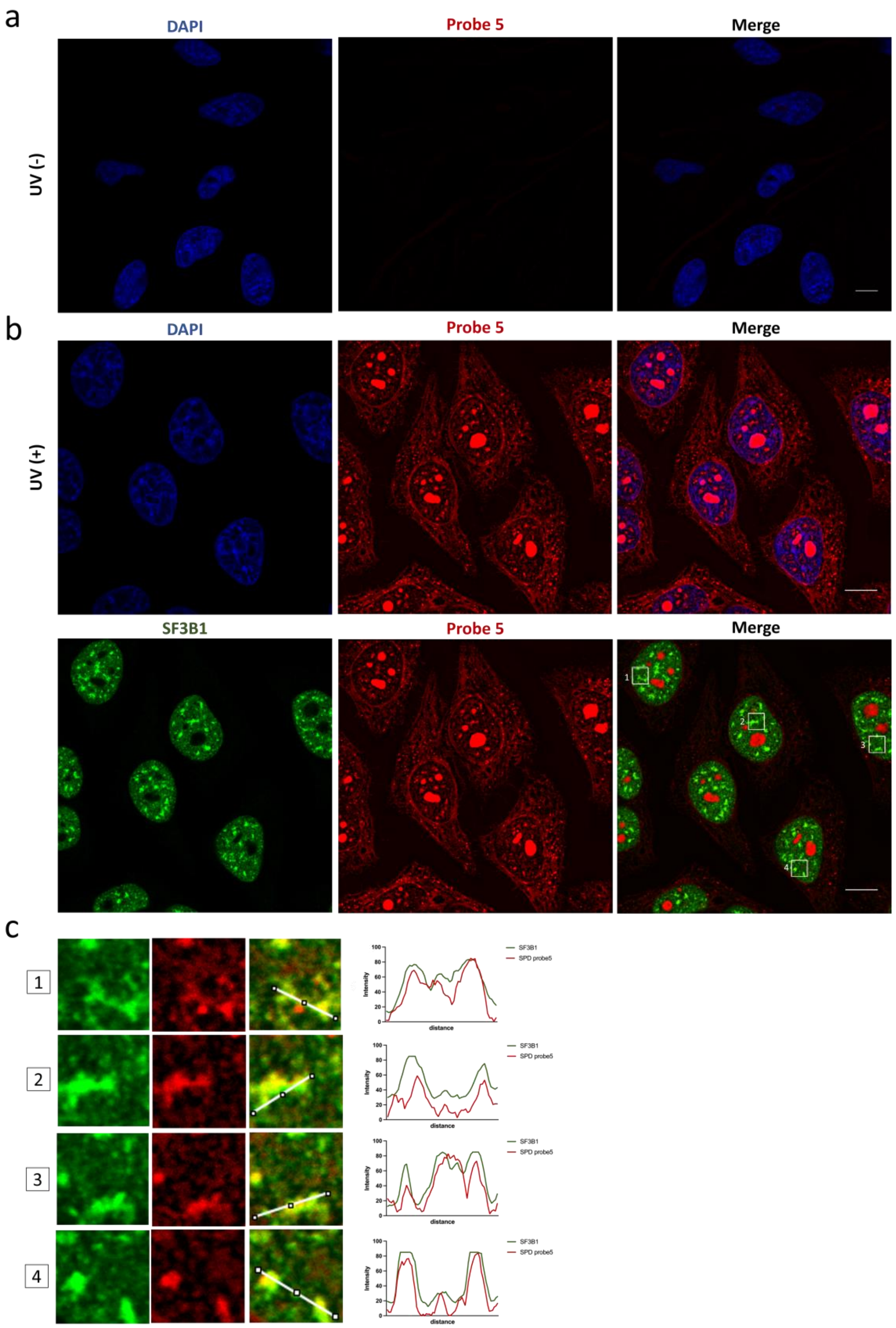

Figure S12.The subcellular localization of spermidine analog **5** conjugates (red) and nuclear speckles marker SF3B1 (green) in HeLa cells. Cells were exposed to probe **5** (60 µM, 2 h) followed by either no irradiation (**a**) or UV irradiation at 365 nm (**b**-**c**). Cells were methanol-fixed; probe conjugates were ligated to 5-TAMRA-azide, and SF3B1 was detected by immunostaining. Nuclei were counterstained with DAPI (blue). Images were acquired by confocal microscopy and processed by background subtraction and deconvolution. (**c**) Magnified subnuclear regions (left; indicated in b) with superimposed intensity profiles of SF3B1 and probe 5 (right), obtained in ImageJ/Fiji[2] along the white lines crossing selected nuclear speckles. Scale bar: 10 µm.

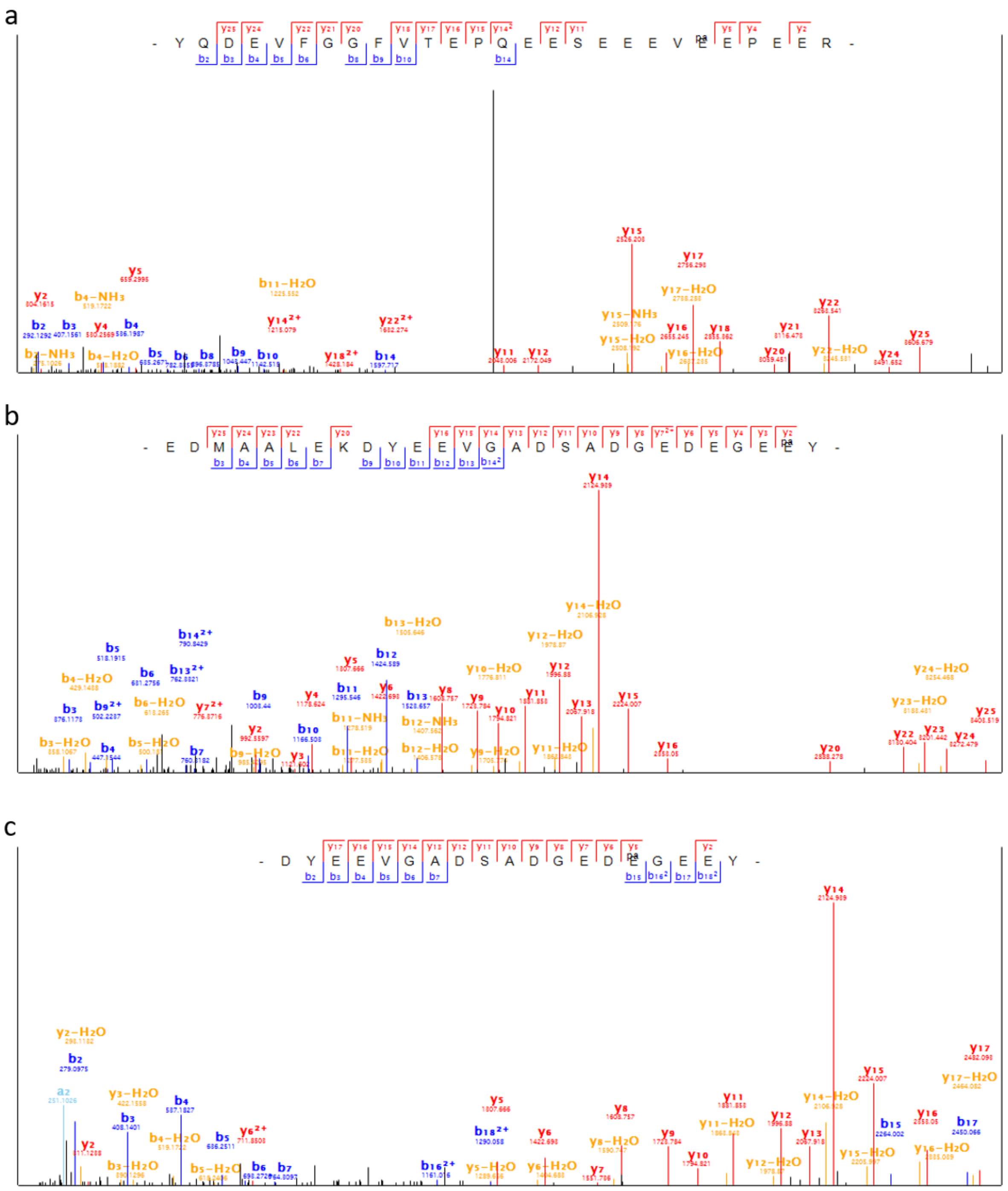

Figure S13. MS/MS spectra of selected probe **5**-modified peptides detected after photoaffinity labeling with probe **5** in HeLa cells (2h incubation at 60 μM, then 10 min irradiation at 365 nm) followed by bioorthogonal ligation to azide-PEG$_3$-biotin, tryptic digestion, and peptide-level affinity enrichment. **a.** Peptide YQDEVFGGFVTEPQEESEEEVEEPEER (residues 133–159) mapping to the N-terminal portion of IDR1 in G3BP1. **b-c.** Peptides EDMAALEKDYEEVGADSADGEDEGEEY (residues 423–449) and DYEEVGADSADGEDEGEEY (residues 431–449) mapping to the C-terminus of TUBA1C. LC-MS/MS data were analyzed and pictures generated in MaxQuant v. 1.6.17.0.[5]

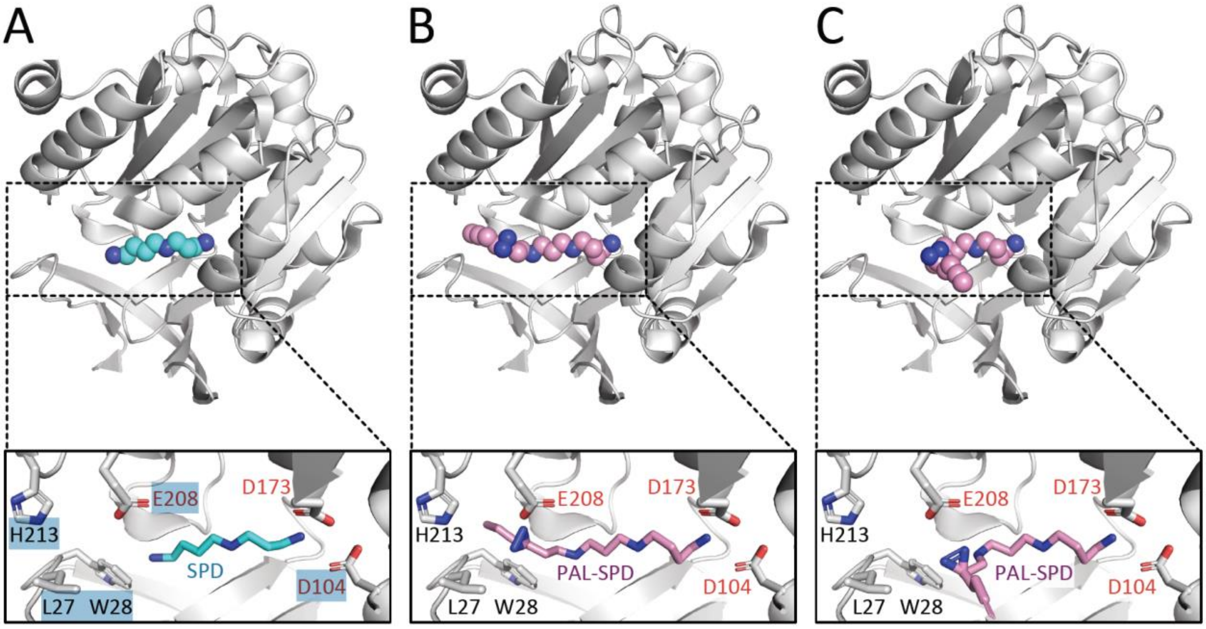

Figure S14. Modeling of spermidine analog 5 binding with spermidine synthase (SRM) based on spermidine co-crystal structure with SRM (PDB 2O07).[14] **a.** Native spermidine bound to SRM in a product-like conformation bridging the amine-acceptor and amine-donor binding sites. **b-c.** Two representative poses of Probe **5** occupying a spermidine-like binding mode; in both poses the alkyl diazirine is positioned within reach of E208, while the propargyl handle projects toward solvent, outside the polyamine-binding pocket. Modeling was performes and pictures generated in PyMOL v. 2.6.2.

a

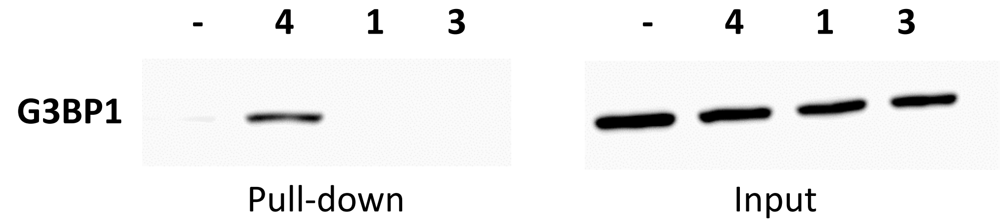

b

Gene name	Log2FC **2** vs **1**	T-test q-value 2 vs 1	Enriched in 2 vs 1	Log2FC **3** vs **1**	T-test q-value 2 vs 1	Enriched in 3 vs 1	Log2FC **4** vs **1**	T-test q-value 4 vs 1	Enriched in 4 vs 1	Log2FC **5** vs **1**	T-test q-value 5 vs 1	Enriched in 5 vs 1	Log2FC **6** vs **1**	T-test q-value 6 vs 1	Enriched in 6 vs 1
G3BP2	0.240	0.498	NO	0.194	0.611	NO	0.849	0.007	YES	0.856	0.001	YES	1.014	0.002	YES

c

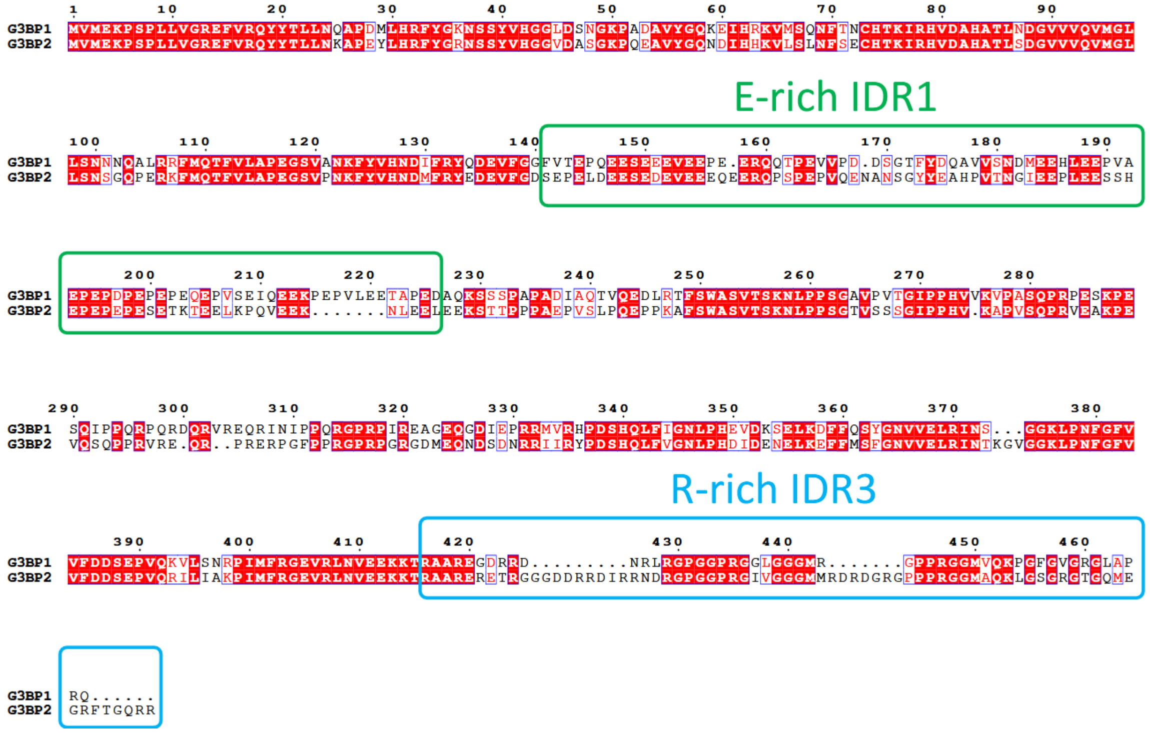

Figure S15. Ras GTPase-activating protein-binding proteins (G3BPs) are binding partners of higher polyamine analogs. **a**. Hela cells were cultured in the presence of monoamine control compound **1** (120 µM), putrescine analog **3** (30 µM), spermidine analog **4** (75 µM), or a vehicle control followed by UV irradiation at 365 nm. Probe-protein conjugates were ligated to azido-PEG3-biotin, enriched on Neutravidin beads, eluted under denaturing conditions, SDS-PAGE separated, and the levels of G3BP1 in these affinity enriched samples (Pull-down) analysed by western blotting. Additionally, G3BP1 levels were examined in samples prior to the affinity enrichment (Input). **b**. Extracted results for G3BP2 from the proteomic dataset that identified binding partners of compounds **1**-**6** (Table S1, Figure S6). Log2 fold change (Log2FC) values highlighted in green did meet while the ones highlighted in orange did not meet the statistical testing thresholds (one-sided Student's t-test, permutation-based FDR = 0.05, S0 = 0.5, n = 3) that returned protein enrichment upon treatment with probes **2**-**6** versus control compound **1**. G3BP1 was not detected in this analysis. **c.** Alignment of amino acid sequences of G3BP1 and G3BP2.

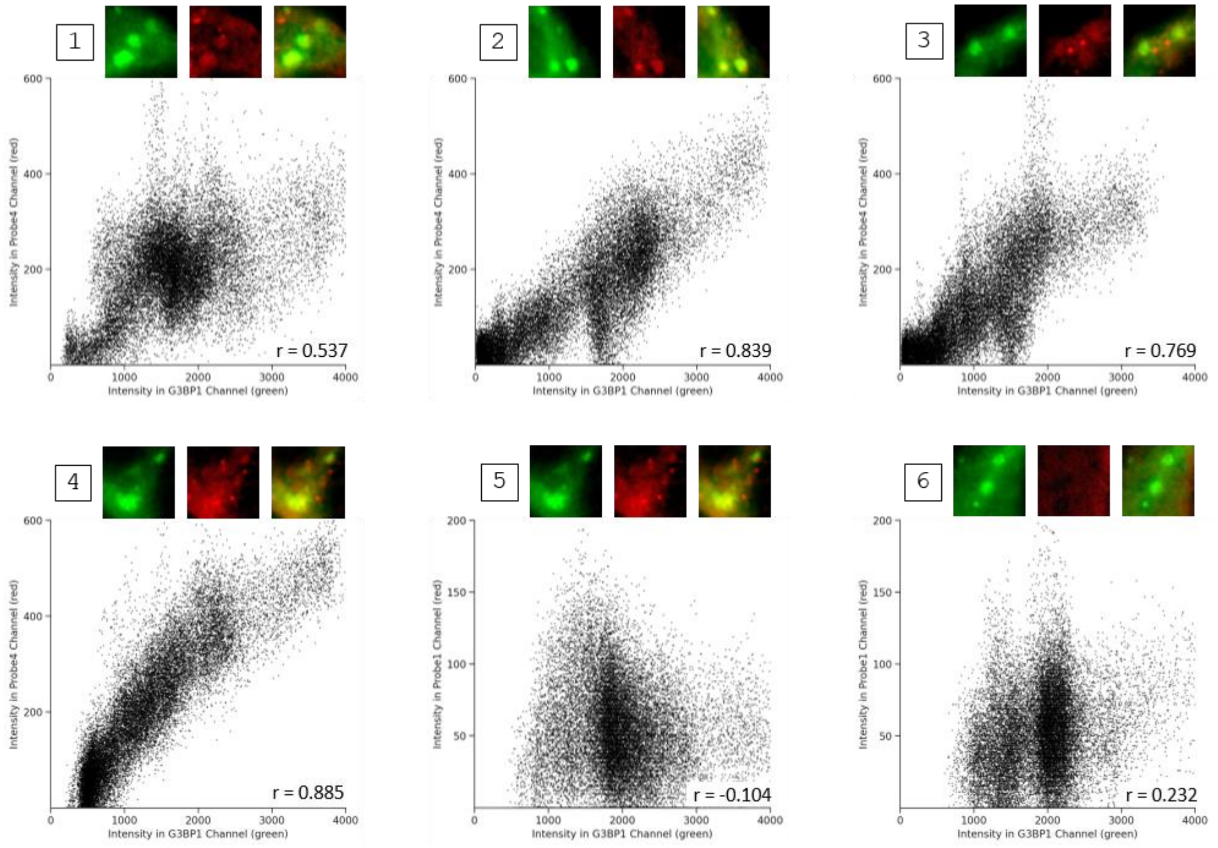

Fig S16. Scatterplots of pixel intensities from the cropped images shown in Fig. 4b, G3BP1 (green), probes **1** and **4** (red). Pearson correlation estimations (r) are shown for each plot and were quantified using Fiji plugin JACoP.[11]

4. Tables S1-5

The tables listed below can be found in the Electronic Supplementary Information in Microsoft Excel (2016) file.

Table S1 lists proteins affinity-enriched from HeLa cells following treatment with compounds **1**-**6**. In addition to the normalized reporter intensity values for individual samples, the results of statistical tests (Student's t-test, right-sided, S0 = 0.5, FDR = 0.05) are provided to identify protein groups enriched by probes **2**–**6** compared to control compound **1**. Gene names, Majority protein IDs, and Protein names are provided as identifiers. Additional outputs from MaxQuant software (MS/MS counts, Razor + unique peptide counts, and Andromeda scores) are provided as well.

Table S2 lists total levels of proteins extracted from HeLa cells treated with probes **1**, **3**, **4**, **6**, or vehicle (H_2O). In addition to the normalized reporter intensity values for individual samples, the results of statistical tests (two-sided Student's t-test, S0 = 0.1, FDR = 0.01, n = 3) are provided to identify protein groups whose levels were altered by polyamine probes **3**, **4**, and **6**, compared to either the control compound **1** or the vehicle control. Gene names, Majority protein IDs, and Protein names are provided as identifiers. Additional outputs from MaxQuant software (MS/MS counts, Razor + unique peptide counts, and Andromeda scores) are provided as well.

Table S3 (Protein subset A) contains the list of 171 protein groups bound to compounds **2** and/or **3** but not to compounds **4**–**6**. This table includes information about the primary subcellular localization of the proteins [HPA subcellular map of human proteome] and the presence of acidic stretches, defined as 20-amino-acid-long sequence fragments containing at least 10 acidic amino acids (D/E).

Table S4 (Protein subset B) contains the list 195 protein groups bound to compounds **4**, **5**, and/or **6** but not to compounds **2**-**3**. This table includes information about the primary subcellular localization of the proteins [HPA subcellular map of human proteome], the presence of acidic stretches (defined as 20-amino-acid-long sequence fragments containing at least 10 acidic amino acids, D/E), and the annotation of the proteins in MitoCarta3.0.

Table S5 lists probe-modified peptides affinity-enriched from HeLa cells after treatment with compounds **3**-**6**. For each peptide, the table reports: sequence and intensity; acidic-residue count (D/E), peptide length, monoisotopic mass, and the D/E fraction; the assigned modification based on ΔMass (putrescine +624.37815 Da, spermidine +681.43599 Da, or spermine +738.49384 Da); and identification-confidence metrics (PEP, Score, Delta Score). Gene symbols and protein names are provided as identifiers.

5. References

1. A. Wiest and P. Kielkowski, *J. Am. Chem. Soc.*, 2024, **146**, 2151–2159.
2. J. Schindelin, I. Arganda-Carreras, E. Frise, V. Kaynig, M. Longair, T. Pietzsch, S. Preibisch, C. Rueden, S. Saalfeld, B. Schmid, J. Tinevez, D. J. White, V. Hartenstein, K. Eliceiri, P. Tomancak, A. Cardona, Nat. Methods 2012, **9**, 676–682.
3. S. A. Myers, A. Rhoads, A. R. Cocco, R. Peckner, A. L. Haber, L. D. Schweitzer, K. Krug, D. R. Mani, K. R. Clauser, O. Rozenblatt-Rosen, N. Hacohen, A. Regev and S. A. Carr, *Mol Cell Proteomics*, 2019, **18**, 995–1009.
4. UniProt Consortium, *Nucleic Acids Res.*, 2023, **51**, D523–D531.
5. S. Tyanova, T. Temu and J. Cox, *Nat. Protoc.*, 2016, **11**, 2301–2319.
6. S. Tyanova, T. Temu, P. Sinitcyn, A. Carlson, M. Y. Hein, T. Geiger, M. Mann and J. Cox, *Nat. Methods*, 2016, **13**, 731–740.
7. S. X. Ge, D. Jung and R. Yao, *Bioinformatics*, 2020, **36**, 2628–2629.
8. P. J. Thul, L. Åkesson, M. Wiking, D. Mahdessian, A. Geladaki, H. Ait Blal, T. Alm, A. Asplund, L. Björk, L. M. Breckels, A. Bäckström, F. Danielsson, L. Fagerberg, J. Fall, L. Gatto, C. Gnann, S. Hober, M. Hjelmare, F. Johansson, S. Lee, C. Lindskog, J. Mulder, C. M. Mulvey, P. Nilsson, P. Oksvold, J. Rockberg, R. Schutten, J. M. Schwenk, Å. Sivertsson, E. Sjöstedt, M. Skogs, C. Stadler, D. P. Sullivan, H. Tegel, C. Winsnes, C. Zhang, M. Zwahlen, A. Mardinoglu, F. Pontén, K. von Feilitzen, K. S. Lilley, M. Uhlén and E. Lundberg, *Science*, 2017, **356**, eaal3321.
9. J. R. Conway, A. Lex and N. Gehlenborg, *Bioinformatics*, 2017, **33**, 2938–2940.
10. A. Chaudhry, R. Shi and D. S. Luciani, *Am J Physiol Endocrinol Metab*, 2020, **318**, E87–E101.
11. S. Bolte and F. P. Cordelières, *Journal of Microscopy*, 2006, **224**, 213–232.
12. Z. Li, P. Hao, L. Li, C. Y. J. Tan, X. Cheng, G. Y. J. Chen, S. K. Sze, H.-M. Shen and S. Q. Yao, *Angew. Chem. Int. Ed Engl.*, 2013, **52**, 8551–8556.
13. V. P. Singh, S. Hirose, M. Takemoto, A. M. A. S. Farrag, S.-I. Sato, T. Honjo, K. Chamoto and M. Uesugi, *J. Am. Chem. Soc.* 2024, **146**, 24, 16412–16418
14. H. Wu, J. Min, Y. Ikeguchi, H. Zeng, A. Dong, P. Loppnau, A. E. Pegg, A. N. Plotnikov, *Biochemistry*, 2007, **46**, 8331-8339.
